# Evaluating the effects of e-health interventions on mental health outcomes in individuals with breast cancer: A systematic review

**DOI:** 10.1371/journal.pone.0321495

**Published:** 2025-05-07

**Authors:** Nurdiana Mohammad Hussin, Nik Ruzyanei Nik Jaafar, Idayu Badilla Idris, Azmawati Mohammed Nawi

**Affiliations:** 1 Department of Public Health Medicine, Faculty of Medicine, Universiti Kebangsaan Malaysia, Kuala Lumpur, Malaysia; 2 Department of Psychiatry, Faculty of Medicine, Universiti Kebangsaan Malaysia, Kuala Lumpur, Malaysia; University of Maribor, SLOVENIA

## Abstract

Individuals with breast cancer (BC) often experience significant psychological distress throughout their cancer journey, and while e-health interventions show promise, evidence of their effectiveness remains limited. This systematic review addresses this gap by evaluating the effects of e-health interventions on mental health outcomes among individuals with BC. This study followed PRISMA guidelines and was registered with PROSPERO (CRD42024543722). A comprehensive search was conducted from July to August 2024, using Scopus, Web of Science, and Ovid Medline databases. Studies were included if they: (1) applied experimental study designs, (2) implemented e-health interventions to improve mental health outcomes, and (3) focused specifically on individuals with BC. Two reviewers used Rayyan software and the predefined criteria for article exclusion and inclusion. Seven studies, predominantly from countries with high BC incidence rates and advanced healthcare systems, met the inclusion criteria. Thematic analysis revealed that e-health interventions improved psychological well-being, coping strategies, quality of life, physical health, and cancer-specific symptom management. However, diverse intervention designs and measurement tools hindered cross-study comparison. Many studies focused on general mental health measures, neglecting crucial aspects such as help-seeking behaviors, cognitive function, and concerns about cancer recurrence. Future research should standardize intervention protocols, ensure comprehensive outcome reporting, and expand mental health measures.

## Introduction

Breast cancer (BC) is the most prevalent cancer and the leading cause of cancer-related death among women worldwide [1]. BC diagnosis and treatment have significant physical effects and profoundly affect patients’ psychological well-being [2]. The spectrum of mental health challenges in individuals with BC is particularly concerning, with 40–60% of patients experiencing clinically significant symptoms of anxiety and depression, especially at diagnosis and during active treatment [3]. These mental health outcomes manifest as psychological distress (including anxiety and depression), emotional dysregulation, and reduced quality of life (QOL) [4]. These psychological symptoms can be exacerbated by BC treatments such as surgery, chemotherapy, and radiotherapy, which often have burdensome side effects [5]. Prolonged distress will directly influence patients’ healthcare utilization [6] and can lead to other challenges, such as decreased QOL, sleep disturbances, and difficulty maintaining social relationships [5].

E-health interventions offer diverse features to help individuals with BC manage their psychological well-being, and include informational resources, online community support, guided relaxation exercises, and cognitive behavioral therapy programs that are accessible at any time [7,8]. These digital solutions have emerged as an effective alternative to traditional mental health services by addressing several barriers to care. Such solutions resolve geographical limitations by providing remote access to psychological support, which includes teletherapy via video or telephone [9,10]. The flexible nature of these interventions allows patients to engage with support at convenient times, which is particularly beneficial for those experiencing fluctuating energy levels and treatment side effects [10]. This digital approach is particularly beneficial to individuals with BC undergoing intensive treatments, as it minimizes their exposure to infection risks during periods of compromised immunity [11]. The high engagement of the predominantly female BC population with digital platforms further supports the suitability of these technology-based psychological interventions [12]. E-health interventions also demonstrate cost-effectiveness through their capacity to minimize in-person clinical visits and enhance resource utilization, resulting in reduced financial expenditure for both healthcare recipients and medical institutions [13].

Despite the potential benefits, the current literature on e-health implementation is fragmented across various subspecialties [14]. The proliferation of literature examining diverse e-health technologies has created a fragmented evidence landscape, complicating the process for healthcare practitioners, administrators, and decision-makers to identify and implement research findings pertinent to their contextual requirements. Additionally, healthcare providers hesitate to recommend or integrate digital interventions into their practice without clear evidence of their efficacy and adherence to established clinical guidelines [15]. This gap between the potential of e-health interventions and their implementation in clinical settings highlights the need for a more systematic approach to their development, evaluation, and integration into comprehensive BC care pathways [16].

This systematic review synthesized and evaluated the efficacy of e-health interventions in enhancing mental health outcomes among individuals with BC across diverse geographical contexts, and examined both their therapeutic impact and implementation patterns across different healthcare systems. This review synthesized evidence from diverse contexts to identify effective strategies and best practices for improving mental health outcomes for individuals with BC worldwide.

## Methods

### Protocol registration

This systematic literature review (SLR) has been registered with PROSPERO (registration ID: CRD42024543722) and was conducted as part of a clinical research project approved by the Universiti Kebangsaan Malaysia Medical Research Ethics Committee (protocol code: JEP-2024–400; approval date: July 9, 2024). The primary reference source was the 2020 Preferred Reporting Items for Systematic Literature Reviews and Meta-Analyses (PRISMA) guideline [17], which has clear guidelines on article extraction and indexing (S1 Checklist) and was instrumental in developing this SLR [18]. The most recent search for this SLR was conducted on August 2, 2024, ensuring compliance with the guidelines for timely literature review.

### Eligibility criteria

The review criteria were formulated following the PICO framework (P: population; I: intervention; C: comparison and O: outcome) which is suitable for SLRs utilizing qualitative synthesis [19]. In this SLR, P referred to the individuals with BC, I referred to the e-health interventions, C referred to existing approaches or any type of control group, and O referred to the mental health outcomes.

The eligibility criteria encompassed three primary components: (1) investigations examining e-health interventions (including digital platforms, telemedicine systems, and mobile health applications;) (2) study populations comprising individuals with BC across all treatment trajectories; and (3) research methodologies incorporating randomized controlled trials (RCTs), non-RCTs, and quasi-experimental designs (such as pre–post designs). Studies were excluded if they: (1) did not explicitly identify e-health or telemedicine as the principal intervention modality, (2) focused on physical symptoms without mental health components, or (3) were review articles, case reports, study protocols, or conference proceedings.

### Information sources and search strategy

The systematic search protocol was implemented across three major databases: Scopus, Web of Science (WoS), and Ovid Medline. WoS was selected for its subject area categorization scheme that facilitates efficient article identification, while Scopus and Medline were chosen for their comprehensive coverage of medical and open-access articles in telemedicine and oncology research [20]. The search strategy integrated three conceptual concept frameworks: (1) BC and its variants (encompassing breast neoplasm and breast malignancy), (2) telemedicine and digital health interventions (including mobile health, e-health, telehealth, and digital health platforms), and (3) mental health and healthcare-seeking behaviors (incorporating mental disorders, psychological well-being, and mental hygiene constructs).

Search terms were identified through multiple approaches, such as online searches, for related terms and synonyms, review of keywords from previous studies, and consultation with subject matter experts. We constructed comprehensive search strings using Boolean operators and wildcards, adapting the syntax for the requirements of each database. The searches were executed across titles, abstracts, and keywords between July and August 2024. To ensure comprehensive coverage, we supplemented our database searches by manually searching key journals, snowballing reference lists, and backward citation tracking of included studies. The initial search retrieved 870 articles for screening. The search strategy for each database is presented in Table 1. The full search string strategies used for each database are available in S1Table

**Table 1:** Search strategy and MeSH keywords used.

**Scopus**	TITLE-ABS ((breast AND cancer) OR (breast AND neoplasm OR (breast AND malignancy) AND (mobile AND health) OR (mhealth) OR (e-health) OR (ehealth) OR (telehealth) OR (telemedicine) OR (digital AND health) AND (help-seeking) OR (health AND seeking) OR mental AND health) OR (mental AND disorder) OR (mood AND disorder) OR (mental AND hygiene) OR well-being) OR (wellbeing) OR (psychological AND well-being) OR (mental AND help-seeking) OR (behavi*r))
**WoS**	TS=((breast AND cancer) OR (breast AND neoplasm) OR (breast AND malignancy)) AND TS=((mobile AND health) OR (mhealth) OR (e-health) OR (ehealth) OR (telehealth) OR (telemedicine) OR (digital AND health)) AND TS=((help-seeking) OR (health AND seeking) OR (mental AND health) OR (mental AND disorder) OR (mood AND disorder) OR (mental AND hygiene) OR (well-being) OR (wellbeing) OR (psychological AND well-being) OR (mental AND help-seeking) OR (behavi*r))
**Ovid Medline**	BREAST NEOPLASMS/ OR breast cancer.tw OR breast malignancy.tw AND TELEMEDICINE/ OR mobile health.tw. OR mhealth.tw. OR e-health.tw. OR ehealth.tw. OR telehealth.tw. OR telemedicine.tw. OR digital health.tw. AND MENTAL HEALTH/ OR mental disorder.tw. OR mental hygiene.tw. OR help-seeking.tw. OR health-seeking.tw. OR mood disorder.tw. OR well-being.tw. OR wellbeing.tw. OR psychological well-being.tw. OR mental help-seeking.tw. OR behaviour.tw.

### Selection process

From the initial 870 articles retrieved (WoS = 563, Scopus = 246, Ovid Medline = 61), 215 records were removed based on our inclusion criteria on the English language and original journal articles. After removing duplicates using EndNote (n = 179), the remaining 476 articles underwent a systematic screening process using Rayyan software. Two researchers (NH and AMN) independently conducted a three-tier screening approach: titles, then abstracts, and finally the method sections were reviewed against our predetermined eligibility criteria. Articles not meeting the PICO framework were excluded at each phase. Of the 476 articles, 465 were excluded due to incompatible study designs (n = 199) and irrelevant study outcomes (n = 266), leaving 11 articles from the database search.

**Fig 1:**
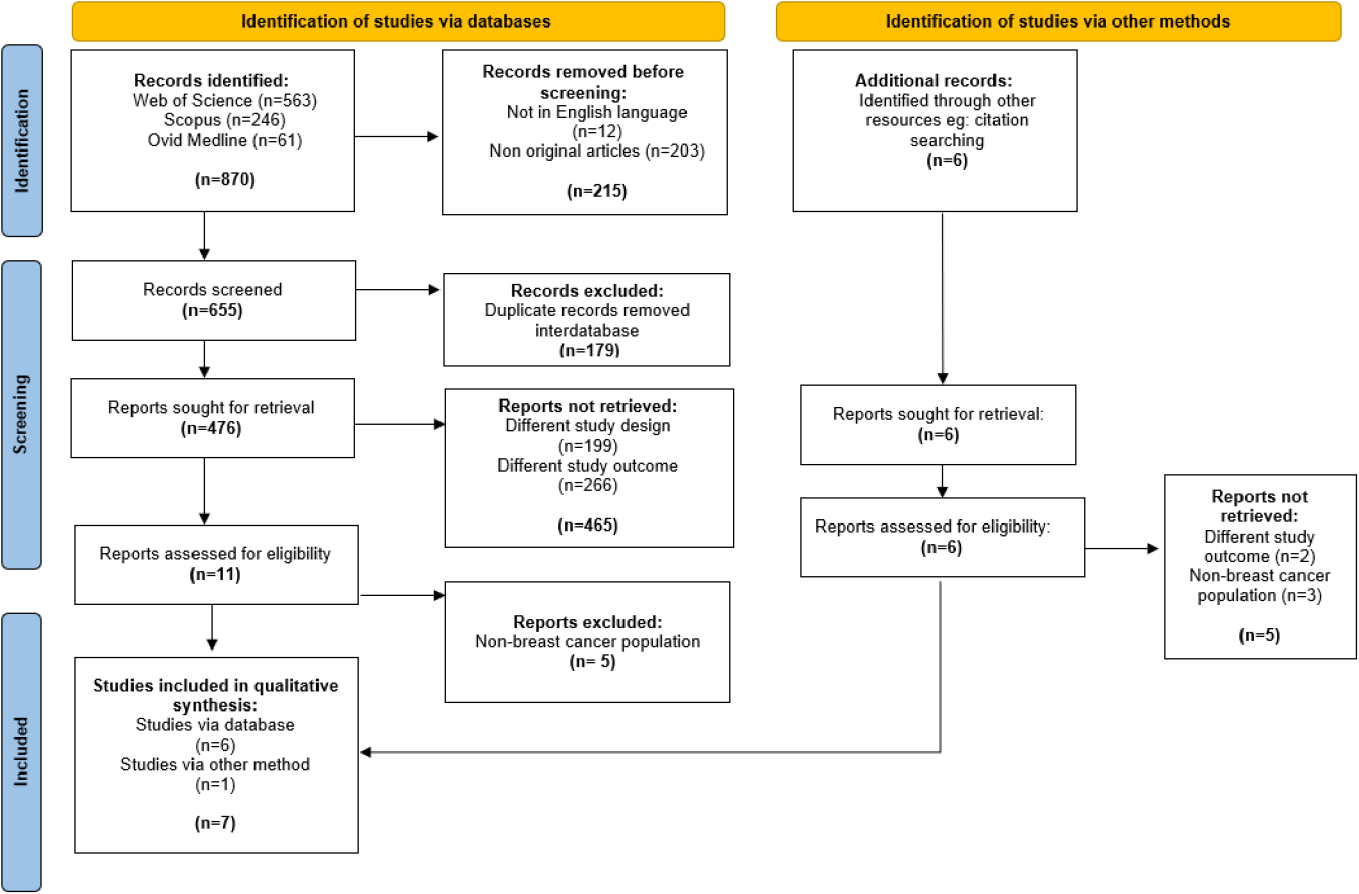
Selection process of studies according to PRISMA 2020 guideline.

Another six articles were identified through other sources, yielding a total of 17 articles for full-text review. All retrieved articles and their exclusion reasons are listed in S5 Table. The relevant data from the articles were extracted and transferred to a Microsoft Excel sheet for analysis. Authors of papers with missing data were contacted via email with specific requests for full texts or additional information, with follow-up emails sent if no initial response was received; where these were not available, abstracts were excluded. Of the 11 articles, 10 were excluded as they did not fulfil the following inclusion criteria: different study outcomes (n = 2) and non-BC populations (n = 8). Two researchers (NH, AMN) reviewed the included studies independently. Any disagreements between the two researchers were resolved through discussion, and a third researcher (IBI) was consulted when consensus could not be reached. The complete selection process is illustrated in **Fig 1**.

### Data extraction and reporting

Data were systematically extracted from the seven included articles and tabulated to provide a comprehensive overview of e-health interventions in bc care. Table 2 synthesizes the foundational study elements, which include study characteristics, design, demographics, intervention details, measurement tools, and results. S2 Table details out the data extraction process, including information about data extractors, dates of extraction, and eligibility confirmation for each included study. building upon this foundation, Table 3 presents a structured categorization of how these interventions address different aspects of bc care, where outcome measurements were organized across four primary domains: psychological well-being, physical health, qol, and cancer self-management. the intervention types and measure outcomes are further classified in S3 Table.

**Table 2:** Characteristic of studies in this review (n = 7).

No.	Author/Country (Year)	Population	Study Design	Intervention Description	Outcome Measurement (Questionnaires)	Result	Effect Category
1	Wolff et al./Germany (2023)	Treatment phase: In therapy/aftercare ≥12 weeks N = 60 Age: 49.4 years	Design: RCT Duration: 12 weeks Assessment: Baseline, 4, 8, 12 weeks	PINK! app •Multimodal content: Nutrition, Physical activity, Mental health support CG: Usual care	1) PHQ-9 2) EORTC-QLQ-C30	**Psychological Distress** **IG** showed improvement (7.6 → 5.1) **CG**: 6.9 → 6.2 (p < 0.01, d = 0.8) **Fatigue** **IG** showed improvement (51 → 41] **CG**: 48 → 47 (p < 0.01, d = 0.2)	Significant Improvement
2	Baik et al./ USA (2020)	• Treatment phase: Within 2 years post-treatment • N = 78 • Age: 52.5 years	Design: Pilot RCT Duration: 8 weeks Assessment: Baseline, 6, 8 weeks	My Guide app Psychosocial support Education CG: My Health app (general health)	HRQOL 1) PHQ-9 2) BCPT 3) IES 4) CASE-cancer 5) BC knowledge	HRQOL *Physical well-being **IG** showed worse outcomes (21.23 → 20.27) **CG** showed no improvement (20.21 → 20.00) *Emotional well-being **IG** showed worse outcomes (19.50 → 18.60) **CG** showed slight improvement (18.89 → 19.00) *Functional well-being **IG** showed worse outcomes (20.73 → 19.95) **CG** showed slight improvement (20.42 → 21.06) *Social well-being **IG** showed worse outcomes (21.02 → 20.90) **CG** showed slight improvement (20.74 → 22.52) *BC well-being **IG** showed worse outcomes (23.5 → 23.41) **CG** showed slight improvement (21.37 → 22.60) †**Symptom burden** **IG** showed worse outcomes (25.55 → 25.14) **CG** showed improvement (31.32 → 27.53) †**Cancer-specific distress** **IG** showed worse outcomes (22.50 → 23.27) **CG** showed improvement (29.58 → 24.35) *Cancer-relevant self-efficacy **IG** showed worse outcomes (44.05 → 43.05) **CG** showed improvement (43.00 → 43.81) *Breast cancer knowledge **IG** showed improvement (9.14 → 11.82) **CG** showed slight improvement (9.21 → 9.65)	No Significant Change
3	Chow et al./ USA (2020)	Treatment phase: Active treatment N = 40 Age: 56.8 years	Design: Pre-Post Duration: 7 weeks Assessment: Baseline, 7 weeks	IntelliCare app CBT Mindfulness • Positive psychology Support calls & SMS	1) PHQ-4 2) PROMIS	†**General distress** Improved from 3.96 to 2.83 (p = 0.02) †**Anxiety symptoms** Improved from 60.26 to 56.53 (p = 0.05) †**Depression symptoms** Improved from 53.77 to 51.09 (p = 0.03) ᵇHigher scores indicate worse outcomes	Significant Improvement
4	Villani et al./ Italy (2018)	Treatment phase: Active treatment N = 29 Age: 62.76 years	Design: RCT • Duration: 12 weeks Assessment: Baseline, 2, 12 weeks	SIT program Stress management • Relaxation Cognitive restructuring CG: Usual care	1) ERQ 2) FACT-B	**ERQ** **Emotional Suppression** **IG** showed slight improvement (2.93 → 2.33) **CG** showed worse outcomes (3.63 → 4.15) **Cognitive Reappraisal** **IG** showed slight improvement (4.99 → 5.43) **CG** showed slight improvement (4.53 → 4.88) **FACT-B** **Physical well-being** **IG** showed worse outcomes (24.40 → 21.92) **CG** showed worse outcomes (21.43 → 19.82) **Social well-being** **IG** no improvement (18.73 to 18.62) **CG** showed slight improvement (16.21 → 17.55) **Emotional well-being** **IG** showed improvement (17.60 → 19.54) **CG** showed slight improvement (16.43 → 16.82) **Functional well-being** **IG** no improvement (14.13 → 14.31) **CG** showed slight improvement (12.64 → 13.45)	No Significant Change
5	Meneses et al./USA (2018)	Treatment phase: Within 3 years post-treatment N = 40 Age: 56.63 years	Design: RCT • Duration: 24 weeks Assessment: Baseline, 12, 24 weeks	Support Intervention 3 weekly education sessions 6 support calls in first month CG: Delayed education	1) PCS 2) MCS 3) CES-D	*PCS Slight improvement from 46.02 → 47.1 (d = 0.1), but remained below population mean of 50 †**MCS** Initial improvement at T2 (48.77 → 50.29, d = 0.14), Declined slightly at T3 (49.36, d = 0.06) †**CES-D** Slight worsening at T2 (13.68 → 14.75, d = 0.08) Returned to baseline at T3 (13.81, d = 0.01) All scores remained below clinical threshold (CES-D ≥ 16)	No Significant Change
6	Kuijpers et al./The Netherlands (2016)	Treatment phase: During or within 1 year post-treatment • N = 92 Age: 49.5 years	Design: Pre-Post Duration: 16 weeks Assessment: Baseline, 16 weeks	**MijnAVL Portal** Personalized education Appointment tracking EMR access Physical activity support	1) PAM 2) SF-36 3) IPAQ	**PAM** Slight decline from 62.7 to 60.9 (not significant) **SF-36** **Self-efficacy** Increased from 65.3 to 78.5 (p = 0.021) **Mental health** Improved from 69.8 to 76.5 (p < 0.01) **Social functioning** Enhanced from 71.2 to 80.5 (p < 0.01) **IPAQ** **Vigorous activity** Significant improvement from 0 to 360 MET-min/week (p = 0.017)	Significant improvement
7	Børøsund et al./Norway (2014)	Treatment phase: Active treatment N = 176 WebChoice: 51 IPCC: 50,	Design: RCT • Duration: 24 weeks Assessment: Baseline, 8, 16, 24 weeks	**WebChoice** Web-based support Symptom monitoring Self-management Patient communication **IPCC** Secure messaging only CG: Usual care	1) MSAS 2) HADS 3) CBI	**Symptom distress** **WebChoice** Significant improvement (-0.16, p = 0.001) **IPCC** No significant change (-0.07, p = 0.21) **Anxiety** **WebChoice** Significant improvement (-0.79, p = 0.03) **IPCC** No significant change (-0.14, p = 0.72) **Depression** **WebChoice** Significant improvement (-0.61, p = 0.03) **IPCC** Similar improvement (-0.69, p = 0.03) **Self-Efficacy** **WebChoice** Positive trend but not significant (8.81, p = 0.08) **IPCC** No improvement (-4.89, p = 0.38)	Significant improvement in WebChoice
		Control: 53 Age: NR					

CG: Control Group; IG: Intervention Group; T1: First assessment; T2: Second assessment; T3: Third assessment; T4: Fourth assessment

Patient Health Questionnaire-9 (PHQ-9), European Organization for Research and Treatment of Cancer Quality of Life Questionnaire Core 30 (EORTC-QLQ-C30), Health-Related Quality of Life (HRQOL), Breast Cancer Prevention Trial Questionnaire (BCPT), Impact of Event Scale (IES), Communication and Attitudinal Self-Efficacy scale for cancer (CASE-cancer), Patient Health Questionnaire-4 (PHQ-4), Patient-Reported Outcomes Measurement Information System (PROMIS), Emotion Regulation Questionnaire (ERQ), Functional Assessment of Cancer Therapy-Breast (FACT-B), Physical Component Score (PCS), Mental Component Score (MCS), Center for Epidemiologic Studies Depression Scale (CES-D), Patient Activation Measure (PAM), 36-Item Short Form Health Survey (SF-36), International Physical Activity Questionnaire (IPAQ), Memorial Symptom Assessment Scale (MSAS), Hospital Anxiety and Depression Scale (HADS), Cancer Behavioral Inventory (CBI)

* Higher scores reflect better outcomes, †Higher scores reflect worse outcomes

**Table 3:** Domains and outcomes of e-health intervention in BC care.

Domain	Subdomain	Outcome Measurement	Effect Category	Study	Intervention
Psychological Well-Being	Distress	PHQ-9†	Significant Improvement	Wolff et al. (2023)	Mobile Apps
		PHQ-4†	Significant Improvement	Chow et al. (2020)	Mobile Apps
		IES†	Negative Outcomes	Baik et al. (2020)	Mobile Apps
		MSAS†	Significant Improvement	Børøsund et al. (2014)	Web-Based Portal
	Depression	PROMIS†	Significant Improvement	Chow et al. (2020)	Mobile Apps
		HADS†	Significant Improvement	Børøsund et al. (2014)	Web-Based Portal
		CES-D†	No Significant Change	Meneses et al. (2018)	Web-Based Portal
	Anxiety	PROMIS†	Significant Improvement	Chow et al. (2020)	Mobile Apps
		HADS†	Significant Improvement	Børøsund et al. (2014)	Web-Based Portal
	Self-Efficacy	SF-36*	Significant Improvement	Kuijpers et al. (2016)	Web-Based Portal
		CBI*	No Significant Change	Børøsund et al. (2014)	Web-Based Portal
		CASE*	Negative Outcomes	Baik et al. (2020)	Mobile Apps
	Emotional Suppression	ERQ†	No Significant Change	Villani et al. (2018)	Digital Delivery
		PHQ-9†	Negative Outcomes	Baik et al. (2020)	Mobile Apps
	Mental Health	SF-36*	Significant Improvement	Kuijpers et al. (2016)	Web-Based Portal
		MCS†	No Significant Change	Meneses et al. (2018)	Web-Based Portal
	Empowerment	PAM*	Negative Outcomes	Kuijpers et al. (2016)	Web-Based Portal
	Cognitive Appraisal	ERQ*	No Significant Change	Villani et al. (2018)	Digital Delivery
Physical Health	Physical Well-being	FACT-B*	Negative Outcomes	Villani et al. (2018)	Digital Delivery
		PCS*	No Significant Change	Meneses et al. (2018)	Web-Based Portal
		PHQ-9†	Negative Outcomes	Baik et al. (2020)	Mobile Apps
	Fatigue	EORTC-QLQ-C30†	Significant Improvement	Wolff et al. (2023)	Mobile Apps
	Symptom Burden	BCPT†	No Significant Change	Baik et al. (2020)	Mobile Apps
QOL	Social Well-Being	BCPT*	Negative Outcomes	Baik et al. (2020)	Mobile Apps
		FACT-B*	No Significant Change	Villani et al. (2018)	Digital Delivery
		SF-36*	Significant Improvement	Kuijpers et al. (2016)	Web-Based Portal
	Functional Well-Being	BCPT*	Negative Outcomes	Baik et al. (2020)	Mobile Apps
		FACT-B*	No Significant Change	Villani et al. (2018)	Digital Delivery
Cancer Self-Management	BC Well-being	BCPT*	Negative Outcomes	Baik et al. (2020)	Mobile Apps
	Knowledge regarding BC	BC Knowledge*	Significant Improvement	Baik et al. (2020)	Mobile Apps

†Higher scores reflect worse outcomes, * Higher scores reflect better outcomes.

**Fig 2:**
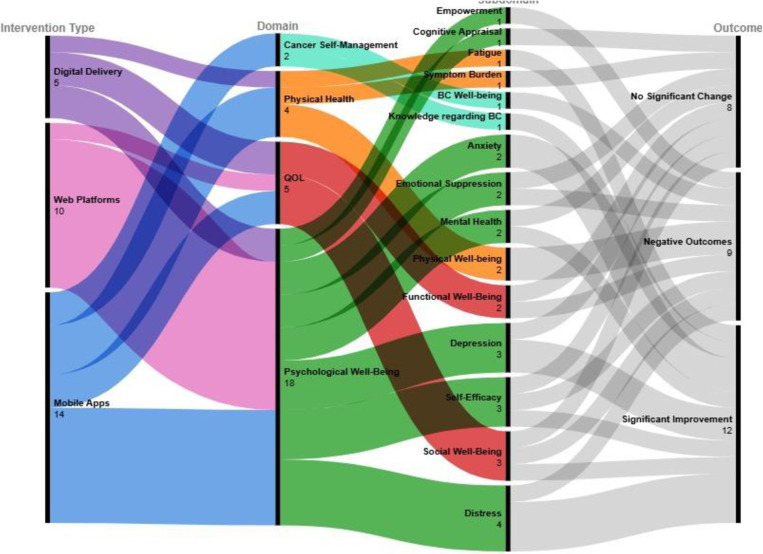
Patterns of digital intervention types and their outcomes in BC care.

These complex relationships were visualized using an alluvial chart (Fig 2.) that maps the flow patterns between intervention types (mobile apps, web-based platforms, and digital delivery), domains, subdomains, and their corresponding outcomes. this visualization approach built upon earlier systematic reviews [21] and provided insights into both the distribution and effectiveness of different delivery methods across various aspects of bc care. the dataset used to create the alluvial chart is presented in S3 Table.

## Results

### Study characteristics

The included studies were experimental: four RCTs [22–25], two pre–post studies [7,26], and one pilot RCT [27]. The sample size ranged from 29 [23] to 92 [26] with an average sample size of 56 and the mean participant age was 49.4–62.76 years. The interventions lasted between 7 [7] and 24 [24] weeks. The studies involved individuals with BC at various treatment and recovery stages.

The included studies evaluated the effectiveness of e-health interventions with the Patient Health Questionnaire (PHQ-9) [22] and the Hospital Anxiety and Depression Scale (HADS) [25] for distress, anxiety, and depression; the European Organization for Research and Treatment of Cancer QOL Questionnaire-Core 30 (EORTC QLQ-C30) [22] and the Functional Assessment of Cancer Therapy – Breast (FACT-B) [23,27] for QOL; the Impact of Event Scale (IES) [27] for stress and coping; and the Short-Form 36-Item Health Survey (SF-36) [26] for social support and self-efficacy.

The interventions were delivered primarily through mobile apps [7,22,27] and web-based portals [24–26] that included multi-format content (articles, videos, podcasts) [22], personalized educational materials [26], and symptom monitoring tools [25] to support and educate. Additionally, one intervention enhanced digital delivery by incorporating phone-based coaching or education sessions [23].

### Effectiveness of e-health interventions

The analysis of the intervention types revealed distinct patterns in their application and effectiveness across four main domains **(Fig 2).** Each intervention type demonstrated unique strengths and limitations in addressing different aspects of BC care.

Mobile apps were tools with varying effectiveness across domains. In the psychological well-being domain, mobile apps demonstrated clinically meaningful and statistically significant positive outcomes: the PINK! app [22] demonstrated significant improvement in distress management with both statistical significance and clinical relevance (7.6–5.1, p < 0.01) supported by a large effect size (d = 0.8), while the IntelliCare app [7] demonstrated clinically meaningful reductions in distress (3.96–2.83, p = 0.05), anxiety (60.26–56.53, p = 0.05), and depression symptoms (53.77–51.09, p = 0.03), with scores maintained below clinical thresholds. Mobile apps yielded mixed results in the physical health domain. While the PINK! app [22] demonstrated significant improvement in fatigue management with a small but clinically relevant effect size (51–41, p < 0.01, d = 0.2), other physical well-being measures demonstrated negative outcomes. Mobile apps performed poorly for QOL outcomes, with the My Guide app [27] demonstrating clinically significant declines across social well-being (21.02–20.90), emotional well-being (19.50–18.60), and functional well-being (20.73–19.95). In the cancer self-management domain, mobile apps achieved significant improvement in BC knowledge (9.14–11.82) through the My Guide app [27], but demonstrated negative outcomes in BC well-being.

Web-based platforms demonstrated comprehensive effectiveness through their integrated approach. In the psychological well-being domain, the WebChoice platform [25] demonstrated clinically meaningful and statistically significant improvements across multiple subdomains: symptom distress (-0.16, p = 0.001), anxiety (-0.79, p = 0.03), and depression (-0.61, p = 0.03), with changes exceeding established thresholds for minimal clinically important differences. Similarly, the MijnAVL portal [26] achieved notable success across psychological well-being subdomains, enhancing mental health (69.8–76.5, p < 0.01) and self-efficacy (65.3–78.5, p = 0.021) while improvements in the QOL domain through enhanced social functioning (71.2–80.5, p < 0.01) demonstrated statistical significance and clinical relevance. However, web-based platforms demonstrated no significant measurements in the cancer self-management domain, suggesting a potential gap.

Digital delivery systems, represented by the SIT program [23], demonstrated limited effectiveness across most domains. In the psychological well-being and QOL domains, these systems demonstrated no significant changes across multiple dimensions, including emotional suppression, cognitive appraisal, social well-being (18.73–18.62), and functional well-being (14.13–14.31). In the physical health domain, digital delivery systems demonstrated consistent deterioration in physical well-being scores.

These results indicate that each intervention type serves distinct purposes in BC care. Despite negative outcomes in QOL measures mobile apps excelled in immediate symptom management and educational support, particularly in improving disease-specific knowledge and managing psychological symptoms such as distress, anxiety, and depression. Web-based platforms provided comprehensive care management with broader positive outcomes across multiple domains, demonstrating particular strength in psychological well-being and QOL outcomes. Digital delivery systems demonstrated limited effectiveness, with no significant improvements in psychological well-being and QOL domains, and deterioration in physical well-being.

**Fig 3:**
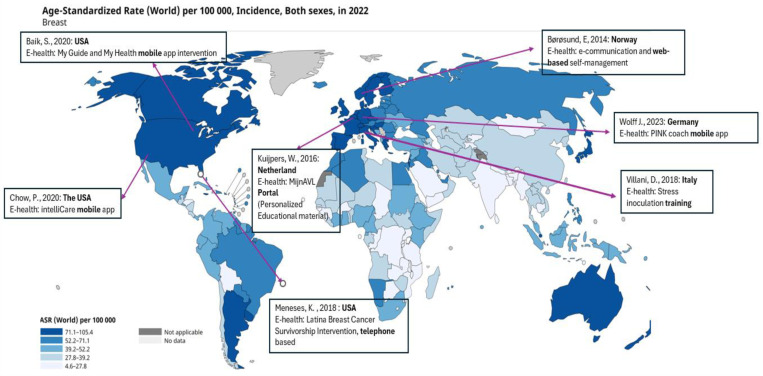
Global BC incidence and intervention research sites.

**Fig 3** depicts the age-standardized incidence rate (ASR) of BC globally in 2022, which highlighted the implementation of e-health interventions across different geographic regions. The incidence rates varied significantly, where the highest figures were recorded in North America, Australia, and much of Europe, whereas the rates were lowest in Asia and Africa.Innovative applications such as My Guide, My Health, and IntelliCare were developed to support BC management in high-incidence areas such as the United States. The Netherlands introduced the MijnAVL Portal, a personalized e-health solution, while Germany created the PINK! Coach mobile app. The map also highlights a significant e-health project in Norway: a web-based system facilitating self-management and communication among individuals with BC. In Italy, researchers implemented a stress inoculation training (SIT) program to provide psychological support to individuals with BC.

### Study characteristics and outcomes

The relationship between the participant numbers and study outcomes revealed a varied landscape across the reviewed studies. The studies ranged from smaller-scale investigations with 29–40 participants to larger trials involving up to 176 individuals, each contributing unique insights into the efficacy of e-health interventions. The smaller studies (29–40 participants) demonstrated promising improvements in specific areas, although statistical significance was not always achieved or reported. For example, a 29-participant study reported that the intervention group had improved coping strategies, particularly in reducing emotional suppression and enhancing cognitive appraisal [23]. Another small-scale study (40 participants) reported significant reductions in psychological well-being, which included distress, anxiety, and depression symptoms [7]. These results suggested that targeted interventions can yield meaningful benefits even with limited participant numbers, particularly in psychological well-being and coping strategies.

Medium-sized studies (60–92 participants) also reported noteworthy results. A 60-participant study reported significant improvements in distress symptoms and fatigue levels (both outcomes: p < 0.01), with moderate to large effect sizes (depression: d = 0.8; fatigue: d = 0.2) [22]. In a 92-participant study, the SF-36 revealed significantly enhanced self-efficacy (p < 0.01), mental health (p < 0.01), and social well-being (p < 0.01). These results indicated that the interventions in the medium-sized studies were effective in addressing psychosocial outcomes ranging from specific symptoms such as depression and fatigue to broader QOL measures. The largest study (176 participants) provided additional insights into intervention efficacy. Significantly improved psychological well-being was reported for the WebChoice intervention group. The IPCC intervention group also demonstrated significantly improved depression symptoms (p = 0.03) [25].

The reviewed studies revealed a complex interplay between study duration and intervention outcomes. The interventions ranged 7–24 weeks, and assessment points varied across studies, allowing for a nuanced examination of immediate and sustained effects. Shorter interventions (7–8 weeks) yielded significantly improved psychological well-being [7]. Similarly, an 8-week study reported improvements across various health-related QOL (HRQOL) domains, although statistical significance was not reported for all outcomes [27]. Medium-length interventions (12 weeks) presented varied results. One study reported significantly improved distress symptoms (PHQ-9 scores) and fatigue levels (both outcomes: p < 0.01), with moderate to large effect sizes (depression: d = 0.8; fatigue: d = 0.2) [22]. Another 12-week study reported improved emotional regulation and QOL measures but did not provide p-values, rendering assessment of the statistical significance of these changes challenging [23]. Longer interventions (16–24 weeks) tended to yield more sustained, albeit variable, improvements. The 16-week intervention yielded significantly enhanced self-efficacy (p < 0.01), mental health (p < 0.01), and social functioning (p < 0.01) [26]. The 24-week studies [24,25] reported improvements for various outcomes, such as symptom distress, anxiety, and depression. However, the relationship between duration and outcome was not always linear. For example, some measures yielded slight improvements at 12 weeks, which plateaued or decreased slightly by 24 weeks [24]. Interestingly, the outcomes varied even within studies of similar duration. This was evident in the 24-week studies [24,25], where different intervention types (WebChoice vs. IPCC) demonstrated varying effectiveness for different outcomes [25]. This result suggested that factors other than intervention duration, such as intervention type and content, are crucial in determining outcomes.

This SLR examined e-health intervention efficacy on mental health outcomes across different treatment and recovery stages in individuals with BC, enabling the analysis of how these stages might influence their outcomes. Individuals actively receiving cancer treatment demonstrated significantly reduced psychological well-being [7]. Similarly, individuals undergoing active treatment had improved emotional suppression, although the specific p-values were not reported [23]. Patients in therapy or aftercare for at least 12 weeks also had significantly improved distress scores (p < 0.01) [22], suggesting that digital interventions can be effective even beyond the active treatment phase. For patients who had recently completed treatment (within 2–24 months), the results varied with the level of app usage [27]. In longer-term recovery, patients demonstrated slight improvements in mental component scores (MCS) within 3 years of completing primary treatment, although the effect size was small (d = 0.06) [24]. These results suggested that digital interventions may positively affect mental health outcomes across various BC treatment and recovery stages.

### Quality rating of included studies

The study quality was evaluated using the Effective Public Health Practice Project Quality Assessment Tool (EPHPP), which was introduced in 1998 and has been a valid and reliable instrument for systematic reviews focusing on public health effectiveness [20]. The EPHPP demonstrates fair inter-rater agreement for individual component ratings and excellent agreement for final ratings [28]. The assessment involved examining bias across six component areas, where each study received a global rating of “strong”, “moderate”, or “weak”. The researchers (NH, AMN, NRNJ, IBI) independently reviewed all selected articles, then collaborated to agree on the final rating for each study. The EPHPP quality rating determined that four studies were strong [22,23,25,27], one study was moderate [24], and two studies were weak [7,26] (Table 4). Among the quality rating criteria, two studies were rated double weak for selection bias due to their recruitment strategies and the assessors’ awareness of whether participants were in the intervention or control group. The detailed component question answers for each domain assessed in the EPHPP risk of bias assessment are presented in S4Table.

**Table 4:** EPHPP risk of bias assessment.

Study ID	Selection Bias	Study Design	Confounders	Blinding	Data Collection Methods	Withdrawals and Dropouts	Global Rating
Wolff et al. (2023)	Moderate	Strong	Strong	Moderate	Strong	Strong	Strong
Baik et al. (2020)	Moderate	Strong	Strong	Moderate	Strong	Strong	Strong
Chow et al. (2020)	Moderate	Weak	Weak	Weak	Strong	Weak	Weak
Meneses et al. (2018)	Moderate	Strong	Strong	Moderate	Strong	Weak	Moderate
Villani et al. (2018)	Moderate	Strong	Strong	Moderate	Strong	Strong	Strong
Kuijpers et al. (2016)	Moderate	Weak	Weak	Weak	Strong	Strong	Weak
Børøsund et al. (2014)	Moderate	Strong	Strong	Moderate	Strong	Strong	Strong

## Discussion

This systematic review of seven experimental studies reveals the complex landscape of e-health interventions in BC care, highlighting promising advances and important limitations across different delivery modalities. Our results demonstrate a nuanced effectiveness pattern that varied significantly by intervention type and outcome domain, suggesting the need for a more targeted approach in e-health intervention design and implementation.

The superior performance of web-based platforms in managing psychological well-being and QOL outcomes represented a significant advancement in e-health intervention design. The comprehensive success of platforms such as WebChoice [25] in reducing symptom distress (p = 0.001), anxiety (p = 0.03), and depression (p = 0.03), coupled with the improvements by MijnAVL [26] in mental health (p < 0.01) and social functioning (p < 0.01), suggests that integrated, multi-feature approaches may be more effective than single-focus interventions. This result aligns with research emphasizing the importance of holistic support systems in cancer care management [29].

Mobile applications demonstrated a distinct pattern of effectiveness, excelling in specific domains while exhibiting limitations in others. The success of the PINK! app [22] in distress management (p < 0.01, d = 0.8) and fatigue reduction (p < 0.01, d = 0.2) and the positive effects of IntelliCare [7] on anxiety (p = 0.05) and depression (p = 0.03) suggest their utility for targeted symptom management. However, the negative outcomes in QOL domains of the My Guide app, particularly in social, emotional, and functional well-being [27], indicated that mobile apps might be better suited as complementary tools within a broader care framework rather than standalone solutions [30].

One notable result was the differential effectiveness across intervention types and domains. While web-based platforms demonstrated comprehensive improvements across psychological and QOL domains, mobile apps demonstrated stronger outcomes in specific symptom management and knowledge improvement. This pattern suggests that different e-health modalities might have distinct purposes in the cancer care continuum, with web-based platforms better suited to integrated care management and mobile apps for targeted intervention delivery [31].

The consistent pattern of limited effectiveness in physical health domains across intervention types, except for fatigue management in mobile apps, highlighted a significant gap in current e-health approaches. This finding result the need for innovative strategies that might combine digital interventions with traditional physical rehabilitation approaches, particularly by integrating wearable technology and real-time monitoring systems. The positive outcomes in fatigue management through mobile apps [22] (d = 0.2) provide a potential model for future development in physical health support.

The varied effectiveness in cancer self-management domains presents another crucial consideration in e-health intervention development. While mobile apps significantly improved BC knowledge (9.14–11.82), their negative outcomes in BC well-being highlight a fundamental challenge in digital health interventions: the knowledge–behavior gap. This phenomenon, where increased disease knowledge fails to translate into improved health outcomes, has been increasingly recognized in digital health literature [32]. The disconnect between knowledge improvement and practical outcomes aligns with recent systematic reviews suggesting that the provision of information alone, even using sophisticated digital platforms, may be insufficient for meaningful behavioral change in individuals with cancer [33].

Intervention duration and timing were critical factors influencing the outcome. Brief interventions spanning 7–12 weeks yielded accelerated improvements in psychological well-being, as demonstrated by outcomes from the success of the PINK! app [22] in distress management and the positive effects of IntelliCare [7] on anxiety and depression. These outcomes were particularly valuable for immediate post-diagnosis support. Conversely, longer interventions (16–24 weeks) yielded more sustained benefits across broader outcomes, as demonstrated by the improvements by WebChoice [25] and MijnAVL [26] in emotional suppression and self-efficacy. However, this relationship is not strictly linear, with some studies reporting plateaued or slightly decreased improvements during longer interventions, suggesting the need for personalized intervention durations based on specific outcomes and patient characteristics.

The analysis revealed that participants receiving active treatment demonstrated the most substantial therapeutic gains, exhibiting statistically significant improvements in psychological well-being outcomes. This finding aligned with previous review results demonstrating that e-health interventions are particularly effective during the acute phase of cancer care [34]. In particular, the success of the WebChoice platform [25] in reducing symptom distress during active treatment phase supports this observation.

While mental health outcomes also improved significantly during the post-treatment phase [22], the effects tended to diminish in extended survivorship, potentially due to reduced intervention adherence or changing psychosocial needs over time [35]. This pattern suggests the need for stage-specific tailoring of digital support strategies, possibly through adaptive intervention algorithms that adjust content and engagement strategies based on the individual’s treatment phase and psychological needs.

Our analysis revealed important gaps in comprehensive mental health assessment, with many studies focusing on general measures such as the HADS and PHQ-9 while overlooking specific aspects such as sleep quality, cognitive function, body image, self-esteem, and concerns about cancer recurrence. Additionally, the e-health intervention efficacies appeared to vary across different patient demographics, with the sample age range (49.4–62.76 years) indicating potential selection bias. Older individuals often face greater challenges in adopting technology [15], while younger individuals demonstrate higher receptivity to app-based interventions [25]. These heterogeneous responses emphasize the need for tailored approaches in e-health interventions.

Geographic disparities in e-health intervention development and implementation emerged as a significant concern. While higher-income regions, particularly North America and Europe (each represented by three studies), lead in developing and implementing these interventions, lower-resource settings often lack access to such innovative digital health solutions despite the increasing BC burdens. This disparity highlights the urgent need for culturally appropriate and resource-sensitive interventions for diverse global contexts, particularly through apps that function with slow internet connections, inconsistent internet access, and that can be used easily on basic mobile phones.

These results have significant implications for clinical implementation and future research directions. Healthcare providers should consider adopting a mixed-modality approach, combining the comprehensive care capabilities of web-based platforms with the targeted symptom management features of mobile apps. Future research should prioritize several key areas to advance the field of e-health interventions in BC care. The standardization of outcome measures across investigations was considered crucial for facilitating robust comparative analyses and meta-analytic studies, while longitudinal follow-up investigations would provide critical insights into the intervention effect durability beyond the immediate treatment phase. There is also a pressing need to develop culturally adaptive interventions that can effectively serve diverse populations and address the current geographical and cultural disparities in e-health implementation. Investigating the optimal intervention durations for different outcomes and patient subgroups would enable more personalized and effective intervention designs. Additionally, research should focus on developing and evaluating strategies to maintain long-term engagement, particularly in extended survivorship phases, where intervention effects tend to diminish. This comprehensive research agenda would contribute to more effective, sustainable, and inclusive e-health interventions for individuals with BC across the care continuum.

## Limitations

Our SLR has several limitations. First, the heterogeneity of intervention designs and measurement tools across studies made direct comparisons challenging and potentially contributed to inconsistent results. Second, the modest number of studies included (n = 7) and their relatively limited sample sizes (29–92 participants) potentially limited the generalizability of the results. Third, the lack of long-term follow-up data in many studies restricted our understanding of the sustained effects of these interventions beyond the immediate intervention period. Fourth, the geographical bias towards high-income countries with advanced medical resources may limit the applicability of the results to diverse global settings, particularly resource-limited areas. Finally, the lack of standardized reporting of intervention components and implementation strategies across the studies hindered our ability to identify specific features contributing to intervention success.

## Conclusion

This systematic review demonstrated the potential and limitations of e-health interventions in supporting individuals with BC. Our results reveal the specific effectiveness of these interventions in reducing distress, depression, and anxiety symptoms while improving physical well-being and social functioning. Web-based platforms were especially effective in providing comprehensive support, while mobile apps were valuable for targeted symptom management.

However, the varying effectiveness across different domains and intervention types underscored an urgent need for standardization in intervention protocols and outcome measurements across future studies. The current variability in assessment tools and methodological approaches significantly limited our ability to compare interventions effectively and establish best practices in e-health delivery. Future successful implementation of e-health interventions in BC care necessitates three key elements: (1) the establishment of standardized protocols and validated outcome measures, (2) culturally informed adaptation of interventions to address the needs of diverse populations, and (3) the integration of multiple delivery modalities to ensure comprehensive therapeutic support across the care continuum.

Despite some inconsistent results, the evidence demonstrates substantial potential for e-health interventions in cancer care. Subsequent research initiatives should prioritize addressing current limitations through three primary mechanisms: the establishment of extended longitudinal follow-up studies, ensuring comprehensive reporting of all study results, and the development of culturally responsive interventions that can be deployed effectively across varied global healthcare settings. Through these improvements, e-health interventions can better fulfill their potential in providing accessible, effective support for individuals with BC across the care continuum.

## Supporting information

S1 Table Full search string strategies. Detailed search strategies used for Scopus, Web of Science, and Ovid Medline databases, including all search terms, Boolean operators, field codes, and document filtering criteria applied at each stage of the identification, screening, and eligibility assessment.
(DOCX)

S2 Table Data extraction details and eligibility confirmation. Comprehensive data extraction table containing study details, intervention characteristics, outcomes, effect categories, eligibility confirmation, and information about data extractors with extraction dates for each included study.
(DOCX)

S3 Table Intervention types and measure outcomes. Detailed categorization of digital interventions by type (mobile apps, web platforms, digital delivery), organized by domains (psychological well-being, physical health, quality of life, cancer self-management), subdomains, and their corresponding outcomes.
(DOCX)

S4 Table Quality assessment tool for quantitative studies. Detailed evaluation of all included studies using the Effective Public Health Practice Project (EPHPP) quality assessment tool, showing ratings for selection bias, study design, confounders, blinding, data collection methods, withdrawals and dropouts, with component question answers and global quality ratings.
(DOCX)

S5 Table Retrieved articles and exclusion reasons. List of articles identified through database searches and other sources, including bibliographic information and specific reasons for exclusion where applicable.
(DOCX)

S1 Checklist PRISMA 2020 checklist. Detailing required components for transparent reporting of systematic reviews across title, abstract, introduction, methods, results, discussion, and other information sections.
(DOCX)
