# Supplementary material for: Evaluating the effects of e-health interventions on mental health outcomes in individuals with breast cancer: A systematic review
Source: PLoS One. 2025 May 7;20(5):e0321495. doi: 10.1371/journal.pone.0321495 (PMC12057970; doi:10.1371/journal.pone.0321495)
Supplement: S1 Table — Detailed search strategies used for Scopus, Web of Science, and Ovid Medline databases, including all search terms, Boolean operators, field codes, and document filtering criteria applied at each stage of the identification, screening, and eligibility assessment. (DOCX) [file pone.0321495.s001.docx]

**S1 Table:** Full search string strategies

| **Scopus** | Identification  (246 article) | TITLE-ABS ( ( breast AND cancer ) OR ( breast AND neoplasm ) OR ( breast AND malignancy ) AND ( mobile AND health ) OR ( mhealth ) OR ( e-health ) OR ( ehealth ) OR ( telehealth ) OR ( telemedicine ) OR ( digital AND health ) AND ( help-seeking ) OR ( health AND seeking ) OR ( mental AND health ) OR ( mental AND disorder ) OR ( mood AND disorder ) OR ( mental AND hygiene ) OR ( well-being ) OR ( wellbeing ) OR ( psychological AND well-being ) OR ( mental AND help-seeking ) OR ( behavi*r ) ) |
| --- | --- | --- |
|  | Screening  (114 articles) | Limit to:   1. Document type (articles) – 170 2. English – 167 3. Duplicate remove intradatabase – 166 4. Duplicate remove interdatabase – 114 |
|  | Eligibility  (4 articles) | 1. Title 2. Abstract |
| **Web of Science (WOS)** | Identification  (563 article) | TS=((breast AND cancer) OR (breast AND neoplasm) OR (breast AND malignancy)) AND TS=((mobile AND health) OR (mhealth) OR (e-health) OR (ehealth) OR (telehealth) OR (telemedicine) OR (digital AND health)) AND TS=((help-seeking) OR (health AND seeking) OR (mental AND health) OR (mental AND disorder) OR (mood AND disorder) OR (mental AND hygiene) OR (well-being) OR (wellbeing) OR (psychological AND well-being) OR (mental AND help-seeking) OR (behavi*r)) |
|  | Screening  (333 articles) | Limit to:   1. Document type (articles) – 438 2. English – 429 3. Duplicate remove intradatabase – 0 4. Duplicate remove interdatabase – 333 |
|  | Eligibility  (0 articles) | 1. Title 2. Abstract |
| **Ovid Medline** | Identification  (61 article) | BREAST NEOPLASMS/ OR breast cancer.tw. OR breast malignancy.tw. AND TELEMEDICINE/ OR mobile health.tw. OR mhealth.tw. OR e-health.tw. OR ehealth.tw. OR digital health.tw. AND help-seeking.tw. OR health-seeking.tw. OR mental health.tw. OR mental disorder.tw. OR mental hygiene.tw. OR MENTAL HEALTH/ OR mood disorder.tw. OR well-being.tw. OR wellbeing.tw. OR psychological well-being.tw. OR mental help-seeking.tw. OR behaviour.tw. |
|  | Screening  (29 articles) | Limit to:   1. Document type (articles) – 59 2. English – 59 3. Duplicate remove intradatabase – 58 4. Duplicate remove interdatabase – 29 |
|  | Eligibility  (2 articles) | 1. Title 2. Abstract |
| **Reference tracking** | Eligibility  (1 articles) | 1. Title 2. Abstract |
