# Supplementary material for: Evaluating the effects of e-health interventions on mental health outcomes in individuals with breast cancer: A systematic review
Source: PLoS One. 2025 May 7;20(5):e0321495. doi: 10.1371/journal.pone.0321495 (PMC12057970; doi:10.1371/journal.pone.0321495)
Supplement: S2 Table — Comprehensive data extraction table containing study details, intervention characteristics, outcomes, effect categories, eligibility confirmation, and information about data extractors with extraction dates for each included study. (DOCX) [file pone.0321495.s002.docx]

**S2 Table including Data Extractors and Date**

| **No.** | **Author/Country (Year)** | **Population** | **Study Design** | **Intervention Description** | **Outcome Measurement (Questionnaires)** | **Result** | **Effect Category** | **Eligibility Confirmation** | | **Name of Data Extractor/Date** |
| --- | --- | --- | --- | --- | --- | --- | --- | --- | --- | --- |
| 1 | Wolff et al./Germany (2023) | • Treatment phase: In therapy/aftercare ≥12 weeks  • N = 60  • Age: 49.4 years | Design: RCT  • Duration: 12 weeks  •Assessment: Baseline, 4, 8, 12 weeks | PINK! app •Multimodal content: Nutrition, Physical activity, Mental health support  CG: Usual care | 1) PHQ-9  2) EORTC-QLQ-C30 | **Psychological Distress**  **IG** showed improvement (7.6 → 5.1)  **CG**: 6.9 → 6.2 (p < 0.01, d = 0.8)  **Fatigue**  **IG** showed improvement (51 → 41)  **CG**: 48 → 47 (p < 0.01, d = 0.2) | Significant Improvement | Yes - meets all inclusion criteria: digital intervention for breast cancer patients with psychological measurements |  | NH/AMN/22 July 2024 |
| 2 | Baik et al./USA (2020) | • Treatment phase: Within 2 years post-treatment  • N = 78  • Age: 52.5 years | • Design: Pilot RCT  • Duration: 8 weeks  •Assessment: Baseline, 6, 8 weeks | My Guide app  • Psychosocial support  • Education CG: My Health app (general health) | HRQOL  1) PHQ-9  2) BCPT  3) IES  4) CASE-cancer  5) BC knowledge | **HRQOL**  ***Physical well-being** **IG** showed worse outcomes (21.23 → 20.27)  **CG** showed no improvement (20.21 → 20.00) ***Emotional well-being**  **IG** showed worse outcomes (19.50 → 18.60)  **CG** showed slight improvement (18.89 → 19.00)  ***Functional well-being** **IG** showed worse outcomes (20.73 → 19.95)  **CG** showed slight improvement (20.42 → 21.06)  ***Social well-being IG** showed worse outcomes (21.02 → 20.90)  **CG** showed slight improvement (20.74 → 22.52)  ***BC well-being** **IG** showed worse outcomes (23.5 → 23.41)  **CG** showed slight improvement (21.37 → 22.60)  †**Symptom burden IG** showed worse outcomes (25.55 → 25.14)  **CG** showed improvement (31.32 → 27.53)  †**Cancer-specific distress** **IG** showed worse outcomes (22.50 → 23.27)  **CG** showed improvement (29.58 → 24.35)  ***Cancer-relevant self-efficacy** **IG** showed worse outcomes (44.05 → 43.05)  **CG** showed improvement (43.00 → 43.81)  ***Breast cancer knowledge** **IG** showed improvement (9.14 → 11.82)  **CG** showed slight improvement (9.21 → 9.65) | No Significant Change | Yes - meets all inclusion criteria: mobile app intervention for breast cancer survivors with psychological measurements | | NH/AMN/23 July 2024 |
| 3 | Chow et al./USA (2020) | • Treatment phase: Active treatment  • N = 40  • Age: 56.8 years | • Design: Pre-Post  • Duration: 7 weeks  •Assessment: Baseline, 7 weeks | IntelliCare app  • CBT  • Mindfulness • Positive psychology  • Support calls & SMS | 1) PHQ-4  2) PROMIS | †**General distress**  Improved from 3.96 to 2.83 (p = 0.02)  †**Anxiety symptoms**  Improved from 60.26 to 56.53 (p = 0.05)  †**Depression symptoms**  Improved from 53.77 to 51.09 (p = 0.03)  ᵇHigher scores indicate worse outcomes | Significant Improvement | Yes - meets inclusion criteria: single-arm study of digital intervention for breast cancer patients with psychological assessment | | NH/AMN/29 July 2024 |
| 4 | Villani et al./Italy (2018) | • Treatment phase: Active treatment  • N = 29  • Age: 62.76 years | • Design: RCT • Duration: 12 weeks  •Assessment: Baseline, 2, 12 weeks | SIT program  • Stress management • Relaxation  • Cognitive restructuring CG: Usual care | 1) ERQ  2) FACT-B | **ERQ** **Emotional Suppression** **IG** showed slight improvement (2.93 → 2.33)  **CG** showed worse outcomes (3.63 → 4.15) **Cognitive Reappraisal** **IG** showed slight improvement (4.99 → 5.43)  **CG** showed slight improvement (4.53 → 4.88) **FACT-B Physical well-being** **IG** showed worse outcomes (24.40 → 21.92)  **CG** showed worse outcomes (21.43 → 19.82) **Social well-being** **IG** no improvement (18.73 to 18.62)  **CG** showed slight improvement (16.21 → 17.55) **Emotional well-being** **IG** showed improvement (17.60 → 19.54)  **CG** showed slight improvement (16.43 → 16.82)  **Functional well-being** **IG** no improvement (14.13 → 14.31)  **CG** showed slight improvement (12.64 → 13.45) | No Significant Change | Yes - meets inclusion criteria: digital stress intervention program for breast cancer patients with psychological outcomes | | NH/AMN/4 August 2024 |
| 5 | Meneses et al./USA (2018) | • Treatment phase: Within 3 years post-treatment  • N = 40  • Age: 56.63 years | • Design: RCT • Duration: 24 weeks  •Assessment: Baseline, 12, 24 weeks | Support Intervention  • 3 weekly education sessions  • 6 support calls in first month  CG**:** Delayed education | 1) PCS  2) MCS  3) CES-D | ***PCS** Slight improvement from 46.02 → 47.1 (d = 0.1), but remained below population mean of 50  †**MCS** Initial improvement at T2 (48.77 → 50.29, d = 0.14), Declined slightly at T3 (49.36, d = 0.06)  †**CES-D** Slight worsening at T2 (13.68 → 14.75, d = 0.08)  Returned to baseline at T3 (13.81, d = 0.01)  All scores remained below clinical threshold (CES-D ≥ 16) | No Significant Change | Yes - meets inclusion criteria: telephone-based support intervention for breast cancer survivors with psychological outcomes | | NH/AMN/4 August 2024 |
| 6 | Kuijpers et al./The Netherlands (2016) | • Treatment phase: During or within 1 year post-treatment • N = 92  • Age: 49.5 years | • Design: Pre-Post  • Duration: 16 weeks  •Assessment: Baseline, 16 weeks | **MijnAVL Portal**  • Personalized education  • Appointment tracking  • EMR access  • Physical activity support | 1) PAM  2) SF-36  3) IPAQ | **PAM** Slight decline from 62.7 to 60.9 (not significant) **SF-36** **Self-efficacy** Increased from 65.3 to 78.5 (p = 0.021) **Mental health** Improved from 69.8 to 76.5 (p < 0.01) **Social functioning** Enhanced from 71.2 to 80.5 (p < 0.01) **IPAQ** **Vigorous activity** Significant improvement from 0 to 360 MET-min/week (p = 0.017) | Significant improvement | Yes - meets inclusion criteria: web portal intervention for breast cancer patients with psychological outcomes | | NH/AMN/30 July 2024 |
| 7 | Børøsund et al./Norway (2014) | • Treatment phase: Active treatment  • N = 176 WebChoice: 51 IPCC: 50,  Control: 53  • Age: NR | • Design: RCT • Duration: 24 weeks  •Assessment: Baseline, 8, 16, 24 weeks | **WebChoice**  • Web-based support  • Symptom monitoring  • Self-management  • Patient communication **IPCC**  • Secure messaging only CG: Usual care | 1) MSAS  2) HADS  3) CBI | **Symptom distress** **WebChoice**  Significant improvement (-0.16, p = 0.001) **IPCC** No significant change (-0.07, p = 0.21) **Anxiety** **WebChoice** Significant improvement (-0.79, p = 0.03) **IPCC** No significant change (-0.14, p = 0.72)  **Depression** **WebChoice** Significant improvement (-0.61, p = 0.03) **IPCC** Similar improvement (-0.69, p = 0.03) **Self-Efficacy** **WebChoice** Positive trend but not significant (8.81, p = 0.08)  **IPCC** No improvement (-4.89, p = 0.38) | Significant improvement in WebChoice | Yes - meets inclusion criteria: web-based intervention for breast cancer patients with psychological outcomes | | NH/AMN/7 August 2024 |

CG: Control Group; IG: Intervention Group; T1: First assessment; T2: Second assessment; T3: Third assessment; T4: Fourth assessment

Patient Health Questionnaire-9 (PHQ-9), European Organization for Research and Treatment of Cancer Quality of Life Questionnaire Core 30 (EORTC-QLQ-C30), Health-Related Quality of Life (HRQOL), Breast Cancer Prevention Trial Questionnaire (BCPT), Impact of Event Scale (IES), Communication and Attitudinal Self-Efficacy scale for cancer (CASE-cancer), Patient Health Questionnaire-4 (PHQ-4), Patient-Reported Outcomes Measurement Information System (PROMIS), Emotion Regulation Questionnaire (ERQ), Functional Assessment of Cancer Therapy-Breast (FACT-B), Physical Component Score (PCS), Mental Component Score (MCS), Center for Epidemiologic Studies Depression Scale (CES-D), Patient Activation Measure (PAM), 36-Item Short Form Health Survey (SF-36), International Physical Activity Questionnaire (IPAQ), Memorial Symptom Assessment Scale (MSAS), Hospital Anxiety and Depression Scale (HADS), Cancer Behavioral Inventory (CBI)

* Higher scores reflect better outcomes, †Higher scores reflect worse outcomes
