# Supplementary material for: Evaluating the effects of e-health interventions on mental health outcomes in individuals with breast cancer: A systematic review
Source: PLoS One. 2025 May 7;20(5):e0321495. doi: 10.1371/journal.pone.0321495 (PMC12057970; doi:10.1371/journal.pone.0321495)
Supplement: S3 Table — Detailed categorization of digital interventions by type (mobile apps, web platforms, digital delivery), organized by domains (psychological well-being, physical health, quality of life, cancer self-management), subdomains, and their corresponding outcomes. (DOCX) [file pone.0321495.s003.docx]

S3 Table. Intervention types and measure outcomes

| **Intervention Type** | **Domain** | **Subdomain** | **Outcome** |
| --- | --- | --- | --- |
| Mobile Apps | Psychological Well-Being | Distress | Significant Improvement |
| Mobile Apps | Psychological Well-Being | Distress | Significant Improvement |
| Mobile Apps | Psychological Well-Being | Distress | Negative Outcomes |
| Web Platforms | Psychological Well-Being | Distress | Significant Improvement |
| Mobile Apps | Psychological Well-Being | Depression | Significant Improvement |
| Web Platforms | Psychological Well-Being | Depression | Significant Improvement |
| Web Platforms | Psychological Well-Being | Depression | No Significant Change |
| Mobile Apps | Psychological Well-Being | Anxiety | Significant Improvement |
| Web Platforms | Psychological Well-Being | Anxiety | Significant Improvement |
| Web Platforms | Psychological Well-Being | Self-Efficacy | Significant Improvement |
| Web Platforms | Psychological Well-Being | Self-Efficacy | No Significant Change |
| Mobile Apps | Psychological Well-Being | Self-Efficacy | Negative Outcomes |
| Digital Delivery | Psychological Well-Being | Emotional Suppression | No Significant Change |
| Mobile Apps | Psychological Well-Being | Emotional Suppression | Negative Outcomes |
| Web Platforms | Psychological Well-Being | Mental Health | Significant Improvement |
| Web Platforms | Psychological Well-Being | Mental Health | No Significant Change |
| Web Platforms | Psychological Well-Being | Empowerment | Negative Outcomes |
| Digital Delivery | Psychological Well-Being | Cognitive Appraisal | No Significant Change |
| Mobile Apps | Physical Health | Fatigue | Significant Improvement |
| Mobile Apps | Physical Health | Physical Well-being | Negative Outcomes |
| Digital Delivery | Physical Health | Physical Well-being | Negative Outcomes |
| Mobile Apps | Physical Health | Symptom Burden | No Significant Change |
| Mobile Apps | QOL | Social Well-Being | Negative Outcomes |
| Digital Delivery | QOL | Social Well-Being | No Significant Change |
| Web Platforms | QOL | Social Well-Being | Significant Improvement |
| Mobile Apps | QOL | Functional Well-Being | Negative Outcomes |
| Digital Delivery | QOL | Functional Well-Being | No Significant Change |
| Mobile Apps | Cancer Self-Management | BC Well-being | Negative Outcomes |
| Mobile Apps | Cancer Self-Management | Knowledge regarding BC | Significant Improvement |
