# Supplementary material for: Evaluating the effects of e-health interventions on mental health outcomes in individuals with breast cancer: A systematic review
Source: PLoS One. 2025 May 7;20(5):e0321495. doi: 10.1371/journal.pone.0321495 (PMC12057970; doi:10.1371/journal.pone.0321495)
Supplement: S4 Table — Detailed evaluation of all included studies using the Effective Public Health Practice Project (EPHPP) quality assessment tool, showing ratings for selection bias, study design, confounders, blinding, data collection methods, withdrawals and dropouts, with component question answers and global quality ratings. (DOCX) [file pone.0321495.s004.docx]

**S4 Table: Quality Assessment Tool for Quantitative Studies**

| **Study** | **Selection Bias** | | **Study Design** | | **Confounders** | | **Blinding** | | **Data Collection Methods** | | **Withdrawals and Dropouts** | | **Global Rating** |
| --- | --- | --- | --- | --- | --- | --- | --- | --- | --- | --- | --- | --- | --- |
| Component Questions | Q1: Are the individuals selected to participate likely to be representative of the target population? | Q2: What percentage of selected individuals agreed to participate? | Q1: Study design type? | Q2: Was the study randomized? If yes, was method appropriate? | Q1: Were there important differences between groups prior to intervention? | Q2: What percentage of relevant confounders were controlled? | Q1: Were outcome assessors aware of intervention status? | Q2: Were participants aware of research question? | Q1: Were data collection tools valid? | Q2: Were data collection tools reliable? | Q1: Were withdrawals and drop-outs reported? | Q2: Percentage completing study? | Overall Quality |
| Wolff et al. (2023) | Very likely (1) | 60-79% (2) | RCT (1) | Yes, appropriate method (1) | No (1) | 80-100% (1) | Yes (2) | Yes (2) | Yes (1) | Yes (1) | Yes (1) | 80%+ (1) | Strong |
| Baik et al. (2020) | Very likely (1) | 60-79% (2) | RCT (1) | Yes, appropriate method (1) | No (1) | 80-100% (1) | Yes (2) | Yes (2) | Yes (1) | Yes (1) | Yes (1) | 80%+ (1) | Strong |
| Chow et al. (2020) | Somewhat likely (2) | <60% (3) | Cohort (2) | No (3) | Yes (2) | <60% (3) | Yes (2) | Yes (2) | Yes (1) | Yes (1) | No (3) | <60% (3) | Weak |
| Meneses et al. (2018) | Very likely (1) | 60-79% (2) | RCT (1) | Yes, appropriate method (1) | No (1) | 80-100% (1) | Yes (2) | Yes (2) | Yes (1) | Yes (1) | Yes (1) | <60% (3) | Moderate |
| Villani et al. (2018) | Very likely (1) | 60-79% (2) | RCT (1) | Yes, appropriate method (1) | No (1) | 80-100% (1) | Yes (2) | Yes (2) | Yes (1) | Yes (1) | Yes (1) | 80%+ (1) | Strong |
| Kuijpers et al. (2016) | Somewhat likely (2) | <60% (3) | Cohort (2) | No (3) | Yes (2) | <60% (3) | Yes (2) | Yes (2) | Yes (1) | Yes (1) | Yes (1) | 80%+ (1) | Weak |
| Børøsund et al. (2014) | Very likely (1) | 60-79% (2) | RCT (1) | Yes, appropriate method (1) | No (1) | 80-100% (1) | Yes (2) | Yes (2) | Yes (1) | Yes (1) | Yes (1) | 80%+ (1) | Strong |
