# Supplementary material for: Evaluating the effects of e-health interventions on mental health outcomes in individuals with breast cancer: A systematic review
Source: PLoS One. 2025 May 7;20(5):e0321495. doi: 10.1371/journal.pone.0321495 (PMC12057970; doi:10.1371/journal.pone.0321495)
Supplement: S5 Table — List of articles identified through database searches and other sources, including bibliographic information and specific reasons for exclusion where applicable. (DOCX) [file pone.0321495.s005.docx]

|  |  |  | **S5Table. Retrieved Articles and Exclusion Reasons** |  |  |  |  |  |  |  |  |
| --- | --- | --- | --- | --- | --- | --- | --- | --- | --- | --- | --- |
|  |  | **No** | **Authors** | **Years** | **Title** | **Journal** | **Reason for Exclusion** |  |  |  |  |
|  |  | 1 | Abraham, O., et al. | 2024 | Need for Culturally Competent and Responsive Cancer Education for African Immigrant Families and Youth Living in the United States | JMIR Cancer | Non Breast Cancer Population |  |  |  |  |
|  |  | 2 | Abrahams, H. J. G., et al. | 2015 | A randomized controlled trial of web-based cognitive behavioral therapy for severely fatigued breast cancer survivors (CHANGE-study): study protocol | BMC Cancer | Different Study Design |  |  |  |  |
|  |  | 3 | Abrahams, H. J. G., et al. | 2015 | A randomized controlled trial of web-based cognitive behavioral therapy for severely fatigued breast cancer survivors (CHANGE-study): study protocol | BMC Cancer | Duplicate |  |  |  |  |
|  |  | 4 | Absolom, K., et al. | 2021 | Phase III Randomized Controlled Trial of eRAPID: EHealth Intervention during Chemotherapy | Journal of Clinical Oncology | Different Study Outcome |  |  |  |  |
|  |  | 5 | Acharya, A., et al. | 2023 | An SMS and animated video intervention to increase uptake of breast cancer screening: a randomised controlled trial | The Lancet | Different Study Outcome |  |  |  |  |
|  |  | 6 | Ahmed, S., et al. | 2022 | Women's perceptions of PERSPECTIVE: a breast cancer risk stratification e-platform | HEREDITARY CANCER IN CLINICAL PRACTICE | Different Study Outcome |  |  |  |  |
|  |  | 7 | Ahmed, S., et al. | 2022 | Women's perceptions of PERSPECTIVE: a breast cancer risk stratification e-platform | HEREDITARY CANCER IN CLINICAL PRACTICE | Duplicate |  |  |  |  |
|  |  | 8 | Ainsworth, M. C., et al. | 2018 | Acceptability of a mobile phone app for measuring time use in breast cancer survivors (life in a day): Mixed-methods study | JMIR cancer | Different Study Outcome |  |  |  |  |
|  |  | 9 | Ainsworth, M. C., et al. | 2018 | Acceptability of a Mobile Phone App for Measuring Time Use in Breast Cancer Survivors (Life in a Day): Mixed-Methods Study | JMIR Cancer | Duplicate |  |  |  |  |
|  |  | 10 | Alexeeff, S. E., et al. | 2019 | Reproductive Factors and Mammographic Density: Associations Among 24,840 Women and Comparison of Studies Using Digitized Film-Screen Mammography and Full-Field Digital Mammography | AMERICAN JOURNAL OF EPIDEMIOLOGY | Different Study Outcome |  |  |  |  |
|  |  | 11 | Alexeeff, S. E., et al. | 2019 | Reproductive Factors and Mammographic Density: Associations Among 24,840 Women and Comparison of Studies Using Digitized Film-Screen Mammography and Full-Field Digital Mammography | AMERICAN JOURNAL OF EPIDEMIOLOGY | Duplicate |  |  |  |  |
|  |  | 12 | Alexy, B. B. & Elnitsky, C. | 1998 | Rural Mobile Health Unit: Outcomes | Public Health Nursing | Different Study Outcome |  |  |  |  |
|  |  | 13 | Alkan, H. & Akyildiz, D. | 2024 | Effect of monthly reminders by telephone message on women's beliefs and practice behaviours regarding breast self-examination: A randomized controlled study | INTERNATIONAL JOURNAL OF NURSING PRACTICE | Different Study Outcome |  |  |  |  |
|  |  | 14 | Allicock, M., et al. | 2021 | A Pilot and Feasibility Mobile Health Intervention to Support Healthy Behaviors in African American Breast Cancer Survivors | Journal of Racial and Ethnic Health Disparities | Different Study Outcome |  |  |  |  |
|  |  | 15 | Allicock, M., et al. | 2021 | A Pilot and Feasibility Mobile Health Intervention to Support Healthy Behaviors in African American Breast Cancer Survivors | Journal of Racial and Ethnic Health Disparities | Duplicate |  |  |  |  |
|  |  | 16 | Altmannshofer, S., Flaucher, M., Beierlein, M., et al. | 2024 | A content-based review of mobile health applications for breast cancer prevention and education: Characteristics, quality and functionality analysis | Digital Health | Non Original Articles |  |  |  |  |
|  |  | 17 | Alsuwaida, A., et al. | 2013 | Health and well-being among physicians | INTERNAL MEDICINE JOURNAL | Different Study Outcome |  |  |  |  |
|  |  | 18 | Ancona, C., Caroppo, E., De Lellis, P. | 2025 | Digital solutions supporting the quality-of-life of European cancer patients and their caregivers: a systematic literature review | Health and Technology | Non Original Articles |  |  |  |  |
|  |  | 19 | Antelo, V. S., et al. | 2022 | A Counseling Mobile App to Reduce the Psychosocial Impact of Human Papillomavirus Testing: Formative Research Using a User-Centered Design Approach in a Low-Middle-Income Setting in Argentina | JMIR Formative Research | Different Study Outcome |  |  |  |  |
|  |  | 20 | Atkins, E., et al. | 2013 | Are Obese Women More Likely to Participate in a Mobile Mammography Program? | Journal of Community Health | Different Study Outcome |  |  |  |  |
|  |  | 21 | Atkins, E., et al. | 2013 | Are Obese Women More Likely to Participate in a Mobile Mammography Program? | Journal of Community Health | Duplicate |  |  |  |  |
|  |  | 22 | Austin, J., et al. | 2023 | Compas-Y: A mixed methods pilot evaluation of a mobile self-compassion training for people with newly diagnosed cancer | Digital Health | Different Study Outcome |  |  |  |  |
|  |  | 23 | Austin, J., et al. | 2023 | Compas-Y: A mixed methods pilot evaluation of a mobile self-compassion training for people with newly diagnosed cancer | Digital Health | Duplicate |  |  |  |  |
|  |  | 24 | Aydin, A. & Gursoy, A. | 2024 | Nurse-Led Mobile App Effect on Quality of Life in Breast Cancer Patients After Surgery: Nonrandomized Controlled Prospective Cohort Study (Step 3) | CANCER NURSING | Non Original Articles |  |  |  |  |
|  |  | 25 | Baglione, A. N., et al. | 2022 | Understanding the Relationship between Mood Symptoms and Mobile App Engagement among Patients with Breast Cancer Using Machine Learning: Case Study | JMIR Medical Informatics | Different Study Design |  |  |  |  |
|  |  | 26 | Baglione, A. N., et al. | 2022 | Understanding the Relationship Between Mood Symptoms and Mobile App Engagement Among Patients With Breast Cancer Using Machine Learning: Case Study | JMIR Medical Informatics | Duplicate |  |  |  |  |
|  |  | 27 | Bahar-Ozdemir, Y., et al. | 2023 | Can assessment of disease burden and quality of life based on mobility level in patients with end-stage cancer provide an insight into unmet needs? An exploratory cross-sectional study | Physiotherapy Theory and Practice | Different Study Outcome |  |  |  |  |
|  |  | 28 | Bahar-Ozdemir, Y., et al. | 2023 | Can assessment of disease burden and quality of life based on mobility level in patients with end-stage cancer provide an insight into unmet needs? An exploratory cross-sectional study | Physiotherapy Theory and Practice | Duplicate |  |  |  |  |
|  |  | 29 | Bai, Y., et al. | 2022 | A randomized trial to promote physical activity in adult pre-hypertensive and hypertensive patients | JOURNAL OF SPORTS SCIENCES | Different Study Outcome |  |  |  |  |
|  |  | 30 | Baik, S. H., et al. | 2023 | Advancing E-health Interventions in Cancer Control and Survivorship for Hispanic/Latina Breast Cancer Patients | Springer | Different Study Outcome |  |  |  |  |
|  |  | 31 | Baik, S. H., et al. | 2023 | Advancing E-health Interventions in Cancer Control and Survivorship for Hispanic/Latina Breast Cancer Patients | Springer | Duplicate |  |  |  |  |
|  |  | 32 | Baik, S. H., et al. | 2020 | Patterns of use of smartphone-based interventions among latina breast cancer survivors: Secondary analysis of a pilot randomized controlled trial | JMIR Cancer | Included |  |  |  |  |
|  |  | 33 | Baik, S. H., et al. | 2020 | Patterns of Use of Smartphone-Based Interventions Among Latina Breast Cancer Survivors: Secondary Analysis of a Pilot Randomized Controlled Trial | JMIR Cancer | Duplicate |  |  |  |  |
|  |  | 34 | Baker, T. B., et al. | 2011 | Optimizing eHealth breast cancer interventions: Which types of eHealth services are effective? | TRANSLATIONAL BEHAVIORAL MEDICINE | Different Study Design |  |  |  |  |
|  |  | 35 | Baker, T. B., et al. | 2011 | Optimizing eHealth breast cancer interventions: which types of eHealth services are effective? | TRANSLATIONAL BEHAVIORAL MEDICINE | Duplicate |  |  |  |  |
|  |  | 36 | Balka, E., et al. | 2010 | Situating Internet Use: Information-Seeking Among Young Women with Breast Cancer | Journal of Computer-Mediated Communication | Different Study Outcome |  |  |  |  |
|  |  | 37 | Banerjee, P., et al. | 2023 | Using real-world evidence data and digital monitoring to analyze the hepatotoxic profiles of biologics across more than two million patients | Scientific Reports | Different Study Outcome |  |  |  |  |
|  |  | 38 | Banerjee, P., et al. | 2023 | Using real-world evidence data and digital monitoring to analyze the hepatotoxic profiles of biologics across more than two million patients | Scientific Reports | Duplicate |  |  |  |  |
|  |  | 39 | Baresova, Z. et al | 2023 | EHealth support for mental health of oncology patients – is there patient interest? Summary of the first year with the MOÚ MindCare mobile application | Klinicka Onkologie | Non English |  |  |  |  |
|  |  | 40 | Baseman, J., et al. | 2017 | A Mobile Breast Cancer Survivorship Care App: Pilot Study | JMIR Cancer | Different Study Outcome |  |  |  |  |
|  |  | 41 | Baseman, J., et al. | 2017 | A mobile breast cancer survivorship care app: Pilot study | JMIR Cancer | Duplicate |  |  |  |  |
|  |  | 42 | Basen-Engquist, K., et al. | 2022 | Optimization of an mHealth lifestyle intervention for families with hereditary cancer syndromes: Study protocol for a multiphase optimization strategy feasibility study | Contemporary Clinical Trials | Different Study Design |  |  |  |  |
|  |  | 43 | Basu, S., et al. | 2008 | Implications of standardized uptake value measurements of the primary lesions in proven cases of breast carcinoma with different degree of disease burden at diagnosis: Does 2-Deoxy-2-[F-18]fluoro-d-glucose-positron emission tomography predict tumor biology? | Molecular Imaging and Biology | Different Study Outcome |  |  |  |  |
|  |  | 44 | Basu, S., et al. | 2008 | Implications of standardized uptake value measurements of the primary lesions in proven cases of breast carcinoma with different degree of disease burden at diagnosis: Does 2-Deoxy-2-[F-18]fluoro-D-glucose-positron emission tomography predict tumor biology? | Molecular Imaging and Biology | Duplicate |  |  |  |  |
|  |  | 45 | Batai, K., et al. | 2022 | Formative Assessment to Improve Cancer Screenings in American Indian Men: Native Patient Navigator and mHealth Texting | International Journal of Environmental Research and Public Health | Different Study Outcome |  |  |  |  |
|  |  | 46 | Bäuerle, A., et al. | 2020 | Web-based MINDfulness and Skills-based distress reduction in cancer (MINDS): study protocol for a multicentre observational healthcare study | BMJ Open | Different Study Outcome |  |  |  |  |
|  |  | 47 | Baumgartner, M. K., et al. | 2024 | Diving into the Digital Landscape: Assessing the Quality of Online Information on Neonatal Jaundice for Parents | CHILDREN-BASEL | Different Study Outcome |  |  |  |  |
|  |  | 48 | Beacom, A. M. & Newman, S. J. | 2010 | Communicating Health Information to Disadvantaged Populations | FAMILY & COMMUNITY HEALTH | Different Study Outcome |  |  |  |  |
|  |  | 49 | Beatty, L., et al. | 2022 | Finding My Way-Advanced: can a web-based psychosocial intervention improve the mental quality of life for women with metastatic breast cancer vs attention-control? Study protocol of a randomised controlled trial | BMC Cancer | Duplicate |  |  |  |  |
|  |  | 50 | Beatty, L., et al. | 2022 | Finding My Way-Advanced: can a web-based psychosocial intervention improve the mental quality of life for women with metastatic breast cancer vs attention-control? Study protocol of a randomised controlled trial | BMC Cancer | Different Study Outcome |  |  |  |  |
|  |  | 51 | Bender, J. L., et al. | 2013 | A lot of action, but not in the right direction: Systematic review and content analysis of smartphone applications for the prevention, detection, and management of cancer | Journal of medical Internet research | Different Study Design |  |  |  |  |
|  |  | 52 | Bentley, J. R., et al. | 2022 | Feasibility and thematic analysis of narrative visualization materials with physical activity monitoring among breast cancer survivors | BMC Cancer | Different Study Design |  |  |  |  |
|  |  | 53 | Bentley, J. R., et al. | 2022 | Feasibility and thematic analysis of narrative visualization materials with physical activity monitoring among breast cancer survivors | BMC Cancer | Duplicate |  |  |  |  |
|  |  | 54 | Bhargava, S., et al. | 2019 | Gender, letters, relatives, and God: mediating actors in mammographic screening among Pakistani women in Norway | ACTA RADIOLOGICA OPEN | Different Study Outcome |  |  |  |  |
|  |  | 55 | Bisseling, E., et al. | 2019 | Development of the Therapeutic Alliance and its Association With Internet-Based Mindfulness-Based Cognitive Therapy for Distressed Cancer Patients: Secondary Analysis of a Multicenter Randomized Controlled Trial | Journal of Medical Internet Research | Different Study Design |  |  |  |  |
|  |  | 56 | Bisseling, E., et al. | 2019 | Development of the Therapeutic Alliance and its Association With Internet-Based Mindfulness-Based Cognitive Therapy for Distressed Cancer Patients: Secondary Analysis of a Multicenter Randomized Controlled Trial | Journal of Medical Internet Research | Duplicate |  |  |  |  |
|  |  | 57 | Blank, T.O., Schmidt, S.D. | 2012 | Cyber support venues for cancer | Encyclopedia of Cyber Behavior | Non Original Articles |  |  |  |  |
|  |  | 58 | Blair, C. K., et al. | 2021 | A Home-Based Mobile Health Intervention to Replace Sedentary Time With Light Physical Activity in Older Cancer Survivors: Randomized Controlled Pilot Trial | JMIR Cancer | Different Study Outcome |  |  |  |  |
|  |  | 59 | Blair, C. K., et al. | 2021 | A home-based mobile health intervention to replace sedentary time with light physical activity in older cancer survivors: Randomized controlled pilot trial | JMIR Cancer | Duplicate |  |  |  |  |
|  |  | 60 | Blair, C. K., et al. | 2021 | A Home-Based Mobile Health Intervention to Replace Sedentary Time With Light Physical Activity in Older Cancer Survivors: Randomized Controlled Pilot Trial | JMIR Cancer | Different Study Outcome |  |  |  |  |
|  |  | 61 | Blajda, J., et al. | 2022 | Application of Personalized Education in the Mobile Medical App for Breast Self-Examination | International Journal of Environmental Research and Public Health | Different Study Outcome |  |  |  |  |
|  |  | 62 | Blazey, M., et al. | 2023 | Designing a Dyad-Based Digital Health Intervention to Reduce Sedentary Time in Black Breast Cancer Survivors and Their First-degree Relatives: Human-Centered Design Study | JMIR Formative Research | Different Study Outcome |  |  |  |  |
|  |  | 63 | Blazey, M., et al. | 2023 | Designing a Dyad-Based Digital Health Intervention to Reduce Sedentary Time in Black Breast Cancer Survivors and Their First-degree Relatives: Human-Centered Design Study | JMIR Formative Research | Duplicate |  |  |  |  |
|  |  | 64 | Bleck, J., et al. | 2023 | Exploring the Link Between Demographic, Employment, and Coaching Characteristics With Financial Earning Among Health and Wellness Coaches | AMERICAN JOURNAL OF LIFESTYLE MEDICINE | Non Original Articles |  |  |  |  |
|  |  | 65 | Bloom, J. R., et al. | 2006 | Family history, perceived risk, and prostate cancer screening among African American men | CANCER EPIDEMIOLOGY BIOMARKERS & PREVENTION | Different Study Outcome |  |  |  |  |
|  |  | 66 | Borjalilu, S., et al. | 2023 | Mobile Applications to Promote Mental Health among Breast Cancer Patients: A Rapid Review | Archives of Breast Cancer | Different Study Design |  |  |  |  |
|  |  | 67 | Børøsund, E., et al. | 2014 | Comparing effects in regular practice of e-communication and Web-based self-management support among breast cancer patients: preliminary results from a randomized controlled trial | Journal of Medical Internet Research | Included |  |  |  |  |
|  |  | 68 | Børøsund, E., et al. | 2022 | Digital stress management in cancer: Testing StressProffen in a 12-month randomized controlled trial | Cancer | Non Breast Cancer Population |  |  |  |  |
|  |  | 69 | Borosund, E., et al. | 2022 | Digital stress management in cancer: Testing StressProffen in a 12-month randomized controlled trial | Cancer | Duplicate |  |  |  |  |
|  |  | 70 | Borosund, E., et al. | 2020 | Results from a randomized controlled trial testing StressProffen; an application-based stress-management intervention for cancer survivors | Cancer Medicine | Different Study Outcome |  |  |  |  |
|  |  | 71 | Børøsund, E., et al. | 2020 | Results from a randomized controlled trial testing StressProffen; an application-based stress-management intervention for cancer survivors | Cancer Medicine | Duplicate |  |  |  |  |
|  |  | 72 | Borosund, E., et al. | 2020 | Pilot testing an app-based stress management intervention for cancer survivors | Translational Behavioral Medicine | Non Original Articles |  |  |  |  |
|  |  | 73 | Børøsund, E., et al. | 2020 | Pilot testing an app-based stress management intervention for cancer survivors | Translational Behavioral Medicine | Duplicate |  |  |  |  |
|  |  | 74 | Boroumand Sani, S., et al. | 2024 | Comparison of the effectiveness of teaching "breast cancer in a simple language" via a user-friendly booklet or through WhatsApp on the learning rate and emotional status in healthy women: a randomized pre-test/post-test experimental design | International Journal of Health Promotion and Education | Different Study Outcome |  |  |  |  |
|  |  | 75 | Bowen, D. J., et al. | 2021 | Learning from and Leveraging Multi-Level Changes in Responses to the COVID 19 Pandemic to Facilitate Breast Cancer Prevention Efforts | International Journal of Environmental Research & Public Health | Non Original Articles |  |  |  |  |
|  |  | 76 | Bradbury, K., et al. | 2019 | Developing a digital intervention for cancer survivors: an evidence-, theory- and person-based approach | NPJ DIGITAL MEDICINE | Different Study Outcome |  |  |  |  |
|  |  | 77 | Bradt, J., Goodill, S.W., Dileo, C. | 2011 | Dance/movement therapy for improving psychological and physical outcomes in cancer patients | Cochrane Database of Systematic Reviews | Non Original Articles |  |  |  |  |
|  |  | 78 | Braun, A., et al. | 2018 | Tele-Motivational Interviewing for Cancer Survivors: Feasibility, Preliminary Efficacy, and Lessons Learned | JOURNAL OF NUTRITION EDUCATION AND BEHAVIOR | Different Study Outcome |  |  |  |  |
|  |  | 79 | Brown, M., et al. | 2021 | Impact of COVID-19 on an established physical activity and behaviour change support programme for cancer survivors: An exploratory survey of the Macmillan Move More service for Northern Ireland | Supportive Care in Cancer | Different Study Outcome |  |  |  |  |
|  |  | 80 | Brunyé, T. T., et al. | 2017 | Accuracy is in the eyes of the pathologist: The visual interpretive process and diagnostic accuracy with digital whole slide images | Journal of Biomedical Informatics | Different Study Outcome |  |  |  |  |
|  |  | 81 | Brunyé, T. T., et al. | 2017 | Accuracy is in the eyes of the pathologist: The visual interpretive process and diagnostic accuracy with digital whole slide images | Journal of Biomedical Informatics | Duplicate |  |  |  |  |
|  |  | 82 | Bulaj, G., et al. | 2021 | From Precision Metapharmacology to Patient Empowerment: Delivery of Self-Care Practices for Epilepsy, Pain, Depression and Cancer Using Digital Health Technologies | FRONTIERS IN PHARMACOLOGY | Non Breast Cancer Population |  |  |  |  |
|  |  | 83 | Bush, N., et al. | 2010 | Profiles of 800,000 Users of the National Cancer Institute's Cancer Information Service Since the Debut of Online Assistance, 2003-2008 | PREVENTING CHRONIC DISEASE | Different Study Outcome |  |  |  |  |
|  |  | 84 | Businelle, M. S., et al. | 2016 | Using Intensive Longitudinal Data Collected via Mobile Phone to Detect Imminent Lapse in Smokers Undergoing a Scheduled Quit Attempt | Journal of Medical Internet Research | Different Study Outcome |  |  |  |  |
|  |  | 85 | Cabling, M. L., et al. | 2022 | Revisiting agency and medical health technology: actor network theory and breast cancer survivors' perspectives on an adherence tool | Health and Technology | Different Study Outcome |  |  |  |  |
|  |  | 86 | Cabling, M. L., et al. | 2022 | Revisiting agency and medical health technology: actor network theory and breast cancer survivors' perspectives on an adherence tool | Health and Technology | Duplicate |  |  |  |  |
|  |  | 87 | Cadmus-Bertram, L., et al. | 2015 | Use of the Fitbit to Measure Adherence to a Physical Activity Intervention Among Overweight or Obese, Postmenopausal Women: Self-Monitoring Trajectory During 16 Weeks | JMIR mHealth and uHealth | Different Study Outcome |  |  |  |  |
|  |  | 88 | Cadmus-Bertram, L. A., et al. | 2015 | Randomized Trial of a Fitbit-Based Physical Activity Intervention for Women | American Journal of Preventive Medicine | Different Study Outcome |  |  |  |  |
|  |  | 89 | Cai, L., et al. | 2020 | An integrated framework for using mobile sensing to understand response to mobile interventions among breast cancer patients | Smart Health | Different Study Outcome |  |  |  |  |
|  |  | 90 | Cai, L., et al. | 2020 | An integrated framework for using mobile sensing to understand response to mobile interventions among breast cancer patients | Smart Health | Duplicate |  |  |  |  |
|  |  | 91 | Calvaresi, D., et al. | 2021 | EREBOTS: Privacy-compliant agent-based platform for multi-scenario personalized health-assistant chatbots | ELECTRONICS | Different Study Outcome |  |  |  |  |
|  |  | 92 | Calvaresi, D., et al. | 2021 | EREBOTS: Privacy-Compliant Agent-Based Platform for Multi-Scenario Personalized Health-Assistant Chatbots | ELECTRONICS | Duplicate |  |  |  |  |
|  |  | 93 | Cao, B. L., et al. | 2022 | Patient Expectation in China: Exploring Patient Satisfaction in Online and Offline Patient-Provider Communication | Frontiers in Psychology | Different Study Outcome |  |  |  |  |
|  |  | 94 | Carli, V., et al. | 2022 | The NEVERMIND e-health system in the treatment of depressive symptoms among patients with severe somatic conditions: A multicentre, pragmatic randomised controlled trial | ECLINICALMEDICINE | Non Breast Cancer Population |  |  |  |  |
|  |  | 95 | Carli, V., et al. | 2020 | A protocol for a multicentre, parallel-group, pragmatic randomised controlled trial to evaluate the NEVERMIND system in preventing and treating depression in patients with severe somatic conditions | BMC PSYCHIATRY | Non Breast Cancer Population |  |  |  |  |
|  |  | 96 | Carlson, L. E. & Garland, S. N. | 2010 | EFFECT OF PSYCHOSOCIAL INTERVENTIONS ON PSYCHOENUROENDOCRINE OUTCOMES IN CANCER PATIENTS: WHERE DO WE GO FROM HERE? | Psychoneuroendocrinology Research Trends | Non Original Articles |  |  |  |  |
|  |  | 97 | Carras, M. C., et al. | 2014 | Use of Mobile Phones, Computers and Internet Among Clients of an Inner-City Community Psychiatric Clinic | JOURNAL OF PSYCHIATRIC PRACTICE | Different Study Outcome |  |  |  |  |
|  |  | 98 | Carson, E. K., et al. | 2024 | Telehealth cognitive behaviour therapy for the management of sleep disturbance in women with early breast cancer receiving chemotherapy: a feasibility study | Supportive Care in Cancer | Different Study Outcome |  |  |  |  |
|  |  | 99 | Carson, E. K., et al. | 2024 | Telehealth cognitive behaviour therapy for the management of sleep disturbance in women with early breast cancer receiving chemotherapy: a feasibility study | Supportive Care in Cancer | Duplicate |  |  |  |  |
|  |  | 100 | Casa, C., et al. | 2023 | Integration of art and technology in personalized radiation oncology care: Experiences, evidence, and perspectives | FRONTIERS IN PUBLIC HEALTH | Different Study Outcome |  |  |  |  |
|  |  | 101 | Chae, S., et al. | 2021 | Sources of Health Information, Technology Access, and Use Among Non-English-Speaking Immigrant Women: Descriptive Correlational Study | Journal of Medical Internet Research | Different Study Outcome |  |  |  |  |
|  |  | 102 | Chae, S., et al. | 2021 | Sources of Health Information, Technology Access, and Use Among Non-English-Speaking Immigrant Women: Descriptive Correlational Study | Journal of Medical Internet Research | Duplicate |  |  |  |  |
|  |  | 103 | Chen, J., et al. | 2016 | Industry-academic partnerships: An approach to accelerate innovation | Journal of Surgical Research | Different Study Outcome |  |  |  |  |
|  |  | 104 | Chen, J., et al. | 2016 | Industry-academic partnerships: an approach to accelerate innovation | Journal of Surgical Research | Duplicate |  |  |  |  |
|  |  | 105 | Chen, Y. Y., et al. | 2018 | Effect of telehealth intervention on breast cancer patients' quality of life and psychological outcomes: A meta-analysis | Journal of Telemedicine & Telecare | Different Study Design |  |  |  |  |
|  |  | 106 | Cho, J., et al. | 2014 | A review of breast cancer survivorship issues from survivors' perspectives | Journal of Breast Cancer | Different Study Design |  |  |  |  |
|  |  | 107 | Cho, J., et al. | 2014 | A review of breast cancer survivorship issues from survivors' perspectives | Journal of Breast Cancer | Duplicate |  |  |  |  |
|  |  | 108 | Cho, Y. M., et al. | 2023 | Use of Electronic Communication with Clinicians Among Cancer Survivors: Health Information National Trend Survey in 2019 and 2020 | TELEMEDICINE AND E-HEALTH | Different Study Outcome |  |  |  |  |
|  |  | 109 | Chow, K. M., et al. | 2024 | A multimodal couple-coping intervention for enhancing sexual adjustment among breast cancer women: Study protocol for a randomised controlled trial | PLoS ONE | Different Study Outcome |  |  |  |  |
|  |  | 110 | Chow, K. M., et al. | 2024 | A multimodal couple-coping intervention for enhancing sexual adjustment among breast cancer women: Study protocol for a randomised controlled trial | PLoS ONE | Duplicate |  |  |  |  |
|  |  | 111 | Chow, K. M., et al. | 2024 | A multimodal couple-coping intervention for enhancing sexual adjustment among breast cancer women: Study protocol for a randomised controlled trial | PLoS ONE | Duplicate |  |  |  |  |
|  |  | 112 | Chow, P. I., et al. | 2020 | A Novel Mobile Phone App Intervention With Phone Coaching to Reduce Symptoms of Depression in Survivors of Women's Cancer: Pre-Post Pilot Study | JMIR Cancer | Non Breast Cancer Population |  |  |  |  |
|  |  | 113 | Chow, P. I., et al. | 2020 | Use of mental health apps by patients with breast cancer in the united states: Pilot pre-post study | JMIR Cancer | Included |  |  |  |  |
|  |  | 114 | Chow, P. I., et al. | 2020 | Use of Mental Health Apps by Patients With Breast Cancer in the United States: Pilot Pre-Post Study | JMIR Cancer | Duplicate |  |  |  |  |
|  |  | 115 | Chow, P. I., et al. | 2019 | Use of mental health apps by breast cancer patients and their caregivers in the United States: Protocol for a pilot pre-post study | JMIR Research Protocols | Different Study Outcome |  |  |  |  |
|  |  | 116 | Chow, P. I., et al. | 2019 | Use of Mental Health Apps by Breast Cancer Patients and Their Caregivers in the United States: Protocol for a Pilot Pre-Post Study | JMIR Research Protocols | Duplicate |  |  |  |  |
|  |  | 117 | Chung, I., et al. | 2020 | Exercise Promotion and Distress Reduction Using a Mobile App-Based Community in Breast Cancer Survivors | Frontiers in Oncology | Different Study Outcome |  |  |  |  |
|  |  | 118 | Chung, I. Y., et al. | 2020 | Exercise Promotion and Distress Reduction Using a Mobile App-Based Community in Breast Cancer Survivors | Frontiers in Oncology | Duplicate |  |  |  |  |
|  |  | 119 | Cieslak, K. & Golusinski, W. | 2018 | Coping with loss of ability vs. emotional control and self-esteem in women after mastectomy | REPORTS OF PRACTICAL ONCOLOGY AND RADIOTHERAPY | Different Study Outcome |  |  |  |  |
|  |  | 120 | Ciria-Suarez, L., et al. | 2022 | A Digital Cancer Ecosystem to Deliver Health and Psychosocial Education as Preventive Intervention | Cancers | Different Study Outcome |  |  |  |  |
|  |  | 121 | Ciria-Suarez, L., et al. | 2022 | A Digital Cancer Ecosystem to Deliver Health and Psychosocial Education as Preventive Intervention | Cancers | Duplicate |  |  |  |  |
|  |  | 122 | Clarke, P. M. | 2002 | Testing the convergent validity of the contingent valuation and travel cost methods in valuing the benefits of health care | HEALTH ECONOMICS | Different Study Outcome |  |  |  |  |
|  |  | 123 | Collie, K., et al. | 2006 | Distance art groups for women with breast cancer: guidelines and recommendations | Supportive Care in Cancer | Different Study Outcome |  |  |  |  |
|  |  | 124 | Collie, K., et al. | 2006 | Distance art groups for women with breast cancer: Guidelines and recommendations | Supportive Care in Cancer | Duplicate |  |  |  |  |
|  |  | 125 | Collie, K., et al. | 2006 | Distance art groups for women with breast cancer: guidelines and recommendations | Supportive Care in Cancer | Duplicate |  |  |  |  |
|  |  | 126 | Contento, I., et al. | 2022 | Developing a Diet and Physical Activity Intervention for Hispanic/Latina Breast Cancer Survivors | Cancer Control | Different Study Outcome |  |  |  |  |
|  |  | 127 | Contento, I., et al. | 2022 | Developing a Diet and Physical Activity Intervention for Hispanic/Latina Breast Cancer Survivors | Cancer Control | Duplicate |  |  |  |  |
|  |  | 128 | Conti, L., et al. | 2023 | Home-Based Treatment for Chronic Pain Combining Neuromodulation, Computer-Assisted Training, and Telemonitoring in Patients With Breast Cancer: Protocol for a Rehabilitative Study | JMIR Research Protocols | Different Study Outcome |  |  |  |  |
|  |  | 129 | Conti, L., et al. | 2023 | Home-Based Treatment for Chronic Pain Combining Neuromodulation, Computer-Assisted Training, and Telemonitoring in Patients With Breast Cancer: Protocol for a Rehabilitative Study | JMIR Research Protocols | Duplicate |  |  |  |  |
|  |  | 130 | Conti, L., et al. | 2023 | Home-Based Treatment for Chronic Pain Combining Neuromodulation, Computer-Assisted Training, and Telemonitoring in Patients With Breast Cancer: Protocol for a Rehabilitative Study | JMIR Research Protocols | Duplicate |  |  |  |  |
|  |  | 131 | Coronado, G. D., et al. | 2016 | Multilevel Intervention Raises Latina Participation in Mammography Screening: Findings from (sic)Fortaleza Latina! | CANCER EPIDEMIOLOGY BIOMARKERS & PREVENTION | Different Study Outcome |  |  |  |  |
|  |  | 132 | Couper, M. P., et al. | 2010 | Use of the Internet and Ratings of Information Sources for Medical Decisions: Results from the DECISIONS Survey | MEDICAL DECISION MAKING | Different Study Outcome |  |  |  |  |
|  |  | 133 | Crafoord, M. T., et al. | 2023 | Patients' Individualized Care Perceptions and Health Literacy Using an Interactive App During Breast and Prostate Cancer Treatment | CIN-COMPUTERS INFORMATICS NURSING | Different Study Outcome |  |  |  |  |
|  |  | 134 | Crawford, J. J., et al. | 2014 | An examination of post-traumatic growth in Canadian and American ParaSport athletes with acquired spinal cord injury | PSYCHOLOGY OF SPORT AND EXERCISE | Duplicate |  |  |  |  |
|  |  | 135 | Crawford, J. J., et al. | 2014 | An examination of post-traumatic growth in Canadian and American ParaSport athletes with acquired spinal cord injury | PSYCHOLOGY OF SPORT AND EXERCISE | Different Study Outcome |  |  |  |  |
|  |  | 136 | Crook, B., et al. | 2016 | Hanging by a thread: exploring the features of nonresponse in an online young adult cancer survivorship support community | Journal of Cancer Survivorship | Different Study Outcome |  |  |  |  |
|  |  | 137 | Crooks, C. E. & Neutens, J. J. | 1993 | Prediction and verification of a woman's intention to participate in a mammography screening program | Journal of Health Education | Different Study Outcome |  |  |  |  |
|  |  | 138 | Crooks, C. E. & Neutens, J. J. | 1993 | Prediction and verification of a woman's intention to participate in a mammography screening program | Journal of Health Education | Duplicate |  |  |  |  |
|  |  | 139 | Cruz, F.O.A.M., Vilela, R.A., Ferreira, E.B., et al. | 2019 | Evidence on the use of mobile apps during the treatment of breast cancer: Systematic review | JMIR mHealth and uHealth | Non Original Articles |  |  |  |  |
|  |  | 140 | Curran, V. R. & Church, J. G. | 1999 | A study of rural women's satisfaction with a breast cancer self-help network | Journal of Telemedicine & Telecare | Different Study Outcome |  |  |  |  |
|  |  | 141 | Curran, V. R. & Church, J. G. | 1999 | A study of rural women's satisfaction with a breast cancer self-help network | Journal of Telemedicine and Telecare | Duplicate |  |  |  |  |
|  |  | 142 | Curran, V. R. & Church, J. G. | 1999 | A study of rural women's satisfaction with a breast cancer self-help network | Journal of Telemedicine and Telecare | Duplicate |  |  |  |  |
|  |  | 143 | Da Cruz, F., et al. | 2021 | A Mobile App (AMOR Mama) for Women With Breast Cancer Undergoing Radiation Therapy: Functionality and Usability Study | Journal of Medical Internet Research | Different Study Outcome |  |  |  |  |
|  |  | 144 | Dai, Q., et al. | 2024 | Development of a supportive care framework for breast cancer survivor's unmet needs: A modified Delphi study | Journal of Clinical Nursing | Different Study Outcome |  |  |  |  |
|  |  | 145 | Dai, Q., et al. | 2024 | Development of a supportive care framework for breast cancer survivor's unmet needs: A modified Delphi study | Journal of Clinical Nursing | Duplicate |  |  |  |  |
|  |  | 146 | David, P., et al. | 2012 | A walking intervention for postmenopausal women using mobile phones and interactive voice response | Journal of Telemedicine and Telecare | Different Study Outcome |  |  |  |  |
|  |  | 147 | Davis, S. N., et al. | 2021 | Correlates of Information Seeking Behaviors and Experiences Among Adult Cancer Survivors in the USA | Journal of Cancer Education | Different Study Outcome |  |  |  |  |
|  |  | 148 | Davis, S. N., et al. | 2021 | Correlates of Information Seeking Behaviors and Experiences Among Adult Cancer Survivors in the USA | Journal of Cancer Education | Duplicate |  |  |  |  |
|  |  | 149 | Davis, S. W. & Oakley-Girvan, I. | 2015 | mHealth Education Applications Along the Cancer Continuum | Journal of Cancer Education | Different Study Outcome |  |  |  |  |
|  |  | 150 | De Almeida Marques Da Cruz, F. O., et al. | 2021 | A mobile app (AMOR Mama) for women with breast cancer undergoing radiation therapy: Functionality and usability study | Journal of Medical Internet Research | Different Study Outcome |  |  |  |  |
|  |  | 151 | De Almeida Marques Da Cruz, F. O., et al. | 2021 | A mobile app (AMOR Mama) for women with breast cancer undergoing radiation therapy: Functionality and usability study | Journal of Medical Internet Research | Duplicate |  |  |  |  |
|  |  | 152 | De Frutos, M. L., et al. | 2020 | Video conferencevsface-to-face group psychotherapy for distressed cancer survivors: Arandomized controlled trial | PSYCHO-ONCOLOGY | Non Original Articles |  |  |  |  |
|  |  | 153 | De La Torre, S., et al. | 2021 | Associations Among Wearable Activity Tracker Use, Exercise Motivation, and Physical Activity in a Cohort of Cancer Survivors: Secondary Data Analysis of the Health Information National Trends Survey | JMIR Cancer | Different Study Design |  |  |  |  |
|  |  | 154 | De Rosis, S. & Barsanti, S. | 2016 | Patient satisfaction, e-health and the evolution of the patient-general practitioner relationship: Evidence from an Italian survey | HEALTH POLICY | Different Study Outcome |  |  |  |  |
|  |  | 155 | Deetjen, U. & Powell, J. A. | 2016 | Informational and emotional elements in online support groups: a Bayesian approach to large-scale content analysis | JOURNAL OF THE AMERICAN MEDICAL INFORMATICS ASSOCIATION | Different Study Design |  |  |  |  |
|  |  | 156 | Degenhardt, T., et al. | 2023 | PRECYCLE: multicenter, randomized phase IV intergroup trial to evaluate the impact of eHealth-based patient-reported outcome (PRO) assessment on quality of life in patients with hormone receptor positive, HER2 negative locally advanced or metastatic breast cancer treated with palbociclib and an aromatase inhibitor or palbociclib and fulvestrant | Trials | Different Study Outcome |  |  |  |  |
|  |  | 157 | Degenhardt, T., et al. | 2023 | PRECYCLE: multicenter, randomized phase IV intergroup trial to evaluate the impact of eHealth-based patient-reported outcome (PRO) assessment on quality of life in patients with hormone receptor positive, HER2 negative locally advanced or metastatic breast cancer treated with palbociclib and an aromatase inhibitor or palbociclib and fulvestrant | Trials | Duplicate |  |  |  |  |
|  |  | 158 | Deguzman, P. B., et al. | 2022 | Self-reliance, Social Norms, and Self-stigma as Barriers to Psychosocial Help-Seeking Among Rural Cancer Survivors With Cancer-Related Distress: Qualitative Interview Study | JMIR Formative Research | Different Study Outcome |  |  |  |  |
|  |  | 159 | Delrieu, L., et al. | 2020 | Design and methods of a national, multicenter, randomized and controlled trial to assess the efficacy of a physical activity program to improve health-related quality of life and reduce fatigue in women with metastatic breast cancer: ABLE02 trial | BMC Cancer | Different Study Outcome |  |  |  |  |
|  |  | 160 | Delrieu, L., et al. | 2020 | Design and methods of a national, multicenter, randomized and controlled trial to assess the efficacy of a physical activity program to improve health-related quality of life and reduce fatigue in women with metastatic breast cancer: ABLE02 trial | BMC Cancer | Duplicate |  |  |  |  |
|  |  | 161 | Dewi, I., et al. | 2023 | The predictors need for complementary interventions using mobile application technology in women with breast cancer | Jurnal Ners | Different Study Outcome |  |  |  |  |
|  |  | 162 | Dey, S. | 2014 | Preventing breast cancer in LMICs via screening and/or early detection: The real and the surreal | WORLD JOURNAL OF CLINICAL ONCOLOGY | Non Original Articles |  |  |  |  |
|  |  | 163 | Di Giacomo, D., et al. | 2021 | Digital Innovation in Oncological Primary Treatment for Well-Being of Patients: Psychological Caring as Prompt for Enhancing Quality of Life | CURRENT ONCOLOGY | Different Study Outcome |  |  |  |  |
|  |  | 164 | Di Meglio, A., et al. | 2021 | Lifestyle and quality of life in patients with early-stage breast cancer receiving adjuvant endocrine therapy | Current Opinion in Oncology | Different Study Outcome |  |  |  |  |
|  |  | 165 | Duodu, N. Y., et al. | 2025 | Advancements in Telehealth: Enhancing Breast Cancer Detection and Health Automation through Smart Integration of IoT and CNN Deep Learning in Residential and Healthcare Settings | Journal of Advanced Research in Applied Sciences and Engineering Technology | Different Study Outcome |  |  |  |  |
|  |  | 166 | Eddens, K. S., et al. | 2009 | Disparities by Race and Ethnicity in Cancer Survivor Stories Available on the Web | Journal of Medical Internet Research | Different Study Outcome |  |  |  |  |
|  |  | 167 | Egbring, M., et al. | 2016 | A Mobile App to Stabilize Daily Functional Activity of Breast Cancer Patients in Collaboration With the Physician: A Randomized Controlled Clinical Trial | Journal of Medical Internet Research | Duplicate |  |  |  |  |
|  |  | 168 | Eisinger, F., et al. | 2011 | Impact of general practitioners' sex and age on systematic recommendation for cancer screening | European Journal of Cancer Prevention | Different Study Design |  |  |  |  |
|  |  | 169 | El Shafie, R. A., et al. | 2018 | Supportive Care in Radiotherapy Based on a Mobile App: Prospective Multicenter Survey | JMIR mHealth and uHealth | Different Study Outcome |  |  |  |  |
|  |  | 170 | Erashdi, M., et al. | 2023 | Libyan cancer patients at King Hussein Cancer Center for more than a decade, the current situation, and a future vision | Frontiers in Oncology | Different Study Outcome |  |  |  |  |
|  |  | 171 | Eseadi, C., Amedu, A.N. | 2024 | Examining Depression among Breast Cancer Patients in Nigeria: A Scoping Review | Archives of Breast Cancer | Non Original Articles |  |  |  |  |
|  |  | 172 | Ester, M., et al. | 2023 | Effectiveness of a Self-Monitoring App in Supporting Physical Activity Maintenance Among Rural Canadians With Cancer After an Exercise Oncology Program: Cluster Randomized Controlled Trial | JMIR Cancer | Different Study Outcome |  |  |  |  |
|  |  | 173 | Ester, M., et al. | 2023 | Effectiveness of a Self-Monitoring App in Supporting Physical Activity Maintenance Among Rural Canadians With Cancer After an Exercise Oncology Program: Cluster Randomized Controlled Trial | JMIR Cancer | Duplicate |  |  |  |  |
|  |  | 174 | Fan, Y. C., et al. | 2023 | The effectiveness of compassion-based interventions among cancer patients: A systematic review and meta-analysis | Palliative & supportive care | Different Study Design |  |  |  |  |
|  |  | 175 | Flaucher, M., Zakreuskaya, A., Nissen, M., et al. | 2023 | Evaluating the Effectiveness of Mobile Health in Breast Cancer Care: A Systematic Review | Oncologist | Non Original Articles |  |  |  |  |
|  |  | 176 | Fennell, K. M., et al. | 2017 | The consumer-driven development and acceptability testing of a website designed to connect rural cancer patients and their families, carers and health professionals with appropriate information and psychosocial support | European Journal of Cancer Care | Different Study Outcome |  |  |  |  |
|  |  | 177 | Fernandez-Alvarez, M. D., et al. | 2024 | YouTube videos on lymphedema as an information source for Spanish speaking breast cancer survivors | Supportive Care in Cancer | Different Study Outcome |  |  |  |  |
|  |  | 178 | Fernandez-Alvarez, M. D. M., et al. | 2024 | YouTube videos on lymphedema as an information source for Spanish speaking breast cancer survivors | Supportive Care in Cancer | Duplicate |  |  |  |  |
|  |  | 179 | Fielding, R. G., et al. | 2005 | Attitudes of breast cancer professionals to conventional and telemedicine-delivered multidisciplinary breast meetings | Journal of Telemedicine and Telecare | Non Original Articles |  |  |  |  |
|  |  | 180 | Fielding, R. G., et al. | 2005 | Attitudes of breast cancer professionals to conventional and telemedicine-delivered multidisciplinary breast meetings | Journal of Telemedicine and Telecare | Duplicate |  |  |  |  |
|  |  | 181 | Flynn, B. S., et al. | 1997 | Community education programs to promote mammography participation in rural New York State | Preventive Medicine | Different Study Outcome |  |  |  |  |
|  |  | 182 | Fogel, J. | 2010 | THE IMPACT OF THE INTERNET FOR THOSE WITH CANCER AND FROM RACIAL/ETHNIC GROUPS AND/OR LOW LITERACY POPULATIONS | Progress in Cancer Research | Non Original Articles |  |  |  |  |
|  |  | 183 | Fore, R., et al. | 2020 | Embedding Mobile Health Technology into the Nurses' Health Study 3 to Study Behavioral Risk Factors for Cancer | CANCER EPIDEMIOLOGY BIOMARKERS & PREVENTION | Different Study Outcome |  |  |  |  |
|  |  | 184 | Franchini, M., et al. | 2022 | Promote Community Engagement in Participatory Research for Improving Breast Cancer Prevention: The PINK Study Framework | Cancers | Different Study Outcome |  |  |  |  |
|  |  | 185 | Freer, P. E., et al. | 2015 | Breast cancer screening in the era of density notification legislation: summary of 2014 Massachusetts experience and suggestion of an evidence-based management algorithm by multi-disciplinary expert panel | Breast Cancer Research and Treatment | Different Study Outcome |  |  |  |  |
|  |  | 186 | Friedman, D. B., et al. | 2006 | Health literacy and the World Wide Web: Comparing the readability of leading incident cancers on the Internet | MEDICAL INFORMATICS AND THE INTERNET IN MEDICINE | Different Study Outcome |  |  |  |  |
|  |  | 187 | Friedman, L. C., et al. | 2005 | Breast and colorectal cancer screening among low-income women with psychiatric disorders | PSYCHO-ONCOLOGY | Different Study Outcome |  |  |  |  |
|  |  | 188 | Fritzell, K., et al. | 2023 | Making the BEST decision-the BESTa project development, implementation and evaluation of a digital Decision Aid in Swedish cancer screening programmes- a description of a research project | PLoS ONE | Different Study Outcome |  |  |  |  |
|  |  | 189 | Fritzell, K., et al. | 2023 | Making the BEST decision-The BESTa project development, implementation and evaluation of a digital Decision Aid in Swedish cancer screening programmes-a description of a research project | PLoS ONE | Duplicate |  |  |  |  |
|  |  | 190 | Fu, M. R., et al. | 2016 | A Web- and Mobile-Based Intervention for Women Treated for Breast Cancer to Manage Chronic Pain and Symptoms Related to Lymphedema: Randomized Clinical Trial Rationale and Protocol | JMIR Research Protocols | Different Study Outcome |  |  |  |  |
|  |  | 191 | Fu, M. R., et al. | 2022 | A Web- and Mobile-Based Intervention for Women Treated for Breast Cancer to Manage Chronic Pain and Symptoms Related to Lymphedema: Results of a Randomized Clinical Trial | JMIR Cancer | Different Study Outcome |  |  |  |  |
|  |  | 192 | Fung, J., et al. | 2021 | Developing a Culturally and Linguistically Targeted Breast Cancer Educational Program for a Multicultural Population | Journal of Cancer Education | Different Study Outcome |  |  |  |  |
|  |  | 193 | Galpin, A., et al. | 2017 | "Thanks for Letting Us All Share Your Mammogram Experience Virtually": Developing a Web-Based Hub for Breast Cancer Screening | JMIR Cancer | Different Study Outcome |  |  |  |  |
|  |  | 194 | Galpin, A., et al. | 2017 | "Thanks for Letting Us All Share Your Mammogram Experience Virtually": Developing a Web-Based Hub for Breast Cancer Screening | JMIR Cancer | Duplicate |  |  |  |  |
|  |  | 195 | Gao, Z., et al. | 2022 | Effects of Tai Chi App and Facebook health education programs on breast cancer survivors? stress and quality of life in the Era of pandemic | COMPLEMENTARY THERAPIES IN CLINICAL PRACTICE | Non Original Articles |  |  |  |  |
|  |  | 196 | Garcia-Alcaraz, C., et al. | 2024 | Intervention recommendations to improve uptake of breast, cervical, and colorectal cancer screening among individuals living with serious mental illness | CANCER CAUSES & CONTROL | Different Study Outcome |  |  |  |  |
|  |  | 197 | Garcia-Alcaraz, C., et al. | 2024 | Intervention recommendations to improve uptake of breast, cervical, and colorectal cancer screening among individuals living with serious mental illness | CANCER CAUSES & CONTROL | Duplicate |  |  |  |  |
|  |  | 198 | Gell, N. M., et al. | 2020 | Female cancer survivor perspectives on remote intervention components to support physical activity maintenance | Supportive Care in Cancer | Different Study Outcome |  |  |  |  |
|  |  | 199 | Geng, Z., et al. | 2024 | Effectiveness of a theory-based tailored mHealth physical activity intervention for women undergoing chemotherapy for breast cancer: A quasi-experimental study | INTERNATIONAL JOURNAL OF NURSING SCIENCES | Different Study Outcome |  |  |  |  |
|  |  | 200 | Geng, Z. H., et al. | 2024 | Effectiveness of a theory-based tailored mHealth physical activity intervention for women undergoing chemotherapy for breast cancer: A quasi-experimental study | INTERNATIONAL JOURNAL OF NURSING SCIENCES | Duplicate |  |  |  |  |
|  |  | 201 | Geng, Z. H., et al. | 2024 | Effectiveness of a theory-based tailored mHealth physical activity intervention for women undergoing chemotherapy for breast cancer: A quasi-experimental study | INTERNATIONAL JOURNAL OF NURSING SCIENCES | Duplicate |  |  |  |  |
|  |  | 202 | Gernier, F., et al. | 2023 | Impact of web application support versus standard management on adherence with adjuvant hormone therapy in patients treated for breast cancer: the WEBAPPAC study | BMC cancer | Different Study Outcome |  |  |  |  |
|  |  | 203 | Gernier, F., et al. | 2023 | Impact of web application support versus standard management on adherence with adjuvant hormone therapy in patients treated for breast cancer: the WEBAPPAC study | BMC Cancer | Duplicate |  |  |  |  |
|  |  | 204 | Ghoulami-Shilsari, F., Bandboni, M.E. | 2019 | Tele-Nursing in Chronic Disease Care: A Systematic Review | Jundishapur Journal of Chronic Disease Care | Non Original Articles |  |  |  |  |
|  |  | 205 | Gichoya, J. W., et al. | 2022 | AI recognition of patient race in medical imaging: a modelling study | The Lancet Digital | Different Study Outcome |  |  |  |  |
|  |  | 206 | Gill, C. J., et al. | 2018 | mCME project V.2.0: randomised controlled trial of a revised SMS-based continuing medical education intervention among HIV clinicians in Vietnam | BMJ GLOBAL HEALTH | Different Study Outcome |  |  |  |  |
|  |  | 207 | Gill, C. J., et al. | 2016 | The mCME Project: A Randomized Controlled Trial of an SMS-Based Continuing Medical Education Intervention for Improving Medical Knowledge among Vietnamese Community Based Physicians' Assistants | PLoS ONE | Different Study Outcome |  |  |  |  |
|  |  | 208 | Gilstad, H., et al. | 2022 | Deconstructing (e)health literacy: aspects that promote and inhibit understanding of health information in breast cancer patient pathways | International Journal of Qualitative Studies on Health and Well-being | Different Study Outcome |  |  |  |  |
|  |  | 209 | Gilstad, H., et al. | 2022 | Deconstructing (e)health literacy: aspects that promote and inhibit understanding of health information in breast cancer patient pathways | International Journal of Qualitative Studies on Health and Well-being | Duplicate |  |  |  |  |
|  |  | 210 | Ginossar, T., et al. | 2017 | Content, usability, and utilization of plain language in breast cancer mobile phone apps: A systematic analysis | JMIR mHealth and Uhealth | Different Study Design |  |  |  |  |
|  |  | 211 | Ginsburg, O. M., et al. | 2014 | An mHealth Model to Increase Clinic Attendance for Breast Symptoms in Rural Bangladesh: Can Bridging the Digital Divide Help Close the Cancer Divide? | Oncologist | Different Study Outcome |  |  |  |  |
|  |  | 212 | Ginsburg, O. M., et al. | 2014 | An mHealth model to increase clinic attendance for breast symptoms in rural Bangladesh: Can bridging the digital divide help close the cancer divide? | Oncologist | Duplicate |  |  |  |  |
|  |  | 213 | Girgis, A., et al. | 2015 | UTILISING EHEALTH TO SUPPORT SURVIVORSHIP CARE | CANCER FORUM | Different Study Outcome |  |  |  |  |
|  |  | 214 | Giunti, G., et al. | 2018 | A biopsy of Breast Cancer mobile applications: state of the practice review | International Journal of Medical Informatics | Different Study Design |  |  |  |  |
|  |  | 215 | Giunti, G., et al. | 2018 | A biopsy of Breast Cancer mobile applications: state of the practice review | International Journal of Medical Informatics | Duplicate |  |  |  |  |
|  |  | 216 | Giustiniani, A., et al. | 2023 | Use of Telemedicine to Improve Cognitive Functions and Psychological Well-Being in Patients with Breast Cancer: A Systematic Review of the Current Literature | Cancers | Non Original Articles |  |  |  |  |
|  |  | 217 | Goldstein, K. M., et al. | 2017 | Evaluation of the Department of Veterans Affairs Mental Health Services | Department of Veterans Affairs | Different Study Outcome |  |  |  |  |
|  |  | 218 | Goldstein, K. M., et al. | 2018 | Telehealth Interventions Designed for Women: an Evidence Map | JOURNAL OF GENERAL INTERNAL MEDICINE | Non Original Articles |  |  |  |  |
|  |  | 219 | Golsteijn, R. H. J., et al. | 2023 | Long-term efficacy of a computer-tailored physical activity intervention for prostate and colorectal cancer patients and survivors: A randomized controlled trial | JOURNAL OF SPORT AND HEALTH SCIENCE | Different Study Outcome |  |  |  |  |
|  |  | 220 | Gómez-Trillos, S., et al. | 2023 | Cultural adaptations to a telephone genetic counseling protocol and booklet for Latina breast cancer survivors at risk for hereditary breast and ovarian cancer | TRANSLATIONAL BEHAVIORAL MEDICINE | Different Study Outcome |  |  |  |  |
|  |  | 221 | Gómez-Trillos, S., et al. | 2023 | Cultural adaptations to a telephone genetic counseling protocol and booklet for Latina breast cancer survivors at risk for hereditary breast and ovarian cancer | TRANSLATIONAL BEHAVIORAL MEDICINE | Duplicate |  |  |  |  |
|  |  | 222 | Graetz, I., et al. | 2024 | Leveraging Mobile Health to Improve Capecitabine Adherence Among Women With Breast Cancer: A Pilot Randomized Controlled Trial | JCO Oncology Practice | Different Study Outcome |  |  |  |  |
|  |  | 223 | Graetz, I., et al. | 2024 | Leveraging Mobile Health to Improve Capecitabine Adherence among Women with Breast Cancer: A Pilot Randomized Controlled Trial | JCO Oncology Practice | Duplicate |  |  |  |  |
|  |  | 224 | Graetz, I., et al. | 2024 | Leveraging Mobile Health to Improve Capecitabine Adherence Among Women With Breast Cancer: A Pilot Randomized Controlled Trial | JCO Oncology Practice | Duplicate |  |  |  |  |
|  |  | 225 | Graf, J. et al. | 2018 | Practicability and Efficiency of E-health Applications in Patient-Reported Outcomes: State of and Need for Research | GESUNDHEITSWESE | Non English |  |  |  |  |
|  |  | 226 | Grau, I. et all | 2013 | Forumclinic: the shaping of virtual communities to assist patients with chronic diseases | Studies in Health Technology and Informatics | Non Original Articles |  |  |  |  |
|  |  | 227 | Greer, J. A., et al. | 2019 | Randomized trial of a tailored cognitive‐behavioral therapy mobile application for anxiety in patients with incurable cancer | The oncologist | Different Study Outcome |  |  |  |  |
|  |  | 228 | Gregory, M. E., et al. | 2024 | Effectiveness of mobile health for exercise promotion on cardiorespiratory fitness after a cancer diagnosis: A systematic review and meta-analysis | Cancer Medicine | Different Study Design |  |  |  |  |
|  |  | 229 | Greidanus, M. A., et al. | 2020 | Supporting employers to enhance the return to work of cancer survivors: development of a web-based intervention (MiLES intervention) | Journal of Cancer Survivorship | Different Study Outcome |  |  |  |  |
|  |  | 230 | Greidanus, M. A., et al. | 2020 | Supporting employers to enhance the return to work of cancer survivors: development of a web-based intervention (MiLES intervention) | Journal of Cancer Survivorship | Duplicate |  |  |  |  |
|  |  | 231 | Grimsbo, G. H., et al. | 2011 | Left Hanging in the Air Experiences of Living With Cancer as Expressed Through E-mail Communications With Oncology Nurses | CANCER NURSING | Different Study Outcome |  |  |  |  |
|  |  | 232 | Gupta, A., et al. | 2020 | Recruiting breast cancer patients for mHealth research: Obstacles to clinic-based recruitment for a mobile phone app intervention study | Clinical Trials | Different Study Outcome |  |  |  |  |
|  |  | 233 | Gupta, A., et al. | 2020 | Recruiting breast cancer patients for mHealth research: Obstacles to clinic-based recruitment for a mobile phone app intervention study | Clinical Trials | Duplicate |  |  |  |  |
|  |  | 234 | Gupta, A., et al. | 2020 | Recruiting breast cancer patients for mHealth research: Obstacles to clinic-based recruitment for a mobile phone app intervention study | Clinical Trials | Duplicate |  |  |  |  |
|  |  | 235 | Gurrieri, L. & Drenten, J. | 2019 | Visual storytelling and vulnerable health care consumers: normalising practices and social support through Instagram | Journal of Services Marketing | Different Study Outcome |  |  |  |  |
|  |  | 236 | Gurrieri, L. & Drenten, J. | 2019 | Visual storytelling and vulnerable health care consumers: normalising practices and social support through Instagram | Journal of Services Marketing | Duplicate |  |  |  |  |
|  |  | 237 | Gustafson, D. H., et al. | 2001 | Effect of computer support on younger women with breast cancer | JOURNAL OF GENERAL INTERNAL MEDICINE | Different Study Outcome |  |  |  |  |
|  |  | 238 | Gustafson, D. H., et al. | 2021 | A Web-Based eHealth Intervention to Improve the Quality of Life of Older Adults With Multiple Chronic Conditions: Protocol for a Randomized Controlled Trial | JMIR Research Protocols | Different Study Outcome |  |  |  |  |
|  |  | 239 | Gyawali, B., et al. | 2023 | A systematic review of eHealth technologies for breast cancer supportive care | Cancer Treatment Reviews | Non Original Articles |  |  |  |  |
|  |  | 240 | Gyawali, B., et al. | 2023 | A systematic review of eHealth technologies for breast cancer supportive care | Cancer Treatment Reviews | Duplicate |  |  |  |  |
|  |  | 241 | Hafslund, B., et al. | 2012 | Effects of False-Positive Results in a Breast Screening Program on Anxiety, Depression and Health-Related Quality of Life | CANCER NURSING | Different Study Outcome |  |  |  |  |
|  |  | 242 | Haghani, M., Abbasi, S., Abdoli, L., et al. | 2024 | Blue Light and Digital Screens Revisited: A New Look at Blue Light from the Vision Quality, Circadian Rhythm and Cognitive Functions Perspective | Journal of Biomedical Physics and Engineering | Non Original Articles |  |  |  |  |
|  |  | 243 | Hall, D. L., et al. | 2022 | A spotlight on avoidance coping to manage fear of recurrence among breast cancer survivors in an eHealth intervention | Journal of Behavioral Medicine | Different Study Outcome |  |  |  |  |
|  |  | 244 | Hall, D. L., et al. | 2022 | A spotlight on avoidance coping to manage fear of recurrence among breast cancer survivors in an eHealth intervention | Journal of Behavioral Medicine | Duplicate |  |  |  |  |
|  |  | 245 | Hall, D. L., et al. | 2022 | A spotlight on avoidance coping to manage fear of recurrence among breast cancer survivors in an eHealth intervention | Journal of Behavioral Medicine | Duplicate |  |  |  |  |
|  |  | 246 | Han, J. Y., et al. | 2017 | How Cancer Patients Use and Benefit from an Interactive Cancer Communication System | JOURNAL OF HEALTH COMMUNICATION | Different Study Outcome |  |  |  |  |
|  |  | 247 | Han, J. Y., et al. | 2010 | Factors Associated with Use of Interactive Cancer Communication System: An Application of the Comprehensive Model of Information Seeking | Journal of Computer-Mediated Communication | Different Study Outcome |  |  |  |  |
|  |  | 248 | Han, J. Y., et al. | 2010 | Factors Associated with Use of Interactive Cancer Communication System: An Application of the Comprehensive Model of Information Seeking | Journal of Computer-Mediated Communication | Duplicate |  |  |  |  |
|  |  | 249 | Harder, H., et al. | 2017 | A user-centred approach to developing bWell, a mobile app for arm and shoulder exercises after breast cancer treatment | Journal of Cancer Survivorship | Different Study Outcome |  |  |  |  |
|  |  | 250 | Harder, H., et al. | 2017 | A user-centred approach to developing bWell, a mobile app for arm and shoulder exercises after breast cancer treatment | Journal of Cancer Survivorship | Duplicate |  |  |  |  |
|  |  | 251 | Heinrich, R., et al. | 2024 | Effects of Mobile Application-Based Cognitive Behavioral Therapy on Psychological Outcomes in Women Treated for Breast Cancer: A Randomized Controlled Pilot Trial in Germany | PSYCHO-ONCOLOGY | Different Study Outcome |  |  |  |  |
|  |  | 252 | Heinrich, R., et al. | 2024 | Effects of Mobile Application-Based Cognitive Behavioral Therapy on Psychological Outcomes in Women Treated for Breast Cancer: A Randomized Controlled Pilot Trial in Germany | PSYCHO-ONCOLOGY | Duplicate |  |  |  |  |
|  |  | 253 | Hernandez, M. Y. & Organista, K. C. | 2013 | Entertainment-Education? A Fotonovela? A New Strategy to Improve Depression Literacy and Help-Seeking Behaviors in At-Risk Immigrant Latinas | AMERICAN JOURNAL OF COMMUNITY PSYCHOLOGY | Different Study Outcome |  |  |  |  |
|  |  | 254 | Holt, C. L., et al. | 2019 | Web-based versus in-person methods for training lay community health advisors to implement health promotion workshops: Participant outcomes from a cluster-randomized trial | TRANSLATIONAL BEHAVIORAL MEDICINE | Different Study Outcome |  |  |  |  |
|  |  | 255 | Holt, C. L., et al. | 2019 | Web-based versus in-person methods for training lay community health advisors to implement health promotion workshops: participant outcomes from a cluster-randomized trial | TRANSLATIONAL BEHAVIORAL MEDICINE | Duplicate |  |  |  |  |
|  |  | 256 | Holt, C. L., et al. | 2009 | Religious Involvement and Prostate Cancer Screening Behaviors Among Southeastern African American Men | AMERICAN JOURNAL OF MENS HEALTH | Different Study Outcome |  |  |  |  |
|  |  | 257 | Horesh, D., et al. | 2022 | Virtual Reality Combined with Artificial Intelligence (VR-AI) Reduces Hot Flashes and Improves Psychological Well-Being in Women with Breast and Ovarian Cancer: A Pilot Study | HEALTHCARE | Different Study Outcome |  |  |  |  |
|  |  | 258 | Horesh, D., et al. | 2022 | Virtual Reality Combined with Artificial Intelligence (VR-AI) Reduces Hot Flashes and Improves Psychological Well-Being in Women with Breast and Ovarian Cancer: A Pilot Study | HEALTHCARE | Duplicate |  |  |  |  |
|  |  | 259 | Horn, A., et al. | 2023 | Systematic review on the effectiveness of mobile health applications on mental health of breast cancer survivors | Journal of Cancer Survivorship | Non Original Articles |  |  |  |  |
|  |  | 260 | Houghton, L.C., Howland, R.E., McDonald, J.A. | 2019 | Mobilizing Breast Cancer Prevention Research Through Smartphone Apps: A Systematic Review of the Literature | Frontiers in Public Health | Non Original Articles |  |  |  |  |
|  |  | 261 | Hu, X. Y., et al. | 2012 | The Prepared Patient: Information Seeking of Online Support Group Members Before Their Medical Appointments | JOURNAL OF HEALTH COMMUNICATION | Different Study Outcome |  |  |  |  |
|  |  | 262 | Huang, C. H., et al. | 2021 | Health literacy and cancer screening behaviors among community-dwelling female adults in Taiwan | WOMEN & HEALTH | Different Study Outcome |  |  |  |  |
|  |  | 263 | Huang, C. H., et al. | 2021 | Health literacy and cancer screening behaviors among community-dwelling female adults in Taiwan | WOMEN & HEALTH | Duplicate |  |  |  |  |
|  |  | 264 | Huberty, J., et al. | 2020 | Cancer Patients' and Survivors' Perceptions of the Calm App: Cross-Sectional Descriptive Study | JMIR Cancer | Different Study Outcome |  |  |  |  |
|  |  | 265 | Innominato, P., et al. | 2018 | Home-Based e-Health Platform for Multidimensional Telemonitoring of Symptoms, Body Weight,Sleep, and Circadian Activity: Relevance for Chronomodulated Administration of Irinotecan, Fluorouracil-Leucovorin and Oxaliplatin at Home-Results From a Pilot Study | JCO CLINICAL CANCER INFORMATICS | Different Study Outcome |  |  |  |  |
|  |  | 266 | Inupakutika, D., et al. | 2022 | On the Performance of Cloud-Based mHealth Applications: A Methodology on Measuring Service Response Time and a Case Study | IEEE ACCESS | Different Study Outcome |  |  |  |  |
|  |  | 267 | Inupakutika, D., et al. | 2022 | On the Performance of Cloud-Based mHealth Applications: A Methodology on Measuring Service Response Time and a Case Study | IEEE ACCESS | Duplicate |  |  |  |  |
|  |  | 268 | Ireland, A. V., et al. | 2019 | Walking groups for women with breast cancer: Mobilising therapeutic assemblages of walk, talk and place | SOCIAL SCIENCE & MEDICINE | Different Study Outcome |  |  |  |  |
|  |  | 269 | Isakoff, S. J., et al. | 2023 | Feasibility of introducing a smartphone navigation application into the care of breast cancer patients (The FIONA Study) | Breast Cancer Research and Treatment | Different Study Outcome |  |  |  |  |
|  |  | 270 | Jabeen, Z., et al. | 2021 | Effect of health education on awareness and practices of breast self-examination among females attending a charitable hospital at North Karachi | JOURNAL OF THE PAKISTAN MEDICAL ASSOCIATION | Different Study Outcome |  |  |  |  |
|  |  | 271 | Jacobs, M., et al. | 2019 | Usability Evaluation of an Adaptive Information Recommendation System for Breast Cancer Patients | AMIA Annual Symposium Proceedings | Different Study Outcome |  |  |  |  |
|  |  | 272 | Jacobs, M., et al. | 2018 | MyPath: Investigating breast cancer patients' use of personalized health information | Proceedings of the ACM on Human-Computer Interaction | Different Study Outcome |  |  |  |  |
|  |  | 273 | Jansen, H., et al. | 2024 | Optimization of clinical care through digital applications exemplified by breast cancer | Gynakologie | Non English |  |  |  |  |
|  |  | 274 | Janssen, A., et al. | 2016 | An Online Learning Module to Increase Self-Efficacy and Involvement in Care for Patients With Advanced Lung Cancer: Research Protocol | JMIR Research Protocols | Different Study Outcome |  |  |  |  |
|  |  | 275 | Jatho, A., et al. | 2020 | Socio-culturally mediated factors and lower level of education are the main influencers of functional cervical cancer literacy among women in Mayuge, Eastern Uganda | ECANCERMEDICALSCIENCE | Different Study Outcome |  |  |  |  |
|  |  | 276 | Jensen, R. E., et al. | 2024 | National Cancer Institute-funded grants focused on synchronous telehealth cancer care delivery: a portfolio analysis | JNCI Monographs | Non Original Articles |  |  |  |  |
|  |  | 277 | Jiang, L., et al. | 2024 | Effects of the "AI-TA" Mobile App With Intelligent Design on Psychological and Related Symptoms of Young Survivors of Breast Cancer: Randomized Controlled Trial | JMIR mHealth and uHealth | Different Study Outcome |  |  |  |  |
|  |  | 278 | Jiang, L. L., et al. | 2024 | Effects of the "AI-TA" Mobile App With Intelligent Design on Psychological and Related Symptoms of Young Survivors of Breast Cancer: Randomized Controlled Trial | JMIR mHealth and uHealth | Duplicate |  |  |  |  |
|  |  | 279 | Jones, J., et al. | 2018 | Novel Approach to Cluster Patient-Generated Data Into Actionable Topics: Case Study of a Web-Based Breast Cancer Forum | JMIR Medical Informatics | Different Study Outcome |  |  |  |  |
|  |  | 280 | Jones, J., et al. | 2018 | Novel Approach to Cluster Patient-Generated Data Into Actionable Topics: Case Study of a Web-Based Breast Cancer Forum | JMIR Medical Informatics | Duplicate |  |  |  |  |
|  |  | 281 | Jones, J., et al. | 2018 | Novel Approach to Cluster Patient-Generated Data Into Actionable Topics: Case Study of a Web-Based Breast Cancer Forum | JMIR Medical Informatics | Duplicate |  |  |  |  |
|  |  | 282 | Jones, J., Pradhan, M., Hosseini, M., et al. | 2018 | Novel approach to cluster patient-generated data into actionable topics: Case study of a web-based breast cancer forum | JMIR Medical Informatics | Non Original Articles |  |  |  |  |
|  |  | 283 | Juarez-Reyes, M., et al. | 2024 | A Randomized Controlled Trial of a Culturally Adapted, Community-Based, Remotely Delivered Mindfulness Program for Latinx Patients With Breast Cancer is Acceptable and Feasible While Reducing Anxiety | GLOBAL ADVANCES IN INTEGRATIVE MEDICINE AND HEALTH | Different Study Outcome |  |  |  |  |
|  |  | 284 | Jung, M., et al. | 2023 | The Impact of a Mobile Support Group on Distress and Physical Activity in Breast Cancer Survivors: Randomized, Parallel-Group, Open-Label, Controlled Trial | Journal of Medical Internet Research | Different Study Outcome |  |  |  |  |
|  |  | 285 | Jung, M., et al. | 2023 | The Impact of a Mobile Support Group on Distress and Physical Activity in Breast Cancer Survivors: Randomized, Parallel-Group, Open-Label, Controlled Trial | Journal of Medical Internet Research | Duplicate |  |  |  |  |
|  |  | 286 | Jung, M. Y., et al. | 2023 | The Impact of a Mobile Support Group on Distress and Physical Activity in Breast Cancer Survivors: Randomized, Parallel-Group, Open-Label, Controlled Trial | Journal of Medical Internet Research | Duplicate |  |  |  |  |
|  |  | 287 | Kalke, K., et al. | 2020 | Use of evidence-based best practices and behavior change techniques in breast cancer apps: Systematic analysis | JMIR mHealth and uHealth | Different Study Design |  |  |  |  |
|  |  | 288 | Kalke, K., et al. | 2020 | Use of Evidence-Based Best Practices and Behavior Change Techniques in Breast Cancer Apps: Systematic Analysis | JMIR mHealth and uHealth | Duplicate |  |  |  |  |
|  |  | 289 | Kalra, M., et al. | 2022 | Making National Cancer Institute-Designated Comprehensive Cancer Center Knowledge Accessible to Community Oncologists via an Online Tumor Board: Longitudinal Observational Study | JMIR Cancer | Different Study Outcome |  |  |  |  |
|  |  | 290 | Kanera, I. M., et al. | 2016 | Lifestyle-related effects of the web-based Kanker Nazorg Wijzer (Cancer Aftercare Guide) intervention for cancer survivors: a randomized controlled trial | Journal of Cancer Survivorship | Different Study Outcome |  |  |  |  |
|  |  | 291 | Kanera, I. M., et al. | 2017 | Long-term effects of a web-based cancer aftercare intervention on moderate physical activity and vegetable consumption among early cancer survivors: a randomized controlled trial | INTERNATIONAL JOURNAL OF BEHAVIORAL NUTRITION AND PHYSICAL ACTIVITY | Different Study Outcome |  |  |  |  |
|  |  | 292 | Kanera, I. M., et al. | 2017 | Long-term effects of a web-based cancer aftercare intervention on moderate physical activity and vegetable consumption among early cancer survivors: a randomized controlled trial | INTERNATIONAL JOURNAL OF BEHAVIORAL NUTRITION AND PHYSICAL ACTIVITY | Duplicate |  |  |  |  |
|  |  | 293 | Karim, H., et al. | 2020 | Mobile health applications for improving the sexual health outcomes among adults with chronic diseases: A systematic review | Digital Health | Non Original Articles |  |  |  |  |
|  |  | 294 | Keikha, L., et al. | 2022 | Telerehabilitation and monitoring physical activity in patient with breast cancer: Systematic review | IRANIAN JOURNAL OF NURSING AND MIDWIFERY RESEARCH | Non Original Articles |  |  |  |  |
|  |  | 295 | Kemp, E., et al. | 2018 | Online information and support needs of women with advanced breast cancer: a qualitative analysis | Supportive Care in Cancer | Different Study Design |  |  |  |  |
|  |  | 296 | Kendzor, D. E., et al. | 2016 | Impact of a mobile phone intervention to reduce sedentary behavior in a community sample of adults: A quasi-experimental evaluation | Journal of Medical Internet Research | Different Study Outcome |  |  |  |  |
|  |  | 297 | Kendzor, D. E., et al. | 2016 | Impact of a Mobile Phone Intervention to Reduce Sedentary Behavior in a Community Sample of Adults: A Quasi-Experimental Evaluation | Journal of Medical Internet Research | Duplicate |  |  |  |  |
|  |  | 298 | Khalil, G. E. | 2012 | When Losing Means Winning: The Impact of Conflict in a Digital Game on Young Adults' Intentions to Get Protected from Cancer | GAMES FOR HEALTH JOURNAL | Different Study Outcome |  |  |  |  |
|  |  | 299 | Khondakar, K. R., et al. | 2023 | Perspective of point-of-care sensing systems in cancer management | MATERIALS ADVANCES | Different Study Outcome |  |  |  |  |
|  |  | 300 | Kim, H., Mahmood, A. & Powell, M. P. | 2021 | Health Information-seeking through Personal Health Records among Women Susceptible to Breast Cancer | HEALTH BEHAVIOR AND POLICY REVIEW | Different Study Design |  |  |  |  |
|  |  | 301 | Kim, J., et al. | 2016 | Depression screening using daily mental-health ratings from a smartphone application for breast cancer patients | Journal of Medical Internet Research | Different Study Outcome |  |  |  |  |
|  |  | 302 | Kim, J., et al. | 2016 | Depression Screening Using Daily Mental-Health Ratings from a Smartphone Application for Breast Cancer Patients | Journal of Medical Internet Research | Duplicate |  |  |  |  |
|  |  | 303 | Kim, J., et al. | 2016 | Depression Screening Using Daily Mental-Health Ratings from a Smartphone Application for Breast Cancer Patients | Journal of Medical Internet Research | Duplicate |  |  |  |  |
|  |  | 304 | Kim, S. C., et al. | 2013 | Predictors of Online Health Information Seeking Among Women with Breast Cancer: The Role of Social Support Perception and Emotional Well-Being | Journal of Computer-Mediated Communication | Different Study Outcome |  |  |  |  |
|  |  | 305 | Kim, S. C., et al. | 2019 | Interactivity, Presence, and Targeted Patient Care: Mapping e-Health Intervention Effects Over Time for Cancer Patients with Depression | Health Communication | Different Study Outcome |  |  |  |  |
|  |  | 306 | Kim, S. C., et al. | 2019 | Interactivity, Presence, and Targeted Patient Care: Mapping e-Health Intervention Effects Over Time for Cancer Patients with Depression | Health Communication | Duplicate |  |  |  |  |
|  |  | 307 | Kim, S. C., et al. | 2019 | Interactivity, Presence, and Targeted Patient Care: Mapping e-Health Intervention Effects Over Time for Cancer Patients with Depression | Health Communication | Duplicate |  |  |  |  |
|  |  | 308 | Kim, Y. M. | 2015 | Is seeking health information online different from seeking general information online? | JOURNAL OF INFORMATION SCIENCE | Different Study Outcome |  |  |  |  |
|  |  | 309 | Kinner, E. M., et al. | 2018 | Internet-Based Group Intervention for Ovarian Cancer Survivors: Feasibility and Preliminary Results | JMIR Cancer | Different Study Outcome |  |  |  |  |
|  |  | 310 | Kinner, E. M., et al. | 2018 | Internet-Based Group Intervention for Ovarian Cancer Survivors: Feasibility and Preliminary Results | JMIR Cancer | Duplicate |  |  |  |  |
|  |  | 311 | Kirkovits, T., et al. | 2016 | ehealth in modern patient-caregiver communication: High rate of acceptance among physicians for additional support of breast cancer patients during long-term therapy | JMIR Cancer | Different Study Outcome |  |  |  |  |
|  |  | 312 | Kirkovits, T., et al. | 2016 | eHealth in Modern Patient-Caregiver Communication: High Rate of Acceptance Among Physicians for Additional Support of Breast Cancer Patients During Long-Term Therapy | JMIR Cancer | Duplicate |  |  |  |  |
|  |  | 313 | Kithulegoda, N., et al. | 2024 | Assessing the effectiveness of "BETTER Women", a community-based, primary care-linked peer health coaching programme for chronic disease prevention: protocol for a pragmatic, wait-list controlled, type 1 hybrid effectiveness-implementation trial | BMJ Open | Different Study Outcome |  |  |  |  |
|  |  | 314 | Kithulegoda, N., et al. | 2024 | Assessing the effectiveness of "BETTER Women", a community-based, primary care-linked peer health coaching programme for chronic disease prevention: protocol for a pragmatic, wait-list controlled, type 1 hybrid effectiveness-implementation trial | BMJ Open | Duplicate |  |  |  |  |
|  |  | 315 | Kithulegoda, N., et al. | 2024 | Assessing the effectiveness of "BETTER Women", a community-based, primary care-linked peer health coaching programme for chronic disease prevention: protocol for a pragmatic, wait-list controlled, type 1 hybrid effectiveness-implementation trial | BMJ Open | Duplicate |  |  |  |  |
|  |  | 316 | Kissin, M.W., Subramanian, A. | 2023 | To Follow-Up or Not to Follow-Up, That Is the Question | Oncoplastic Breast Surgery: A Practical Guide | Non Original Articles |  |  |  |  |
|  |  | 317 | Koc, Z., Kaplan, E. & Tanriverdi, D. | 2024 | The effectiveness of telehealth programs on the mental health of women with breast cancer: A systematic review | Journal of Telemedicine & Telecare | Different Study Design |  |  |  |  |
|  |  | 318 | Koç, Z., Kaplan, E. & Tanrıverdi, D. | 2024 | The effectiveness of telehealth programs on the mental health of women with breast cancer: A systematic review | Journal of Telemedicine & Telecare | Duplicate |  |  |  |  |
|  |  | 319 | Kowalski, C., et al. | 2014 | Changes over time in the utilization of disease-related internet information in newly diagnosed breast cancer patients 2007 to 2013 | Journal of Medical Internet Research | Different Study Outcome |  |  |  |  |
|  |  | 320 | Kowalski, C., et al. | 2014 | Changes Over Time in the Utilization of Disease-Related Internet Information in Newly Diagnosed Breast Cancer Patients 2007 to 2013 | Journal of Medical Internet Research | Different Study Outcome |  |  |  |  |
|  |  | 321 | Kratzke, C. & Wilson, S. | 2014 | Ethnic differences in breast cancer prevention information-seeking among rural women: Will provider mobile messages work? | Journal of Cancer Education | Different Study Outcome |  |  |  |  |
|  |  | 322 | Kratzke, C. & Wilson, S. | 2014 | Ethnic Differences in Breast Cancer Prevention Information-Seeking Among Rural Women: Will Provider Mobile Messages Work? | Journal of Cancer Education | Duplicate |  |  |  |  |
|  |  | 323 | Kratzke, C., Wilson, S. & Vilchis, H. | 2013 | Reaching rural women: Breast cancer prevention information seeking behaviors and interest in internet, cell phone, and text use | Journal of Community Health | Different Study Outcome |  |  |  |  |
|  |  | 324 | Kratzke, C., Wilson, S. & Vilchis, H. | 2013 | Reaching Rural Women: Breast Cancer Prevention Information Seeking Behaviors and Interest in Internet, Cell Phone, and Text Use | Journal of Community Health | Duplicate |  |  |  |  |
|  |  | 325 | Krause, N., et al. | 2022 | 'That would have been the perfect thing after diagnosis': development of a digital lifestyle management application in multiple sclerosis | Therapeutic Advances in Neurological Disorders | Different Study Outcome |  |  |  |  |
|  |  | 326 | Krause, N., et al. | 2022 | 'That would have been the perfect thing after diagnosis': development of a digital lifestyle management application in multiple sclerosis | JMIR Cancer | Duplicate |  |  |  |  |
|  |  | 327 | Krebs, P., et al. | 2017 | An ehealth intervention to increase physical activity and healthy eating in older adult cancer survivors: Summative evaluation results | JMIR Cancer | Different Study Outcome |  |  |  |  |
|  |  | 328 | Krebs, P., et al. | 2017 | An eHealth Intervention to Increase Physical Activity and Healthy Eating in Older Adult Cancer Survivors: Summative Evaluation Results | JMIR Cancer | Duplicate |  |  |  |  |
|  |  | 329 | Krok-Schoen, J. L., et al. | 2019 | Increasing Adherence to Adjuvant Hormone Therapy Among Patients With Breast Cancer: A Smart Phone App-Based Pilot Study | Cancer Control | Different Study Outcome |  |  |  |  |
|  |  | 330 | Krok-Schoen, J. L., et al. | 2024 | Social determinants of health and depressive symptoms before and after cancer diagnosis | JOURNAL OF WOMEN & AGING | Different Study Outcome |  |  |  |  |
|  |  | 331 | Krok-Schoen, J. L., et al. | 2024 | Social determinants of health and depressive symptoms before and after cancer diagnosis | JOURNAL OF WOMEN & AGING | Duplicate |  |  |  |  |
|  |  | 332 | Krusche, A., et al. | 2019 | Renewed: Protocol for a randomised controlled trial of a digital intervention to support quality of life in cancer survivors | BMJ open | Different Study Outcome |  |  |  |  |
|  |  | 333 | Kruse, C. S., Pacheco, G. J., Vargas, B., Lozano, N., Castro, S. & Gattu, M. | 2022 | Leveraging Telehealth for the Management of Breast Cancer: A Systematic Review | HEALTHCARE | Non Original Articles |  |  |  |  |
|  |  | 334 | Kubo, A., et al. | 2019 | A Randomized Controlled Trial of mHealth Mindfulness Intervention for Cancer Patients and Informal Cancer Caregivers: A Feasibility Study Within an Integrated Health Care Delivery System | Integrative Cancer Therapies | Different Study Outcome |  |  |  |  |
|  |  | 335 | Kubo, A., et al. | 2019 | A Randomized Controlled Trial of mHealth Mindfulness Intervention for Cancer Patients and Informal Cancer Caregivers: A Feasibility Study Within an Integrated Health Care Delivery System | Integrative Cancer Therapies | Duplicate |  |  |  |  |
|  |  | 336 | Kudadjie-Gyamfi, E., Consedine, N. S., Ungar, T. & Magai, C. | 2008 | Influence of coping with prostate cancer threat on frequency of digital rectal examinations | AMERICAN JOURNAL OF HEALTH BEHAVIOR | Different Study Outcome |  |  |  |  |
|  |  | 337 | Kuijpers, W., et al. | 2016 | eHealth for Breast Cancer Survivors: Use, Feasibility and Impact of an Interactive Portal | JMIR Cancer | Included |  |  |  |  |
|  |  | 338 | Kuijpers, W., et al. | 2016 | ehealth for breast cancer survivors: Use, feasibility and impact of an interactive portal | JMIR Cancer | Duplicate |  |  |  |  |
|  |  | 339 | Kunin, A., et al. | 2020 | Voice perturbations under the stress overload in young individuals: phenotyping and suboptimal health as predictors for cascading pathologies | EPMA JOURNAL | Different Study Outcome |  |  |  |  |
|  |  | 340 | Kunkler, I., et al. | 2006 | Group dynamics in telemedicine-delivered and standard multidisciplinary team meetings: Results from the TELEMAM randomised trial | Journal of Telemedicine and Telecare | Non Original Articles |  |  |  |  |
|  |  | 341 | Kunkler, I., et al. | 2006 | Group dynamics in telemedicine-delivered and standard multidisciplinary team meetings: results from the TELEMAM randomised trial | Journal of Telemedicine and Telecare | Duplicate |  |  |  |  |
|  |  | 342 | Labrague, L. J., et al. | 2021 | Effects of mobile text messaging on breast cancer and breast self-examination (BSE) knowledge, BSE self-efficacy, and BSE frequency: a randomised controlled trial | SCANDINAVIAN JOURNAL OF CARING SCIENCES | Different Study Outcome |  |  |  |  |
|  |  | 343 | Lafata, J. E., et al. | 2019 | Randomised trial to evaluate the effectiveness and impact of offering postvisit decision support and assistance in obtaining physician-recommended colorectal cancer screening: the e-assist: Colon Health study - a protocol study | BMJ Open | Different Study Outcome |  |  |  |  |
|  |  | 344 | Lai, Y. K., et al. | 2024 | From Online to Offline: How Different Sources of Online Health Information Seeking Affect Patient-Centered Communication in Chinese Older Adults? The Roles of Patient Activation and Patient-Provider Discussion of Online Health Information | Health Communication | Non Original Articles |  |  |  |  |
|  |  | 345 | Lally, R. M., et al. | 2019 | Feasibility and acceptance of the CaringGuidance web-based, distress self-management, psychoeducational program initiated within 12 weeks of breast cancer diagnosis | PSYCHO-ONCOLOGY | Different Study Outcome |  |  |  |  |
|  |  | 346 | Lally, R. M., Kupzyk, K., Gallo, S. & Berry, D. | 2020 | Use of an Unguided, Web-Based Distress Self-Management Program After Breast Cancer Diagnosis: Sub-Analysis of CaringGuidance Pilot Study | Journal of Medical Internet Research | Different Study Design |  |  |  |  |
|  |  | 347 | Lange, L. et al. | 2018 | eHealth interventions and applications in oncology | Onkologe | Non English |  |  |  |  |
|  |  | 348 | Lambert, S. D., Duncan, L. R., Culos-Reed, S. N., Hallward, L., Higano, C. S., Loban, E., Katz, A., De Raad, M., Ellis, J., Korman, M. B., Sears, C., Ibberson, C., Walker, L., Belzile, E., Saha-Chaudhuri, P., McTaggart-Cowan, H. & Peacock, S. | 2022 | Feasibility, Acceptability, and Clinical Significance of a Dyadic, Web-Based, Psychosocial and Physical Activity Self-Management Program (TEMPO) Tailored to the Needs of Men with Prostate Cancer and Their Caregivers: A Multi-Center Randomized Pilot Trial | CURRENT ONCOLOGY | Different Study Outcome |  |  |  |  |
|  |  | 349 | Lambert, S. D., Duncan, L. R., Culos-Reed, S. N., Hallward, L., Higano, C. S., Loban, E., Katz, A., De Raad, M., Ellis, J., Korman, M. B., Sears, C., Ibberson, C., Walker, L., Belzile, E., Saha-Chaudhuri, P., McTaggart-Cowan, H. & Peacock, S. | 2022 | Feasibility, Acceptability, and Clinical Significance of a Dyadic, Web-Based, Psychosocial and Physical Activity Self-Management Program (TEMPO) Tailored to the Needs of Men with Prostate Cancer and Their Caregivers: A Multi-Center Randomized Pilot Trial | CURRENT ONCOLOGY | Duplicate |  |  |  |  |
|  |  | 350 | Langius-Eklöf, A., Crafoord, M. T., Christiansen, M., Fjell, M. & Sundberg, K. | 2017 | Effects of an interactive mHealth innovation for early detection of patient-reported symptom distress with focus on participatory care: Protocol for a study based on prospective, randomised, controlled trials in patients with prostate and breast cancer | BMC Cancer | Different Study Outcome |  |  |  |  |
|  |  | 351 | Langius-Eklöf, A., Crafoord, M. T., Christiansen, M., Fjell, M. & Sundberg, K. | 2017 | Effects of an interactive mHealth innovation for early detection of patient-reported symptom distress with focus on participatory care: Protocol for a study based on prospective, randomised, controlled trials in patients with prostate and breast cancer | BMC Cancer | Duplicate |  |  |  |  |
|  |  | 352 | Lee, B. E., Uhm, J. Y. & Kim, M. S. | 2023 | Effects of social support and self-efficacy on eHealth literacy in Korean women undergoing breast cancer treatment: A secondary analysis | ASIA-PACIFIC JOURNAL OF ONCOLOGY NURSING | Different Study Design |  |  |  |  |
|  |  | 353 | Lee, C. J., Gray, S. W. & Lewis, N. | 2010 | Internet use leads cancer patients to be active health care consumers | Patient Education and Counseling | Different Study Outcome |  |  |  |  |
|  |  | 354 | Lee, E. W. J., Mccloud, R. F. & Viswanath, K. | 2022 | Designing Effective eHealth Interventions for Underserved Groups: Five Lessons From a Decade of eHealth Intervention Design and Deployment | Journal of Medical Internet Research | Different Study Outcome |  |  |  |  |
|  |  | 355 | Lee, H., Ghebre, R., Le, C., Jang, Y. J., Sharratt, M. & Yee, D. | 2017 | Mobile Phone Multilevel and Multimedia Messaging Intervention for Breast Cancer Screening: Pilot Randomized Controlled Trial | JMIR mHealth and uHealth | Different Study Outcome |  |  |  |  |
|  |  | 356 | Lee, H. Y., Koopmeiners, J. S., Rhee, T. G., Raveis, V. H. & Ahluwalia, J. S. | 2014 | Mobile Phone Text Messaging Intervention for Cervical Cancer Screening: Changes in Knowledge and Behavior Pre-Post Intervention | Journal of Medical Internet Research | Different Study Outcome |  |  |  |  |
|  |  | 357 | Lee, H. Y., Lee, M. H., Gao, Z. & Sadak, K. | 2018 | Development and Evaluation of Culturally and Linguistically Tailored Mobile App to Promote Breast Cancer Screening | Journal of Clinical Medicine | Different Study Outcome |  |  |  |  |
|  |  | 358 | Légaré, F., Robitaille, H., Gane, C., Hébert, J., Labrecque, M. & Rousseau, F. | 2016 | Improving Decision Making about Genetic Testing in the Clinic: An Overview of Effective Knowledge Translation Interventions | PLoS ONE | Different Study Outcome |  |  |  |  |
|  |  | 359 | Lemasters, T., Madhavan, S., Atkins, E., Vyas, A., Remick, S. & Vona-Davis, L. | 2014 | "Don't Know" and Accuracy of Breast Cancer Risk Perceptions Among Appalachian Women Attending a Mobile Mammography Program: Implications for Educational Interventions and Patient Empowerment | Journal of Cancer Education | Different Study Outcome |  |  |  |  |
|  |  | 360 | Lemasters, T., Madhavan, S., Atkins, E., Vyas, A., Remick, S. & Vona-Davis, L. | 2014 | "Don't Know" and Accuracy of Breast Cancer Risk Perceptions Among Appalachian Women Attending a Mobile Mammography Program: Implications for Educational Interventions and Patient Empowerment | Journal of Cancer Education | Duplicate |  |  |  |  |
|  |  | 361 | Leske, M., Koczwara, B., Morris, J., & Beatty, L. | 2023 | Modality Preferences for Health Behaviour Interventions for Post-Treatment Cancer Survivors: A Theoretical Investigation. | *Supportive Care in Cancer* | Different Study Outcome |  |  |  |  |
|  |  | 362 | Levy-Storms, L., Bastani, R., & Reuben, D. B. | 2004 | Predictors of Varying Levels of Nonadherence to Mammography Screening in Older Women. | *Journal of the American Geriatrics Society* | Different Study Outcome |  |  |  |  |
|  |  | 363 | Li, D., Kong, J., Yang, Y., Wang, S. L., Wu, J. P., & Chao, J. Q. | 2019 | Group Cognitive Behavioural Therapy of Physical and Psychological Menopausal Symptoms of Chinese Women, Delivered via Internet and Mobile Phone Versus Face to Face: A Protocol for a Randomized Non-Inferiority Trial. | *Maturitas* | Different Study Outcome |  |  |  |  |
|  |  | 364 | Li, F., Li, M., Guan, P., Ma, S., & Cui, L. | 2015 | Mapping Publication Trends and Identifying Hot Spots of Research on Internet Health Information Seeking Behavior: A Quantitative and Co-Word Biclustering Analysis. | Journal of medical Internet research | Different Study Design |  |  |  |  |
|  |  | 365 | Li, L. J., Wang, L., Sun, Q., Xiao, P. P., Duan, Y. L., et al. | 2022 | Effect of Two Interventions on Sleep Quality for Adolescent and Young Adult Cancer Survivors: A Pilot Randomized Controlled Trial. | *Cancer Nursing* | Different Study Outcome |  |  |  |  |
|  |  | 366 | Li, X., Zhang, N., Yang, J., Geng, Z., Zhou, J., & Zhang, J. | 2024 | Weight Management Personas of Breast Cancer Patients Undergoing Chemotherapy in China: A Multi-Method Study. | *BMC Medical Informatics and Decision Making* | Different Study Outcome |  |  |  |  |
|  |  | 367 | Li, X. Y., Zhang, N., Yang, J., Geng, Z. H., Zhou, J., & Zhang, J. Y. | 2024 | Weight Management Personas of Breast Cancer Patients Undergoing Chemotherapy in China: A Multi-Method Study. | *BMC Medical Informatics and Decision Making* | Duplicate |  |  |  |  |
|  |  | 368 | Lim, J. W., Baik, O. M., & Ashing-Giwa, K. T. | 2012 | Cultural Health Beliefs and Health Behaviors in Asian American Breast Cancer Survivors: A Mixed-Methods Approach. | *Oncology Nursing Forum* | Different Study Outcome |  |  |  |  |
|  |  | 369 | Lim, J. Y., Kim, Y., Yeo, S. M., Chae, B. J., Yu, J., & Hwang, J. H. | 2023 | Feasibility and Usability of a Personalized mHealth App for Self-Management in the First Year Following Breast Cancer Surgery. | *Health Informatics Journal* | Different Study Outcome |  |  |  |  |
|  |  | 370 | Liou, K., Mcconnell, K. M., Currier, M. B., Baser, R. E., et al. | 2023 | Telehealth-Based Music Therapy Versus Cognitive Behavioral Therapy for Anxiety in Cancer Survivors: Rationale and Protocol for a Comparative Effectiveness Trial. | *JMIR Research Protocols* | Different Study Outcome |  |  |  |  |
|  |  | 371 | Liu, P. L., Ye, J. F., Ao, H. S., Sun, S. X., Zheng, Y., et al. | 2023 | Effects of Electronic Personal Health Information Technology on American Women's Cancer Screening Behaviors Mediated Through Cancer Worry. | *Digital Health* | Different Study Outcome |  |  |  |  |
|  |  | 372 | Liu, Z. L., Liao, Y., Hwang, C. L., Rethorst, C. D., & Zhang, X. L. | 2024 | Associations of Online Health Information Seeking with Health Behaviors of Cancer Survivors. | *Digital Health* | Different Study Outcome |  |  |  |  |
|  |  | 373 | Loerzel, V., Alamian, A., Clochesy, J., & Geddie, P. | 2024 | Serious Gaming for Chemotherapy-Induced Nausea and Vomiting in Older Adults With Cancer: Protocol for a Randomized Clinical Trial. | *JMIR Research Protocols* | Different Study Outcome |  |  |  |  |
|  |  | 374 | Lohr, A. M., Capetillo, G. P., Molina, L., et al. | 2024 | Development of a Digital Storytelling Intervention to Increase Breast, Cervical, and Colorectal Cancer Screening in the Hispanic/Latino Community: A Qualitative Evaluation. | *Journal of Cancer Education* | Different Study Outcome |  |  |  |  |
|  |  | 375 | Lohr, A. M., Capetillo, G. P., Molina, L., et al. | 2024 | Development of a Digital Storytelling Intervention to Increase Breast, Cervical, and Colorectal Cancer Screening in the Hispanic/Latino Community: A Qualitative Evaluation. | *Journal of Cancer Education* | Duplicate |  |  |  |  |
|  |  | 376 | Lopez-Pentecost, M., Perkin, S., Freylersythe, S., et al. | 2023 | Feasibility and Acceptability of a Text Message Intervention to Promote Adherence to Nutrition and Physical Activity Guidelines in a Predominantly Hispanic Sample of Cancer Survivors and Their Informal Caregivers. | *Nutrients* | Different Study Outcome |  |  |  |  |
|  |  | 377 | Lowry, K. P., Trentham-Dietz, A., Schechter, C. B., et al. | 2020 | Long-Term Outcomes and Cost-Effectiveness of Breast Cancer Screening With Digital Breast Tomosynthesis in the United States. | *JNCI - Journal of the National Cancer Institute* | Different Study Outcome |  |  |  |  |
|  |  | 378 | Loy, M. H., Prisco, L., & Parikh, C. | 2024 | Implementation of Virtual Integrative Oncology Shared Medical Appointment Series (VIOSMAS) Within Mixed Diagnosis Population. | *Integrative Cancer Therapies* | Different Study Outcome |  |  |  |  |
|  |  | 379 | Loy, M. H., Prisco, L., & Parikh, C. | 2024 | Implementation of Virtual Integrative Oncology Shared Medical Appointment Series (VIOSMAS) Within Mixed Diagnosis Population. | *Integrative Cancer Therapies* | Duplicate |  |  |  |  |
|  |  | 380 | Lozano-Lozano, M., Martín-Martín, L., Galiano-Castillo, N., et al. | 2016 | Integral Strategy to Supportive Care in Breast Cancer Survivors Through Occupational Therapy and a mHealth System: Design of a Randomized Clinical Trial. | *BMC Medical Informatics and Decision Making* | Different Study Outcome |  |  |  |  |
|  |  | 381 | Ludwigson, A., Huynh, V., Myers, S., et al. | 2022 | Patient Perceptions of Changes in Breast Cancer Care and Well-Being During COVID-19: A Mixed Methods Study. | *Annals of Surgical Oncology* | Different Study Outcome |  |  |  |  |
|  |  | 382 | Ludwigson, A., Huynh, V., Myers, S., et al. | 2022 | Patient Perceptions of Changes in Breast Cancer Care and Well-Being During COVID-19: A Mixed Methods Study. | *Annals of Surgical Oncology* | Duplicate |  |  |  |  |
|  |  | 383 | Luo, C., Zhang, Z., & Jin, J. | 2023 | Recommending Breast Cancer Screening to My Mum: Examining the Interplay of Threat, Efficacy, and Virality on Recommendation Intention in the Chinese Context. | *International Journal of Environmental Research and Public Health* | Different Study Outcome |  |  |  |  |
|  |  | 384 | Luo, C., Zhang, Z., & Jin, J. | 2023 | Recommending Breast Cancer Screening to My Mum: Examining the Interplay of Threat, Efficacy, and Virality on Recommendation Intention in the Chinese Context. | *International Journal of Environmental Research and Public Health* | Duplicate |  |  |  |  |
|  |  | 385 | Luo, X., Chen, Y., Chen, J., et al. | 2022 | Effectiveness of mobile health-based self-management interventions in breast cancer patients: a meta-analysis | Supportive Care in Cancer | Non Original Articles |  |  |  |  |
|  |  | 386 | Lyhne, J. D., Smith, A., Frostholm, L., et al. | 2020 | Study Protocol: A Randomized Controlled Trial Comparing the Efficacy of Therapist-Guided Internet-Delivered Cognitive Therapy (TG-iConquerFear) with Augmented Treatment as Usual in Reducing Fear of Cancer Recurrence in Danish Colorectal Cancer Survivors. | *BMC Cancer* | Duplicate |  |  |  |  |
|  |  | 387 | Lyhne, J. D., Smith, A., Frostholm, L., et al. | 2020 | Study Protocol: A Randomized Controlled Trial Comparing the Efficacy of Therapist-Guided Internet-Delivered Cognitive Therapy (TG-iConquerFear) with Augmented Treatment as Usual in Reducing Fear of Cancer Recurrence in Danish Colorectal Cancer Survivors. | *BMC Cancer* | Different Study Outcome |  |  |  |  |
|  |  | 388 | Lyons, E. J., Baranowski, T., Basen-Engquist, K. M., et al. | 2016 | Testing the Effects of Narrative and Play on Physical Activity Among Breast Cancer Survivors Using Mobile Apps: Study Protocol for a Randomized Controlled Trial. | *BMC Cancer* | Different Study Outcome |  |  |  |  |
|  |  | 389 | Madl, M., et al. | 2022 | The influence of patient-related factors on the frequency and duration of psycho-oncological sessions in a university cancer center | Journal of Psychosocial Oncology | Different Study Outcome |  |  |  |  |
|  |  | 390 | Malandrone, F., et al. | 2024 | Exploring the Effects of Variety and Amount of Mindfulness Practices on Depression, Anxiety, and Stress Symptoms: Longitudinal Study on a Mental Health-Focused eHealth System for Patients With Breast or Prostate Cancer | JMIR Mental Health | Different Study Outcome |  |  |  |  |
|  |  | 391 | Malandrone, F., et al. | 2024 | Exploring the Effects of Variety and Amount of Mindfulness Practices on Depression, Anxiety, and Stress Symptoms: Longitudinal Study on a Mental Health-Focused eHealth System for Patients With Breast or Prostate Cancer | JMIR Mental Health | Duplicate |  |  |  |  |
|  |  | 392 | Malandrone, F., et al. | 2024 | Exploring the Effects of Variety and Amount of Mindfulness Practices on Depression, Anxiety, and Stress Symptoms: Longitudinal Study on a Mental Health-Focused eHealth System for Patients With Breast or Prostate Cancer | JMIR Mental Health | Duplicate |  |  |  |  |
|  |  | 393 | Mallah, M. A., et al. | 2022 | Polycyclic aromatic hydrocarbon and its effects on human health: An overeview | CHEMOSPHERE | Non Original Articles |  |  |  |  |
|  |  | 394 | Mallaiah, S., et al. | 2022 | Yoga Therapy in Cancer Care via Telehealth During the COVID-19 Pandemic | Integrative Cancer Therapies | Different Study Outcome |  |  |  |  |
|  |  | 395 | Mallaiah, S., et al. | 2022 | Yoga Therapy in Cancer Care via Telehealth During the COVID-19 Pandemic | Integrative Cancer Therapies | Duplicate |  |  |  |  |
|  |  | 396 | Mallmann, C. A., et al. | 2021 | Digital Technical and Informal Resources of Breast Cancer Patients From 2012 to 2020: Questionnaire-Based Longitudinal Trend Study | JMIR Cancer | Different Study Outcome |  |  |  |  |
|  |  | 397 | Mallmann, C. A., et al. | 2021 | Digital Technical and Informal Resources of Breast Cancer Patients From 2012 to 2020: Questionnaire-Based Longitudinal Trend Study | JMIR Cancer | Duplicate |  |  |  |  |
|  |  | 398 | Marcu, A., et al. | 2019 | Online Information-Seeking About Potential Breast Cancer Symptoms: Capturing Online Behavior With an Internet Browsing Tracking Tool | Journal of Medical Internet Research | Different Study Outcome |  |  |  |  |
|  |  | 399 | Marcu, A., et al. | 2019 | Online Information-Seeking About Potential Breast Cancer Symptoms: Capturing Online Behavior With an Internet Browsing Tracking Tool | Journal of Medical Internet Research | Duplicate |  |  |  |  |
|  |  | 400 | Marsch, L. A. | 2021 | Digital health data-driven approaches to understand human behavior | Neuropsychopharmacology | Different Study Outcome |  |  |  |  |
|  |  | 401 | Marshall-McKenna, R., et al. | 2023 | A multinational investigation of healthcare needs, preferences, and expectations in supportive cancer care: co-creating the LifeChamps digital platform | Journal of Cancer Survivorship | Different Study Outcome |  |  |  |  |
|  |  | 402 | Marshall-McKenna, R., et al. | 2023 | A multinational investigation of healthcare needs, preferences, and expectations in supportive cancer care: co-creating the LifeChamps digital platform | Journal of Cancer Survivorship | Duplicate |  |  |  |  |
|  |  | 403 | Marthick, M., et al. | 2019 | Feasibility of an Interactive Patient Portal in Oncology: Qualitative Study | JMIR Cancer | Different Study Outcome |  |  |  |  |
|  |  | 404 | Martí-Bonmatí, L | 2018 | How to integrate quantitative information into imaging reports for oncologic patients | RADIOLOGIA | Non English |  |  |  |  |
|  |  | 405 | Martin, E., et al. | 2021 | Use of mHealth to Increase Physical Activity Among Breast Cancer Survivors With Fatigue: Qualitative Exploration | JMIR Cancer | Different Study Outcome |  |  |  |  |
|  |  | 406 | Martin, E., et al. | 2021 | Use of mHealth to Increase Physical Activity Among Breast Cancer Survivors With Fatigue: Qualitative Exploration | JMIR Cancer | Duplicate |  |  |  |  |
|  |  | 407 | Martin, E., et al. | 2021 | Use of mHealth to Increase Physical Activity Among Breast Cancer Survivors With Fatigue: Qualitative Exploration | JMIR Cancer | Duplicate |  |  |  |  |
|  |  | 408 | Martín-Payo, R., et al. | 2023 | Use a web-app to improve breast cancer risk factors and symptoms knowledge and adherence to healthy diet and physical activity in women without breast cancer diagnosis (Precam project) | Cancer Causes & Control | Different Study Outcome |  |  |  |  |
|  |  | 409 | Martín-Payo, R., et al. | 2023 | Use a web-app to improve breast cancer risk factors and symptoms knowledge and adherence to healthy diet and physical activity in women without breast cancer diagnosis (Precam project) | Cancer Causes & Control | Duplicate |  |  |  |  |
|  |  | 410 | Masiero, M., et al. | 2024 | Adherence to oral anticancer treatments: network and sentiment analysis exploring perceived internal and external determinants in patients with metastatic breast cancer | Supportive Care in Cancer | Different Study Design |  |  |  |  |
|  |  | 411 | Mayer, D. K., et al. | 2018 | SurvivorCHESS to increase physical activity in colon cancer survivors: can we get them moving? | Journal of Cancer Survivorship | Different Study Outcome |  |  |  |  |
|  |  | 412 | McCarthy, M. S., et al. | 2018 | Feasibility of a Telemedicine-Delivered Cognitive Behavioral Therapy for Insomnia in Rural Breast Cancer Survivors | Oncology Nursing Forum | Different Study Outcome |  |  |  |  |
|  |  | 413 | McGrady, M. E., et al. | 2024 | Psychological Outcomes in Adolescent and Young Adult Cancer Survivors | Journal of Clinical Oncology | Different Study Outcome |  |  |  |  |
|  |  | 414 | Medina, J. C., et al. | 2022 | ICOnnecta't: Development and Initial Results of a Stepped Psychosocial eHealth Ecosystem to Facilitate Risk Assessment and Prevention of Early Emotional Distress in Breast Cancer Survivors' Journey | Cancers | Different Study Outcome |  |  |  |  |
|  |  | 415 | Meeking, K. | 2020 | Patients' experiences of radiotherapy: Insights from Twitter | Radiography | Different Study Outcome |  |  |  |  |
|  |  | 416 | Melhem, S. J., et al. | 2023 | Digital Trends, Digital Literacy, and E-Health Engagement Predictors of Breast and Colorectal Cancer Survivors: A Population-Based Cross-Sectional Survey | International Journal of Environmental Research and Public Health | Different Study Outcome |  |  |  |  |
|  |  | 417 | Mendes-Santos, C., et al. | 2022 | Development and Evaluation of the Usefulness, Usability, and Feasibility of iNNOV Breast Cancer: Mixed Methods Study | JMIR Cancer | Different Study Outcome |  |  |  |  |
|  |  | 418 | Meneses, K., et al. | 2018 | Telehealth intervention for Latina breast cancer survivors: A pilot | Women's Health | Included |  |  |  |  |
|  |  | 419 | Mifsud, A., et al. | 2021 | Feasibility and pilot study of a brief self-compassion intervention addressing body image distress in breast cancer survivors | Health Psychology and Behavioral Medicine | Different Study Outcome |  |  |  |  |
|  |  | 420 | Mlakar, I., et al. | 2021 | Patients-centered SurvivorShIp care plan after Cancer treatments based on Big Data and Artificial Intelligence technologies (PERSIST): a multicenter study protocol to evaluate efficacy of digital tools supporting cancer survivors | BMC Medical Informatics and Decision Making | Different Study Outcome |  |  |  |  |
|  |  | 421 | Molina, Y., et al. | 2015 | Medical Advocacy and Supportive Environments for African-Americans Following Abnormal Mammograms | Journal of Cancer Education | Different Study Outcome |  |  |  |  |
|  |  | 422 | Moller, D., Conrad, J. | 2010 | Electrical glitch | Core Clinical Competencies in Anesthesiology: A Case-Based Approach | Non Original Articles |  |  |  |  |
|  |  | 423 | Molero-Castillo, G., Jasso-Villazul, J., Torres-Vargas, A., Velázquez-Mena, A | 2020 | Towards the processes discovery in the medical treatment of mexican-origin women diagnosed with breast cancer | Lecture Notes in Networks and Systems | Non Original Articles |  |  |  |  |
|  |  | 424 | Monteiro-Guerra, F., et al. | 2020 | A personalized physical activity coaching app for breast cancer survivors: Design process and early prototype testing | JMIR mHealth and uHealth | Different Study Outcome |  |  |  |  |
|  |  | 425 | Monteiro-Guerra, F., et al. | 2020 | A Personalized Physical Activity Coaching App for Breast Cancer Survivors: Design Process and Early Prototype Testing | JMIR mHealth and uHealth | Duplicate |  |  |  |  |
|  |  | 426 | Mooney, K., et al. | 2024 | Assessing Patient Perspectives and the Health Equity of a Digital Cancer Symptom Remote Monitoring and Management System | JCO Clinical Cancer Informatics | Different Study Outcome |  |  |  |  |
|  |  | 427 | Moraitis, A. M., et al. | 2023 | Feasibility and acceptability of an mHealth, home-based exercise intervention in colorectal cancer survivors: A pilot randomized controlled trial | PLoS ONE | Different Study Outcome |  |  |  |  |
|  |  | 428 | Moraliyage, H., et al. | 2021 | Cancer in Lockdown: Impact of the COVID-19 Pandemic on Patients with Cancer | Oncologist | Different Study Outcome |  |  |  |  |
|  |  | 429 | Moravac, C. C. | 2018 | Reflections of Homeless Women and Women with Mental Health Challenges on Breast and Cervical Cancer Screening Decisions: Power, Trust, and Communication with Care Providers | Frontiers in Public Health | Duplicate |  |  |  |  |
|  |  | 430 | Moravac, C. C. | 2018 | Reflections of Homeless Women and Women with Mental Health Challenges on Breast and Cervical Cancer Screening Decisions: Power, Trust, and Communication with Care Providers | Frontiers in Public Health | Different Study Outcome |  |  |  |  |
|  |  | 431 | Morena, N., et al. | 2023 | Content Quality of YouTube Videos About Metastatic Breast Cancer in Young Women: Systematic Assessment | JMIR Cancer | Different Study Design |  |  |  |  |
|  |  | 432 | Moreno-Gutierrez, S., et al. | 2021 | ATOPE plus: An mHealth System to Support Personalized Therapeutic Exercise Interventions in Patients With Cancer | IEEE Access | Different Study Outcome |  |  |  |  |
|  |  | 433 | Murad, M. F., et al. | 2014 | Teleoncology: Improving Patient Outcome Through Coordinated Care | Telemedicine and e-Health | Different Study Outcome |  |  |  |  |
|  |  | 434 | Myers, C., et al. | 2024 | Lessons learned from COVID-19: improving breast cancer care post-pandemic from the patient perspective | Supportive Care in Cancer | Different Study Outcome |  |  |  |  |
|  |  | 435 | Myers, C., et al. | 2024 | Lessons learned from COVID-19: improving breast cancer care post-pandemic from the patient perspective | Supportive Care in Cancer | Duplicate |  |  |  |  |
|  |  | 436 | Myers, C., et al. | 2024 | Lessons learned from COVID-19: improving breast cancer care post-pandemic from the patient perspective | Supportive Care in Cancer | Duplicate |  |  |  |  |
|  |  | 437 | Myneni, S., et al. | 2016 | In Pursuit of Theoretical Ground in Behavior Change Support Systems: Analysis of Peer-to-Peer Communication in a Health-Related Online Community | Journal of Medical Internet Research | Different Study Design |  |  |  |  |
|  |  | 438 | Nadhamuni, S., Gupta, G., Mullath, R., Sridhar, S. | 2020 | Leveraging Technology to Enable Effective Preventive Screening of NCDs at Population Scale: Initial Observations | Journal of the Indian Institute of Science | Non Original Articles |  |  |  |  |
|  |  | 439 | Nahm, E. S., et al. | 2019 | Testing the Impact of a Cancer Survivorship Patient Engagement Toolkit on Selected Health Outcomes | Oncology Nursing Forum | Different Study Outcome |  |  |  |  |
|  |  | 440 | Najafi, N., et al. | 2024 | Breamy: An augmented reality mHealth prototype for surgical decision-making in breast cancer | Healthcare Technology Letters | Different Study Outcome |  |  |  |  |
|  |  | 441 | Najafi, N., et al. | 2024 | Breamy: An augmented reality mHealth prototype for surgical decision-making in breast cancer | Healthcare Technology Letters | Duplicate |  |  |  |  |
|  |  | 442 | Najafi, N., et al. | 2024 | Breamy: An augmented reality mHealth prototype for surgical decision-making in breast cancer | Healthcare Technology Letters | Duplicate |  |  |  |  |
|  |  | 443 | Nakao, M., Shirotsuki, K., Sugaya, N. | 2021 | Cognitive–behavioral therapy for management of mental health and stress-related disorders: Recent advances in techniques and technologies | BioPsychoSocial Medicine | Non Original Articles |  |  |  |  |
|  |  | 444 | Namkoong, K., et al. | 2010 | Expression and reception of treatment information in breast cancer support groups: How health self-efficacy moderates effects on emotional well-being | Patient Education and Counseling | Different Study Outcome |  |  |  |  |
|  |  | 445 | Namkoong, K., et al. | 2010 | Expression and reception of treatment information in breast cancer support groups: How health self-efficacy moderates effects on emotional well-being | Patient Education and Counseling | Duplicate |  |  |  |  |
|  |  | 446 | Nápoles, A. M., et al. | 2019 | Feasibility of a mobile phone app and telephone coaching survivorship care planning program among spanish-speaking breast cancer survivors | JMIR Cancer | Different Study Outcome |  |  |  |  |
|  |  | 447 | Nápoles, A. M., et al. | 2019 | Feasibility of a Mobile Phone App and Telephone Coaching Survivorship Care Planning Program Among Spanish-Speaking Breast Cancer Survivors | JMIR Cancer | Duplicate |  |  |  |  |
|  |  | 448 | Narayanan, S., et al. | 2021 | Integrative Oncology Consultations Delivered via Telehealth in 2020 and In-Person in 2019: Paradigm Shift During the COVID-19 World Pandemic | Integrative Cancer Therapies | Different Study Outcome |  |  |  |  |
|  |  | 449 | Narayanan, S., et al. | 2021 | Integrative Oncology Consultations Delivered via Telehealth in 2020 and In-Person in 2019: Paradigm Shift During the COVID-19 World Pandemic | Integrative Cancer Therapies | Duplicate |  |  |  |  |
|  |  | 450 | Narayanan, S., et al. | 2021 | Integrative Oncology Consultations Delivered via Telehealth in 2020 and In-Person in 2019: Paradigm Shift During the COVID-19 World Pandemic | Integrative Cancer Therapies | Duplicate |  |  |  |  |
|  |  | 451 | Nasution, A., et al. | 2024 | Breast Cancer Awareness: Development and Usability of the BrAware Mobile Health Application | Malaysian Journal of Medicine & Health Sciences | Different Study Outcome |  |  |  |  |
|  |  | 452 | Neubert, S., et al. | 2023 | Effects of a video sequence based intervention on anxiety, fatigue and depression in cancer patients: results of a randomized controlled trial | Integrative Cancer Therapies | Different Study Outcome |  |  |  |  |
|  |  | 453 | Ng, A. K. & Hudson, M. M. | 2014 | BioSurveillance and Longitudinal Lifelong Guidelines | ALERT - ADVERSE LATE EFFECTS OF CANCER TREATMENT | Non Original Articles |  |  |  |  |
|  |  | 454 | Nguyen, N. P., et al. | 2023 | Impact of COVID-19 pandemic on older cancer patients: Proposed solution by the International Geriatric Radiotherapy Group | Frontiers in Oncology | Different Study Outcome |  |  |  |  |
|  |  | 455 | Ni, C. X., et al. | 2024 | A Mobile Applet for Assessing Medication Adherence and Managing Adverse Drug Reactions Among Patients With Cancer: Usability and Utility Study | JMIR Formative Research | Different Study Outcome |  |  |  |  |
|  |  | 456 | Niu, L., et al. | 2021 | Using Nomogram to Predict the Hospitalization Forgone Among Internal Migrants in China: A Nationally Representative Cross-Sectional Secondary Data Analysis | RISK MANAGEMENT AND HEALTHCARE POLICY | Different Study Design |  |  |  |  |
|  |  | 457 | Nofal, M. R., et al. | 2018 | Unpacking the "Black Box": How an SMS-Based Continuing Medical Education Intervention Improved Medical Knowledge Among HIV Clinicians in Vietnam | GLOBAL HEALTH-SCIENCE AND PRACTICE | Different Study Outcome |  |  |  |  |
|  |  | 458 | Ochoa-Arnedo, C. | 2020 | E-health iconnecta’t program: An ecosystem to promote wellbing in cancer towards oncommun european proposal | Psicooncologia | Non English |  |  |  |  |
|  |  | 459 | Okyere, J., et al. | 2024 | Clicks and checks: investigating the association between internet usage frequency and women's uptake of clinical breast examination in Ghana | BMC Health Services Research | Different Study Outcome |  |  |  |  |
|  |  | 460 | Onuma, A. E., et al. | 2019 | Patient preferences on the use of technology in cancer surveillance after curative surgery: A cross-sectional analysis | SURGERY | Non Original Articles |  |  |  |  |
|  |  | 461 | Oostra, D. L., et al. | 2021 | Understanding Nutritional Problems of Metastatic Breast Cancer Patients Opportunities for Supportive Care Through eHealth | CANCER NURSING | Different Study Outcome |  |  |  |  |
|  |  | 462 | Oostra, D. L., et al. | 2021 | Understanding Nutritional Problems of Metastatic Breast Cancer Patients Opportunities for Supportive Care Through eHealth | CANCER NURSING | Duplicate |  |  |  |  |
|  |  | 463 | Oppong, B. A., et al. | 2023 | Utilization of cancer survivorship services during the COVID-19 pandemic in a tertiary referral center | Journal of Cancer Survivorship | Different Study Outcome |  |  |  |  |
|  |  | 464 | Ormel, I., et al. | 2021 | Using a Mobile App-Based Video Recommender System of Patient Narratives to Prepare Women for Breast Cancer Surgery: Development and Usability Study Informed by Qualitative Data | JMIR Formative Research | Different Study Outcome |  |  |  |  |
|  |  | 465 | Ouyang, W., Xie, W., Xin, Z., et al. | 2021 | Evolutionary overview of consumer health informatics: Bibliometric study on the web of science from 1999 to 2019 | Journal of Medical Internet Research | Non Original Articles |  |  |  |  |
|  |  | 466 | Owen, J. E., et al. | 2016 | Characterizing Social Networks and Communication Channels in a Web-Based Peer Support Intervention | CYBERPSYCHOLOGY BEHAVIOR AND SOCIAL NETWORKING | Different Study Outcome |  |  |  |  |
|  |  | 467 | Pace, T. W. W., et al. | 2022 | Feasibility, Acceptability, and Preliminary Efficacy of an App-Based Meditation Intervention to Decrease Firefighter Psychological Distress and Burnout: A One-Group Pilot Study | JMIR Formative Research | Different Study Outcome |  |  |  |  |
|  |  | 468 | Pagkatipunan, P. M. N. | 2018 | Peer leaders and phone prompts: Implications in the practice of breast care among college students | Asian Pacific Journal of Cancer Prevention | Different Study Outcome |  |  |  |  |
|  |  | 469 | Pan, Y., et al. | 2022 | Research Trends around Exercise Rehabilitation among Cancer Patients: A Bibliometrics and Visualized Knowledge Graph Analysis | BioMed Research International | Different Study Design |  |  |  |  |
|  |  | 470 | Pan, Y., et al. | 2022 | Research Trends around Exercise Rehabilitation among Cancer Patients: A Bibliometrics and Visualized Knowledge Graph Analysis | BioMed Research International | Duplicate |  |  |  |  |
|  |  | 471 | Pan, Y., et al. | 2022 | Research Trends around Exercise Rehabilitation among Cancer Patients: A Bibliometrics and Visualized Knowledge Graph Analysis | BioMed Research International | Duplicate |  |  |  |  |
|  |  | 472 | Park, J. H., et al. | 2012 | Quality of life and symptom experience in breast cancer survivors after participating in a psychoeducational support program: a pilot study | CANCER NURSING | Different Study Outcome |  |  |  |  |
|  |  | 473 | Park, S. W., et al. | 2019 | Factors associated with physical activity of breast cancer patients participating in exercise intervention | Supportive Care in Cancer | Different Study Outcome |  |  |  |  |
|  |  | 474 | Parrocha, A. N. & Bernadas, J. | 2024 | 'They Know What It's Like': Exploring Facebook Groups for Digital Coping | JOURNAL OF CREATIVE COMMUNICATIONS | Different Study Outcome |  |  |  |  |
|  |  | 475 | Patrick-Miller, L. J., et al. | 2014 | Development of a Communication Protocol for Telephone Disclosure of Genetic Test Results for Cancer Predisposition | JMIR Research Protocols | Different Study Outcome |  |  |  |  |
|  |  | 476 | Payo, R. M., et al. | 2019 | Prescribing fitness apps for people with cancer: a preliminary assessment of content and quality of commercially available apps | Journal of Cancer Survivorship | Different Study Outcome |  |  |  |  |
|  |  | 477 | Peek, M. E. & Han, J. | 2007 | Mobile mammography: Assessment of self-referral in reaching medically underserved women | JOURNAL OF THE NATIONAL MEDICAL ASSOCIATION | Different Study Outcome |  |  |  |  |
|  |  | 478 | Peersmann, S. H. M., et al. | 2021 | Does the guided online cognitive behavioral therapy for insomnia "i-Sleep youth" improve sleep of adolescents and young adults with insomnia after childhood cancer? (MICADO-study): study protocol of a randomized controlled trial | Trials | Different Study Outcome |  |  |  |  |
|  |  | 479 | Pembroke, L., et al. | 2024 | Online Group Cognitive Rehabilitation Program for Prostate Cancer Survivors: Development Using Codesign and the Theoretical Domains Framework | SEMINARS IN ONCOLOGY NURSING | Different Study Outcome |  |  |  |  |
|  |  | 480 | Persky, S., et al. | 2019 | Internet Versus Virtual Reality Settings for Genomics Information Provision | CYBERPSYCHOLOGY BEHAVIOR AND SOCIAL NETWORKING | Different Study Outcome |  |  |  |  |
|  |  | 481 | Pesapane, F., Abbate, F., Bozzini, A., et al. | 2022 | What breast radiologists have learned from the COVID-19 pandemic | Journal of Public Health and Emergency | Non Original Articles |  |  |  |  |
|  |  | 482 | Petros, N. G., et al. | 2023 | Predictors of the Use of a Mental Health-Focused eHealth System in Patients With Breast and Prostate Cancer: Bayesian Structural Equation Modeling Analysis of a Prospective Study | JMIR Cancer | Different Study Design |  |  |  |  |
|  |  | 483 | Petros, N. G., et al. | 2023 | Predictors of the Use of a Mental Health–Focused eHealth System in Patients With Breast and Prostate Cancer: Bayesian Structural Equation Modeling Analysis of a Prospective Study | JMIR Cancer | Duplicate |  |  |  |  |
|  |  | 484 | Petros, N. G., et al. | 2022 | Sociodemographic Characteristics Associated With an eHealth System Designed to Reduce Depressive Symptoms Among Patients With Breast or Prostate Cancer: Prospective Study | JMIR Formative Research | Different Study Outcome |  |  |  |  |
|  |  | 485 | Petros, N. G., et al. | 2022 | Sociodemographic Characteristics Associated With an eHealth System Designed to Reduce Depressive Symptoms Among Patients With Breast or Prostate Cancer: Prospective Study | JMIR Formative Research | Duplicate |  |  |  |  |
|  |  | 486 | Pfirrmann, D., et al. | 2018 | Applicability of a Web-Based, Individualized Exercise Intervention in Patients With Liver Disease, Cystic Fibrosis, Esophageal Cancer, and Psychiatric Disorders: Process Evaluation of 4 Ongoing Clinical Trials | JMIR Research Protocols | Different Study Outcome |  |  |  |  |
|  |  | 487 | Phillips, S., et al. | 2021 | A Technology-Based Physical Activity Intervention for Patients With Metastatic Breast Cancer (Fit2ThriveMB): Protocol for a Randomized Controlled Trial | JMIR Research Protocols | Different Study Outcome |  |  |  |  |
|  |  | 488 | Phillips, S. M., et al. | 2017 | Breast cancer survivors' preferences for technology-supported exercise interventions | Supportive Care in Cancer | Different Study Outcome |  |  |  |  |
|  |  | 489 | Phillips, S. M., et al. | 2022 | Optimization of a technology-supported physical activity promotion intervention for breast cancer survivors: Results from Fit2Thrive | Cancer | Different Study Outcome |  |  |  |  |
|  |  | 490 | Piccolo, C. L., et al. | 2024 | The Correlation between Morpho-Dynamic Contrast-Enhanced Mammography (CEM) Features and Prognostic Factors in Breast Cancer: A Single-Center Retrospective Analysis | Cancers | Different Study Design |  |  |  |  |
|  |  | 491 | Pichardo, M.S., Sanft, T., Ferrucci, L.M., et al. | 2023 | Diet and physical activity interventions in Black and Latina women with breast cancer: A scoping review | Frontiers in Oncology | Non Original Articles |  |  |  |  |
|  |  | 492 | Piché, A., et al. | 2023 | Assessing real-world implementability of a multimodal group-based tele-prehabilitation program in cancer care: a pragmatic feasibility study | Frontiers in Oncology | Different Study Outcome |  |  |  |  |
|  |  | 493 | Pisano, E. D., et al. | 1995 | PATIENT COMPLIANCE IN MOBILE SCREENING MAMMOGRAPHY | ACADEMIC RADIOLOGY | Different Study Outcome |  |  |  |  |
|  |  | 494 | Poikonen-Saksela, P., et al. | 2024 | Digital Self-Management Intervention Paths for Early Breast Cancer Patients: Results of a Pilot Study | BREAST JOURNAL | Different Study Outcome |  |  |  |  |
|  |  | 495 | Poikonen-Saksela, P., et al. | 2024 | Digital Self-Management Intervention Paths for Early Breast Cancer Patients: Results of a Pilot Study | BREAST JOURNAL | Duplicate |  |  |  |  |
|  |  | 496 | Ponder, M., et al. | 2021 | Mobile Health Application for Patients Undergoing Breast Cancer Surgery: Feasibility Study | BREAST JOURNAL | Different Study Outcome |  |  |  |  |
|  |  | 497 | Ponder, M., et al. | 2021 | Mobile Health Application for Patients Undergoing Breast Cancer Surgery: Feasibility Study | JCO Oncology Practice | Duplicate |  |  |  |  |
|  |  | 498 | Porter, K. J., et al. | 2021 | A Novel Behavioral Intervention for Rural Appalachian Cancer Survivors (weSurvive): Participatory Development and Proof-of-Concept Testing | JMIR Cancer | Different Study Outcome |  |  |  |  |
|  |  | 499 | Pourat, N., et al. | 2022 | Trends in access to care among rural patients served at HRSA-funded health centers | JOURNAL OF RURAL HEALTH | Different Study Outcome |  |  |  |  |
|  |  | 500 | Posadzki, P., Mastellos, N., Ryan, R., et al. | 2016 | Automated telephone communication systems for preventive healthcare and management of long-term conditions | Cochrane Database of Systematic Reviews | Non Original Articles |  |  |  |  |
|  |  | 501 | Power, J. M., et al. | 2020 | Experiences of African American breast cancer survivors using digital scales and activity trackers in a weight gain prevention intervention: Qualitative study | JMIR mHealth and uHealth | Different Study Design |  |  |  |  |
|  |  | 502 | Power, J. M., et al. | 2020 | Experiences of African American Breast Cancer Survivors Using Digital Scales and Activity Trackers in a Weight Gain Prevention Intervention: Qualitative Study | JMIR mHealth and uHealth | Duplicate |  |  |  |  |
|  |  | 503 | Price, J. & Brunet, J. | 2022 | Understanding rural-living young adult cancer survivors' motivation during a telehealth behavior change intervention within a single-arm feasibility trial | Health Informatics Journal | Different Study Design |  |  |  |  |
|  |  | 504 | Prodhan, A., et al. | 2023 | Breast Cancer Management in the Era of Covid-19; Key Issues, Contemporary Strategies, and Future Implications | Breast Cancer Targets and Therap | Different Study Design |  |  |  |  |
|  |  | 505 | Pruss, M., et al. | 2024 | Retrospective Impact of COVID-19 Pandemic on Primary Breast Cancer Care | Breast Care | Different Study Design |  |  |  |  |
|  |  | 506 | Puszkiewicz, P., et al. | 2016 | Assessment of Cancer Survivors' Experiences of Using a Publicly Available Physical Activity Mobile Application | JMIR Cancer | Different Study Design |  |  |  |  |
|  |  | 507 | Pezzolato, M., Marzorati, C., Lanzoni, L., et al. | 2023 | "Interventions to increase adherence to oral therapies in breast cancer patients: A systematic review based on the behavior change technique taxonomy" | Psycho-Oncology | Non Original Articles |  |  |  |  |
|  |  | 508 | Prodhan, A.H.M.S.U., Islam, D.Z., Khandker, S.S., et al. | 2023 | Breast Cancer Management in the Era of Covid-19; Key Issues, Contemporary Strategies, and Future Implications | Breast Cancer: Targets and Therapy | Non Original Articles |  |  |  |  |
|  |  | 509 | Pruss, M., Neubacher, M., Dietzel, F., et al. | 2024 | Retrospective Impact of COVID-19 Pandemic on Primary Breast Cancer Care | Breast Care, 19(5) | Non Original Articles |  |  |  |  |
|  |  | 510 | Qi, Y., et al. | 2024 | Feasibility of an exercise-nutrition-psychology integrated rehabilitation model based on mobile health and virtual reality for cancer patients: a single-center, single-arm, prospective phase II study | BMC PALLIATIVE CARE | Different Study Design |  |  |  |  |
|  |  | 511 | Quintiliani, L. M., et al. | 2016 | Pilot and feasibility test of a mobile health-supported behavioral counseling intervention for weight management among breast cancer survivors | JMIR Cancer | Duplicate |  |  |  |  |
|  |  | 512 | Quintiliani, L. M., et al. | 2016 | Pilot and Feasibility Test of a Mobile Health-Supported Behavioral Counseling Intervention for Weight Management Among Breast Cancer Survivors | JMIR Cancer | Different Study Design |  |  |  |  |
|  |  | 513 | Randles, M., Johnson, P., Radi, N. | 2015 | Assured Decision and Meta-Governance for Mobile Medical Support Systems | Applied Computing in Medicine and Health | Non Original Articles |  |  |  |  |
|  |  | 514 | Rahm, A. K., et al. | 2015 | Implementing an evidence-based breast cancer support and communication tool to newly diagnosed patients as standard care in two institutions | TRANSLATIONAL BEHAVIORAL MEDICINE | Different Study Design |  |  |  |  |
|  |  | 515 | Rakhshanderou, S., et al. | 2020 | Theoretically designed interventions for colorectal cancer prevention: a case of the health belief model | BMC MEDICAL EDUCATION | Different Study Design |  |  |  |  |
|  |  | 516 | Razon, S., et al. | 2019 | Perceptions of Physical Activity Tracking Devices: A Survey Analysis | PHYSICAL EDUCATOR-US | Different Study Design |  |  |  |  |
|  |  | 517 | Reading, J. M., et al. | 2024 | Optimization of a mHealth Physical Activity Promotion Intervention With Mindful Awareness for Young Adult Cancer Survivors: Design and Methods of Opt2Move Full Factorial Trial | GLOBAL ADVANCES IN INTEGRATIVE MEDICINE AND HEALTH | Different Study Design |  |  |  |  |
|  |  | 518 | Ream, E., et al. | 2020 | Telephone interventions for symptom management in adults with cancer | Cochrane Database of Systematic Reviews | Non Original Articles |  |  |  |  |
|  |  | 519 | Reblin, M. & Uchino, B. N. | 2008 | Social and emotional support and its implication for health | CURRENT OPINION IN PSYCHIATRY | Different Study Design |  |  |  |  |
|  |  | 520 | Reding, D. J., et al. | 1997 | Cancer screening and prevention in rural wisconsin: The Greater Marshfield Experience | Wisconsin medical journal | Different Study Design |  |  |  |  |
|  |  | 521 | Reese, J. B., et al. | 2022 | Investigating the impact of the COVID-19 pandemic on breast cancer clinicians' communication about sexual health | Supportive Care in Cancer | Duplicate |  |  |  |  |
|  |  | 522 | Reese, J. B., et al. | 2022 | Investigating the impact of the COVID-19 pandemic on breast cancer clinicians' communication about sexual health | Supportive Care in Cancer | Duplicate |  |  |  |  |
|  |  | 523 | Reese, J. B., et al. | 2022 | Investigating the impact of the COVID-19 pandemic on breast cancer clinicians' communication about sexual health | Supportive Care in Cancer | Different Study Design |  |  |  |  |
|  |  | 524 | Rees-Punia, E., et al. | 2022 | Pilot Randomized Controlled Trial of Feasibility, Acceptability, and Preliminary Efficacy of a Web-Based Physical Activity and Sedentary Time Intervention for Survivors of Physical Inactivity-Related Cancers | International Journal of Behavioral Medicine | Different Study Design |  |  |  |  |
|  |  | 525 | Rehman, A., et al. | 2024 | FedCSCD-GAN: A secure and collaborative framework for clinical cancer diagnosis via optimized federated learning and GAN | BIOMEDICAL SIGNAL PROCESSING AND CONTROL | Duplicate |  |  |  |  |
|  |  | 526 | Rehman, A., et al. | 2024 | FedCSCD-GAN: A secure and collaborative framework for clinical cancer diagnosis via optimized federated learning and GAN | BIOMEDICAL SIGNAL PROCESSING AND CONTROL | Non English |  |  |  |  |
|  |  | 527 | Reyes, BC | 2021 | Breast cancer caracteristics in young women | REVISTA DE SENOLOGIA Y PATOLOGIA MAMARIA | Different Study Design |  |  |  |  |
|  |  | 528 | Rezaee, R., et al. | 2022 | Development, usability and quality evaluation of the resilient mobile application for women with breast cancer | Health Science Reports | Duplicate |  |  |  |  |
|  |  | 529 | Rezaee, R., et al. | 2022 | Development, usability and quality evaluation of the resilient mobile application for women with breast cancer | Health Science Reports | Duplicate |  |  |  |  |
|  |  | 530 | Rezaee, R., et al. | 2022 | Development, usability and quality evaluation of the resilient mobile application for women with breast cancer | Health Science Reports | Different Study Design |  |  |  |  |
|  |  | 531 | Richardson, J. L., et al. | 1995 | Adherence to Screening Examinations for Colorectal Cancer After Diagnosis in a First-Degree Relative | Preventive Medicine | Duplicate |  |  |  |  |
|  |  | 532 | Richardson, J. L., et al. | 1995 | ADHERENCE TO SCREENING EXAMINATIONS FOR COLORECTAL-CANCER AFTER DIAGNOSIS IN A 1ST-DEGREE RELATIVE | Preventive Medicine | Different Study Design |  |  |  |  |
|  |  | 533 | Richardson-Parry, A., et al. | 2023 | Interventions to reduce cancer screening inequities: the perspective and role of patients, advocacy groups, and empowerment organizations | INTERNATIONAL JOURNAL FOR EQUITY IN HEALTH | Different Study Design |  |  |  |  |
|  |  | 534 | Rincon, E., Monteiro-Guerra, F., Rivera-Romero, O., et al. | 2017 | Mobile phone apps for quality of life and well-being assessment in breast and prostate cancer patients: Systematic review | JMIR mHealth and uHealth | Non Original Articles |  |  |  |  |
|  |  | 535 | Roberts, A. L., et al. | 2019 | Breast, Prostate, and Colorectal Cancer Survivors' Experiences of Using Publicly Available Physical Activity Mobile Apps: Qualitative Study | JMIR mHealth and uHealth | Different Study Design |  |  |  |  |
|  |  | 536 | Rochette, C., et al. | 2021 | Telephone follow-up of oncology patients: the contribution of the nurse specialist for a Service-Dominant Logic in hospital | BMC Health Services Research | Different Study Design |  |  |  |  |
|  |  | 537 | Rodriguez, G. M., et al. | 2021 | The Impact of COVID-19 on Patients With Cancer: A National Study of Patient Experiences | American Journal of Clinical Oncology | Different Study Design |  |  |  |  |
|  |  | 538 | Roh, S. & Lee, Y. S. | 2023 | Developing Culturally Tailored Mobile Web App Education to Promote Breast Cancer Screening: Knowledge, Barriers, and Needs Among American Indian Women | Journal of Cancer Education | Duplicate |  |  |  |  |
|  |  | 539 | Roh, S. & Lee, Y. S. | 2023 | Developing Culturally Tailored Mobile Web App Education to Promote Breast Cancer Screening: Knowledge, Barriers, and Needs Among American Indian Women | Journal of Cancer Education | Different Study Design |  |  |  |  |
|  |  | 540 | Roh, S., et al. | 2023 | Mobile Web App Intervention to Promote Breast Cancer Screening Among American Indian Women in the Northern Plains: Feasibility and Efficacy Study | JMIR Formative Research | Different Study Design |  |  |  |  |
|  |  | 541 | Ruco, A., Dossa, F., Tinmouth, J., et al. | 2020 | Social media and mobile health technology for cancer screening: A systematic review and meta-analysis protocol | BMJ Open | Non Original Articles |  |  |  |  |
|  |  | 542 | Ruland, C. M., et al. | 2013 | Evaluation of different features of an eHealth application for personalized illness management support: Cancer patients' use and appraisal of usefulness | International Journal of Medical Informatics | Duplicate |  |  |  |  |
|  |  | 543 | Ruland, C. M., et al. | 2013 | Evaluation of different features of an eHealth application for personalized illness management support: Cancer patients' use and appraisal of usefulness | International Journal of Medical Informatics | Different Study Design |  |  |  |  |
|  |  | 544 | Rutsch, M., et al. | 2020 | ReNaApp: increasing the long-term effects of oncological rehabilitation through an application after medical rehabilitation (ReNaApp): a quasi-randomized longitudinal study of prospective design | BMC Health Services Research | Different Study Design |  |  |  |  |
|  |  | 545 | Sabiston, C. M., et al. | 2023 | Exploring Peer Support Characteristics for Promoting Physical Activity Among Women Living Beyond a Cancer Diagnosis A Qualitative Descriptive Study | Oncology Nursing Forum | Different Study Design |  |  |  |  |
|  |  | 546 | Sado, M., et al. | 2022 | Effectiveness and Cost-effectiveness of Online Brief Mindfulness-based Cognitive Therapy for the Improvement of Productivity in the Workplace: Study Protocol for a Randomized Controlled Trial | JMIR Research Protocols | Different Study Design |  |  |  |  |
|  |  | 547 | Saevarsdottir, S. R. & Gudmundsdottir, S. L. | 2023 | Mobile Apps and Quality of Life in Patients With Breast Cancer and Survivors: Systematic Literature Review | Journal of Medical Internet Research | Non Original Articles |  |  |  |  |
|  |  | 548 | Safran, V., et al. | 2024 | Multilingual Framework for Risk Assessment and Symptom Tracking (MRAST) | Sensors | Different Study Design |  |  |  |  |
|  |  | 549 | Sahoo, S., et al. | 2020 | Problem-solving in technology-rich environments and cancer screening in later life | EUROPEAN JOURNAL OF CANCER PREVENTION | Different Study Design |  |  |  |  |
|  |  | 550 | Salinas, M. & Odedina, F. T. | 2024 | Addressing Breast Cancer Equity Through Virtual Community Oncology Navigation and Engagement (vCONET) | Cancer Control | Different Study Design |  |  |  |  |
|  |  | 551 | Salmani, H., et al. | 2022 | Smartphone-based application for self-management of patients with colorectal cancer: development and usability evaluation | Supportive Care in Cancer | Different Study Design |  |  |  |  |
|  |  | 552 | Salsman, J. M., et al. | 2023 | An eHealth, Positive Emotion Skills Intervention for Enhancing Psychological Well-Being in Young Adult Cancer Survivors: Results from a Multi-Site, Pilot Feasibility Trial | International Journal of Behavioral Medicine | Duplicate |  |  |  |  |
|  |  | 553 | Salsman, J. M., et al. | 2023 | An eHealth, Positive Emotion Skills Intervention for Enhancing Psychological Well-Being in Young Adult Cancer Survivors: Results from a Multi-Site, Pilot Feasibility Trial | International Journal of Behavioral Medicine | Duplicate |  |  |  |  |
|  |  | 554 | Salsman, J. M., et al. | 2023 | An eHealth, Positive Emotion Skills Intervention for Enhancing Psychological Well-Being in Young Adult Cancer Survivors: Results from a Multi-Site, Pilot Feasibility Trial | International Journal of Behavioral Medicine | Different Study Design |  |  |  |  |
|  |  | 555 | Samadbeik, M., et al. | 2023 | Mobile health interventions for cancer patient education: A scoping review | International Journal of Medical Informatics | Different Study Design |  |  |  |  |
|  |  | 556 | Sanders, A. B., et al. | 2019 | Physical Activity and Sedentary Behavior in Older Gastrointestinal Cancer Survivors: Need and Acceptability of Digital Health Interventions | JOURNAL OF GASTROINTESTINAL CANCER | Different Study Design |  |  |  |  |
|  |  | 557 | Sandre, A. R. & Newbold, K. B. | 2016 | Telemedicine: Bridging the Gap between Refugee Health and Health Services Accessibility in Hamilton, Ontario | REFUGE | Non Original Articles |  |  |  |  |
|  |  | 558 | Sani, S. B., et al. | 2021 | Comparison of the effectiveness of teaching breast cancer in a simple language via a user-friendly booklet or through WhatsApp on the learning rate and emotional status in healthy women: a randomized pre-test/post-test experimental design | International Journal of Health Promotion and Education | Different Study Design |  |  |  |  |
|  |  | 559 | Santiago-Torres, M., et al. | 2021 | ¡Mi Vida Saludable! A randomized, controlled, 2 × 2 factorial trial of a diet and physical activity intervention among Latina breast cancer survivors: Study design and methods | Contemporary Clinical Trials | Different Study Design |  |  |  |  |
|  |  | 560 | Santin, O., et al. | 2022 | Supporting someone with cancer during the COVID-19 pandemic: A mixed methods analysis of cancer carer's health, Quality of Life and need for support | HEALTH & SOCIAL CARE IN THE COMMUNITY | Different Study Design |  |  |  |  |
|  |  | 561 | Sauer, C., et al. | 2021 | eHealth intervention to manage symptoms for patients with cancer on immunotherapy (SOFIA): a study protocol for a randomised controlled external pilot trial | BMJ Open | Non English |  |  |  |  |
|  |  | 562 | Schwarz, PKN | 2022 | Telemedicine and navigation of patients with breast cancer. A study in four public hospitals in Argentina | ASTROLABIO-NUEVA EPOCA | Different Study Design |  |  |  |  |
|  |  | 563 | Schlecht, S., et al. | 2023 | Changes of symptoms of anxiety, depression, and fatigue in cancer patients 3 months after a video-based intervention | International Journal of Environmental Research and Public Health | Different Study Design |  |  |  |  |
|  |  | 564 | Sefenu, R. P. S., et al. | 2024 | Development of a Culturally Appropriate Text Messaging Platform for Improving Breast Cancer Screening Uptake Among Ghanaian Women in Metropolitan Areas | INTERNATIONAL JOURNAL OF BREAST CANCER | Duplicate |  |  |  |  |
|  |  | 565 | Sefenu, R. P. S., et al. | 2024 | Development of a Culturally Appropriate Text Messaging Platform for Improving Breast Cancer Screening Uptake Among Ghanaian Women in Metropolitan Areas | INTERNATIONAL JOURNAL OF BREAST CANCER | Different Study Design |  |  |  |  |
|  |  | 566 | Seib, C., et al. | 2022 | Improving health-related quality of life in women with breast, blood, and gynaecological Cancer with an eHealth-enabled 12-week lifestyle intervention: the women's wellness after Cancer program randomised controlled trial | BMC Cancer | Different Study Design |  |  |  |  |
|  |  | 567 | Sekar, P., et al. | 2022 | The dynamics of breast cancer screening approaches in urban India: An ethnographic study from Delhi | SSM-QUALITATIVE RESEARCH IN HEALTH | Different Study Design |  |  |  |  |
|  |  | 568 | Sella, T., et al. | 2021 | Young, Empowered and Strong: A Web-Based Education and Supportive Care Intervention for Young Women With Breast Cancer Across the Care Continuum | JCO CLINICAL CANCER INFORMATICS | Different Study Design |  |  |  |  |
|  |  | 569 | Serra-Blasco, M., et al. | 2024 | Cognitive-enhanced eHealth psychosocial stepped intervention for managing breast cancer-related cognitive impairment: Protocol for a randomized controlled trial | Digital Health | Different Study Design |  |  |  |  |
|  |  | 570 | Seven, M., et al. | 2022 | A Mobile Application for Symptom Management in Patients With Breast Cancer | Oncology Nursing Forum | Different Study Design |  |  |  |  |
|  |  | 571 | Shah, Y. B., et al. | 2023 | Risk factors for heightened COVID-19-Related anxiety among breast cancer patients | Cancer Medicine | Different Study Design |  |  |  |  |
|  |  | 572 | Shah, Y. B., et al. | 2023 | Risk factors for heightened COVID-19-Related anxiety among breast cancer patients | Cancer Medicine | Different Study Design |  |  |  |  |
|  |  | 573 | Shakery, M., et al. | 2021 | The effect of a smartphone application on women's performance and health beliefs about breast self-examination: a quasi-experimental study | BMC Medical Informatics and Decision Making | Different Study Design |  |  |  |  |
|  |  | 574 | Shaw, B., et al. | 2006 | How underserved breast cancer patients use and benefit from eHealth programs - Implications for closing the digital divide | American Behavioral Scientist | Duplicate |  |  |  |  |
|  |  | 575 | Shaw, B., et al. | 2006 | How underserved breast cancer patients use and benefit from eHealth programs: Implications for closing the digital divide | American Behavioral Scientist | Different Study Design |  |  |  |  |
|  |  | 576 | Shaw, B. R., et al. | 2008 | Antecedent characteristics of online cancer information seeking among rural breast cancer patients: An application of the Cognitive-Social Health Information Processing (C-SHIP) model | JOURNAL OF HEALTH COMMUNICATION | Different Study Design |  |  |  |  |
|  |  | 577 | Shen, S., et al. | 2024 | Evaluation of a mobile behavior change program for weight loss in breast cancer survivors | NPJ BREAST CANCER | Different Study Design |  |  |  |  |
|  |  | 578 | Sherman, D. W., et al. | 2012 | The effects of psychoeducation and telephone counseling on the adjustment of women with early-stage breast cancer | Applied Nursing Research | Different Study Design |  |  |  |  |
|  |  | 579 | Shi, N., et al. | 2024 | Feasibility of a mobile health app-based self-management program for Chinese patients with breast cancer receiving chemotherapy: A randomized controlled pilot study | Digital Health | Duplicate |  |  |  |  |
|  |  | 580 | Shi, N., et al. | 2024 | Feasibility of a mobile health app-based self-management program for Chinese patients with breast cancer receiving chemotherapy: A randomized controlled pilot study | Digital Health | Duplicate |  |  |  |  |
|  |  | 581 | Shi, N., et al. | 2024 | Feasibility of a mobile health app-based self-management program for Chinese patients with breast cancer receiving chemotherapy: A randomized controlled pilot study | Digital Health | Different Study Design |  |  |  |  |
|  |  | 582 | Shih, Y. W., et al. | 2020 | The Association Between Smartphone Use and Breast Cancer Risk Among Taiwanese Women: A Case-Control Study | CANCER MANAGEMENT AND RESEARCH | Different Study Design |  |  |  |  |
|  |  | 583 | Shin, E. & Shim, J. M. | 2019 | Listen to Doctors, Friends, or Both? Embedded They Produce Thick Knowledge and Promote Health | JOURNAL OF HEALTH COMMUNICATION | Different Study Design |  |  |  |  |
|  |  | 584 | Short, C. E., et al. | 2024 | Evaluating a Remotely Delivered Cardio-Oncology Rehabilitation Intervention for Patients With Breast Cancer (REMOTE-COR-B): Protocol for a Single-Arm Feasibility Trial | JMIR Research Protocols | Different Study Design |  |  |  |  |
|  |  | 585 | Short, C. E., et al. | 2017 | How do different delivery schedules of tailored web-based physical activity advice for breast cancer survivors influence intervention use and efficacy? | Journal of Cancer Survivorship | Different Study Design |  |  |  |  |
|  |  | 586 | Shtaynberger, J. & Krebs, P. | 2016 | Associations Between Decisional Balance and Health Behaviors Among Adult Cancer Survivors | Journal of Cancer Education | Duplicate |  |  |  |  |
|  |  | 587 | Shtaynberger, J. & Krebs, P. | 2016 | Associations Between Decisional Balance and Health Behaviors Among Adult Cancer Survivors | Journal of Cancer Education | Different Study Design |  |  |  |  |
|  |  | 588 | Signorelli, G. R., et al. | 2019 | A Research Roadmap: Connected Health as an Enabler of Cancer Patient Support | Journal of Medical Internet Research | Different Study Design |  |  |  |  |
|  |  | 589 | Signorelli, G. R., et al. | 2022 | Breast Cancer Physical Activity Mobile Intervention: Early Findings from a User Experience and Acceptability Mixed Methods Study | JMIR Formative Research | Duplicate |  |  |  |  |
|  |  | 590 | Signorelli, G. R., et al. | 2022 | Breast Cancer Physical Activity Mobile Intervention: Early Findings From a User Experience and Acceptability Mixed Methods Study | JMIR Formative Research | Different Study Design |  |  |  |  |
|  |  | 591 | Sinclair, J. M. A., et al. | 2020 | A Context-Specific Digital Alcohol Brief Intervention in Symptomatic Breast Clinics (Abreast of Health): Development and Usability Study | JMIR Research Protocols | Different Study Design |  |  |  |  |
|  |  | 592 | Singleton, A., et al. | 2019 | A text message intervention to support women's physical and mental health after breast cancer treatments (EMPOWER-SMS): a randomised controlled trial protocol | BMC Cancer | Different Study Design |  |  |  |  |
|  |  | 593 | Singleton, A., et al. | 2021 | Co-designing a Lifestyle-Focused Text Message Intervention for Women After Breast Cancer Treatment: Mixed Methods Study | Journal of Medical Internet Research | Different Study Design |  |  |  |  |
|  |  | 594 | Singleton, A. C., et al. | 2023 | Supporting women's health outcomes after breast cancer treatment comparing a text message intervention to usual care: the EMPOWER-SMS randomised clinical trial | Journal of Cancer Survivorship | Duplicate |  |  |  |  |
|  |  | 595 | Singleton, A. C., et al. | 2023 | Supporting women's health outcomes after breast cancer treatment comparing a text message intervention to usual care: the EMPOWER-SMS randomised clinical trial | Journal of Cancer Survivorship | Different Study Design |  |  |  |  |
|  |  | 596 | Singleton, A. C., et al. | 2022 | Supporting breast cancer survivors via text messages: reach, acceptability, and utility of EMPOWER-SMS | Journal of Cancer Survivorship | Non English |  |  |  |  |
|  |  | 597 | Singleton, A.C., Raeside, R., Hyun, K.K., et al. | 2022 | Electronic Health Interventions for Patients with Breast Cancer: Systematic Review and Meta-Analyses | Journal of Clinical Oncology | Non Original Articles |  |  |  |  |
|  |  | 598 | Singer, S. et al. | 2024 | Digital health applications in psycho-oncology: Claims and reality | Onkologie | Different Study Design |  |  |  |  |
|  |  | 599 | Sinha, N. & Sharma, A. | 2024 | Digital media intervention for breast cancer awareness among rural women: A quasi-experimental study from Bihar, India | CLINICAL EPIDEMIOLOGY AND GLOBAL HEALTH | Duplicate |  |  |  |  |
|  |  | 600 | Sinha, N. & Sharma, A. | 2024 | Digital media intervention for breast cancer awareness among rural women: A quasi-experimental study from Bihar, India | CLINICAL EPIDEMIOLOGY AND GLOBAL HEALTH | Different Study Design |  |  |  |  |
|  |  | 601 | Skiba, M. B., et al. | 2022 | Health Promotion Among Mexican-Origin Survivors of Breast Cancer and Caregivers Living in the United States-Mexico Border Region: Qualitative Analysis From the Vida Plena Study | JMIR Cancer | Duplicate |  |  |  |  |
|  |  | 602 | Skiba, M. B., et al. | 2022 | Health Promotion Among Mexican-Origin Survivors of Breast Cancer and Caregivers Living in the United States-Mexico Border Region: Qualitative Analysis From the Vida Plena Study | JMIR Cancer | Different Study Design |  |  |  |  |
|  |  | 603 | Smetherman, D. H. | 2021 | Breast Cancer Screening and the COVID-19 Pandemic | JOURNAL OF BREAST IMAGING | Different Study Design |  |  |  |  |
|  |  | 604 | Smith, A., et al. | 2020 | Development and usability evaluation of an online self-management intervention for fear of cancer recurrence (iConquerFear) | PSYCHO-ONCOLOGY | Non Original Articles |  |  |  |  |
|  |  | 605 | Smith, G. V. H., et al. | 2024 | Virtually Supervised Exercise Programs for People With Cancer | CANCER NURSING | Non Original Articles |  |  |  |  |
|  |  | 606 | Smith, J., et al. | 2024 | Experiences of using a supported digital intervention for cancer survivors in primary care: a qualitative process evaluation | Journal of Cancer Survivorship | Different Study Design |  |  |  |  |
|  |  | 607 | Snyder, M. & Elkins, G. R. | 2024 | Characteristics of Users of a Digital Hypnotherapy Intervention for Hot Flashes: Retrospective Study | JMIR Formative Research | Duplicate |  |  |  |  |
|  |  | 608 | Snyder, M. & Elkins, G. R. | 2024 | Characteristics of Users of a Digital Hypnotherapy Intervention for Hot Flashes: Retrospective Study | JMIR Formative Research | Different Study Design |  |  |  |  |
|  |  | 609 | Sohrabi, S., Atashi, A. | 2021 | The impact of mobile health on breast cancer patient's life and treatment: A systematic review | Frontiers in Health Informatics | Non Original Articles |  |  |  |  |
|  |  | 610 | Song, L. X., et al. | 2018 | Enhancing Survivorship Care Planning for Patients With Localized Prostate Cancer Using a Couple-Focused mHealth Symptom Self-Management Program: Protocol for a Feasibility Study | JMIR Research Protocols | Different Study Design |  |  |  |  |
|  |  | 611 | Song, L. X., et al. | 2022 | Testing the efficacy of a couple-focused, tailored eHealth intervention for symptom self-management among men with prostate cancer and their partners: the study protocol | Trials | Different Study Design |  |  |  |  |
|  |  | 612 | Sorice-Virk, S., et al. | 2024 | Patient Perceptions of Cancer and Reconstructive Care During the COVID-19 Pandemic | Plastic Surgery | Different Study Design |  |  |  |  |
|  |  | 613 | Sotirova, M.B., McCaughan, E.M., Ramsey, L., et al. | 2021 | Acceptability of online exercise-based interventions after breast cancer surgery: systematic review and narrative synthesis | Journal of Cancer Survivorship | Non Original Articles |  |  |  |  |
|  |  | 614 | Spark, L. C., et al. | 2015 | Efficacy of a Text Message-Delivered Extended Contact Intervention on Maintenance of Weight Loss, Physical Activity, and Dietary Behavior Change | JMIR mHealth and uHealth | Different Study Design |  |  |  |  |
|  |  | 615 | Sr, D. H., et al. | 2015 | The effect of an information and communication technology (ICT) on older adults' quality of life: study protocol for a randomized control trial | Trials | Different Study Design |  |  |  |  |
|  |  | 616 | Sr, D. H. G., et al. | 2022 | Using Smart Displays to Implement an eHealth System for Older Adults With Multiple Chronic Conditions: Protocol for a Randomized Controlled Trial | JMIR Research Protocols | Different Study Design |  |  |  |  |
|  |  | 617 | St George, S. M., et al. | 2020 | Development of a multigenerational digital lifestyle intervention for women cancer survivors and their families | PSYCHO-ONCOLOGY | Different Study Design |  |  |  |  |
|  |  | 618 | Stinesen-Kollberg, K., et al. | 2013 | Worry about one's own children, psychological well-being, and interest in psychosocial intervention | PSYCHO-ONCOLOGY | Different Study Design |  |  |  |  |
|  |  | 619 | Street, R. L., et al. | 2022 | Managing uncertainty and responding to difficult emotions: Cancer patients? perspectives on clinician response during the COVID-19 pandemic | Patient Education and Counseling | Different Study Design |  |  |  |  |
|  |  | 620 | Street, R. L., et al. | 2000 | Specialist-primary care provider-patient communication in telemedical consultations | TELEMEDICINE JOURNAL | Different Study Design |  |  |  |  |
|  |  | 621 | Stubbins, R., et al. | 2018 | A behavior-modification, clinical-grade mobile application to improve breast cancer survivors' accountability and health outcomes | JCO CLINICAL CANCER INFORMATICS | Duplicate |  |  |  |  |
|  |  | 622 | Stubbins, R., et al. | 2018 | A Behavior-Modification, Clinical-Grade Mobile Application to Improve Breast Cancer Survivors' Accountability and Health Outcomes | JCO CLINICAL CANCER INFORMATICS | Different Study Design |  |  |  |  |
|  |  | 623 | Subramanian, S., et al. | 2021 | Acceptability, Utility, and Cost of a Mobile Health Cancer Screening Education Application for Training Primary Care Physicians in India | Oncologist | Different Study Design |  |  |  |  |
|  |  | 624 | Subramanian, S., et al. | 2021 | Acceptability, Utility, and Cost of a Mobile Health Cancer Screening Education Application for Training Primary Care Physicians in India | Oncologist | Different Study Design |  |  |  |  |
|  |  | 625 | Svetlák, M., et al. | 2023 | The effectiveness of three mobile-based psychological interventions in reducing psychological distress and preventing stress-related changes in the psycho-neuro-endocrine-immune network in breast cancer survivors: Study protocol for a randomised controlled trial | INTERNET INTERVENTIONS-THE APPLICATION OF INFORMATION TECHNOLOGY IN MENTAL AND BEHAVIOURAL HEALTH | Duplicate |  |  |  |  |
|  |  | 626 | Světlák, M., et al. | 2023 | The effectiveness of three mobile-based psychological interventions in reducing psychological distress and preventing stress-related changes in the psycho-neuro-endocrine-immune network in breast cancer survivors: Study protocol for a randomised controlled trial | INTERNET INTERVENTIONS-THE APPLICATION OF INFORMATION TECHNOLOGY IN MENTAL AND BEHAVIOURAL HEALTH | Different Study Design |  |  |  |  |
|  |  | 627 | Swartz, M. C., et al. | 2023 | Assessing the Suitability of a Virtual 'Pink Warrior' for Older Breast Cancer Survivors during COVID-19: A Pilot Study | LIFE-BASEL | Different Study Design |  |  |  |  |
|  |  | 628 | Tan, J. Y. A., Ong, G. Y. Q., Cheng, L. J., Pikkarainen, M. & He, H. G | 2023 | Effectiveness of mHealth-based psychosocial interventions for breast cancer patients and their caregivers: A systematic review and meta-analysis | Journal of Telemedicine & Telecare | Duplicate |  |  |  |  |
|  |  | 629 | Tan, J. Y. A., Ong, G. Y. Q., Cheng, L. J., Pikkarainen, M. & He, H. G | 2023 | Effectiveness of mHealth-based psychosocial interventions for breast cancer patients and their caregivers: A systematic review and meta-analysis | Journal of Telemedicine & Telecare | Non Original Articles |  |  |  |  |
|  |  | 630 | Tandon, A., Dhir, A. & Islam, N | 2023 | Mobile Health Interventions for Cancer Care and Support: The Next Level of Digitalization in Healthcare? | IEEE Transactions on Engineering Management | Non Original Articles |  |  |  |  |
|  |  | 631 | Taramasco, C., Rimassa, C., Noel, R., Storm, M. L. B. & Sánchez, C | 2023 | Co-design of a Mobile App for Engaging Breast Cancer Patients in Reporting Health Experiences: Qualitative Case Study | Journal of Medical Internet Research | Non Original Articles |  |  |  |  |
|  |  | 632 | Taylor, K. L., Diplacido, J., Redd, W. H., Faccenda, K., Greer, L. & Perlmutter, A | 1999 | Demographics, family histories, and psychological characteristics of prostate carcinoma screening participants | Journal of the American Cancer Society | Different Study Design |  |  |  |  |
|  |  | 633 | Thiessen, M., Sinclair, S., Tang, P. A. & Bouchal, S. R | 2020 | Information Access and Use by Patients With Cancer and Their Friends and Family: Development of a Grounded Theory | Journal of Medical Internet Research | Different Study Design |  |  |  |  |
|  |  | 634 | Tokgöz, P., Dockweiler, C. | 2022 | Telemedicine in Rehabilitation Aftercare for Women with Breast Cancer - A Systematic Literature Review | Rehabilitation | Non Original Articles |  |  |  |  |
|  |  | 635 | Toohey, K., Paterson, C., Moore, M. & Hunter, M | 2022 | Towards best practice in the delivery of prescribed exercise via telehealth for individuals diagnosed with cancer: A randomised controlled trial protocol | Contemporary Clinical Trials | Different Study Design |  |  |  |  |
|  |  | 636 | Torrez, C. R. Z., Gasior, J. A., Ginzberg, S. P., Nunes, L. W., Fayanju, O. M., Englander, B. S., Elmore, L. C. & Edmonds, C. E | 2024 | Identifying and Addressing Barriers to Screening Mammography in a Medically Underserved Community | ACADEMIC RADIOLOGY | Different Study Design |  |  |  |  |
|  |  | 637 | Trabjerg, T. B., Jensen, L. H., Sondergaard, J., Sisler, J. J. & Hansen, D. G | 2019 | Improving continuity by bringing the cancer patient, general practitioner and oncologist together in a shared video-based consultation - protocol for a randomised controlled trial | BMC FAMILY PRACTICE | Different Study Design |  |  |  |  |
|  |  | 638 | Triantafyllidis, A., Kondylakis, H., Katehakis, D., Kouroubali, A., Alexiadis, A., Segkouli, S., Votis, K. & Tzovaras, D | 2024 | Smartwatch interventions in healthcare: A systematic review of the literature | International Journal of Medical Informatics | Non Original Articles |  |  |  |  |
|  |  | 639 | Tshabalala, G., Blanchard, C., Mmoledi, K., Malope, D., O’neil, D. S., Norris, S. A., Joffe, M. & Dietrich, J. J | 2023 | A qualitative study to explore healthcare providers’ perspectives on barriers and enablers to early detection of breast and cervical cancers among women attending primary healthcare clinics in Johannesburg, South Africa | PLOS Global Public Health | Different Study Design |  |  |  |  |
|  |  | 640 | Tuckey, N., Iasiello, M., Corsini, N., Koczwara, B., Bareham, M., Wellalagodage, A. & Wardill, H. R | 2024 | 'Just Google it'-A scoping review of online mental health resources for survivors of breast cancer | PSYCHO-ONCOLOGY | Different Study Design |  |  |  |  |
|  |  | 641 | Tumeh, I., Bergerot, C. D., Lee, D., Philip, E. J. & Freitas, R., Jr | 2023 | mHealth program for patients with advanced cancer receiving treatment in a public health hospital in Brazil | PSYCHO-ONCOLOGY | Duplicate |  |  |  |  |
|  |  | 642 | Tumeh, I., Bergerot, C. D., Lee, D., Philip, E. J. & Freitas, R., Jr | 2023 | mHealth program for patients with advanced cancer receiving treatment in a public health hospital in Brazil | PSYCHO-ONCOLOGY | Different Study Design |  |  |  |  |
|  |  | 643 | Turnbull, D., Irwig, L., Simpson, J. M., Donnelly, N. & Mock, P | 1995 | A PROSPECTIVE COHORT STUDY INVESTIGATING PSYCHOSOCIAL PREDICTORS OF ATTENDANCE AT A MOBILE BREAST SCREENING SERVICE | AUSTRALIAN JOURNAL OF PUBLIC HEALTH | Non English |  |  |  |  |
|  |  | 644 | Tokgoz, P., Dockweiler, C. | 2023 | Telemedicine in Rehabilitation Aftercare for Women with Breast Cancer - A Systematic Literature Review | Rehabilitation (Germany) | Non English |  |  |  |  |
|  |  | 645 | Tokgoz, P., Dockweiler, C. | 2023 | Telemedicine in Rehabilitation Aftercare for Women with Breast Cancer - A Systematic Literature Review | Tumor Diagnostik und Therapie | Different Study Design |  |  |  |  |
|  |  | 646 | Tollefson, M., Eriksen, N., Pathak, N. | 2021 | Improving Women's Health Across the Lifespan | Improving Women's Health Across the Lifespan | Different Study Design |  |  |  |  |
|  |  | 647 | Ueland, K., et al. | 2022 | A digital health intervention to improve nutrition and physical activity in breast cancer survivors: Rationale and design of the Cook and Move for Your Life pilot and feasibility randomized controlled trial | Contemporary Clinical Trials | Different Study Design |  |  |  |  |
|  |  | 648 | Vallance, J. K., et al | 2020 | Effects of the ACTIVity And TEchnology (ACTIVATE) intervention on health-related quality of life and fatigue outcomes in breast cancer survivors | PSYCHO-ONCOLOGY | Different Study Design |  |  |  |  |
|  |  | 649 | Vaccarella, M., Myers, K.R. | 2021 | Medical Humanities: Criticism and Creativity Breast Cancer Inside Out: Bodies, Biographies & Beliefs | Medical Humanities: Criticism and Creativity Breast Cancer Inside Out: Bodies, Biographies & Beliefs | Non Original Articles |  |  |  |  |
|  |  | 650 | Valle, C. G., et al | 2023 | Effect of an mHealth intervention on physical activity outcomes among young adult cancer survivors: The IMPACT randomized controlled trial | Cancer | Different Study Design |  |  |  |  |
|  |  | 651 | Van Beek, F. E., Wijnhoven, L. M. A., Jansen, F., Custers, J. a. E., Aukema, E. J., Coupé, V. M. H., Cuijpers, P., Van Der Lee, M. L., Lissenberg-Witte, B., Wijnen, B., Prins, J. B. & Verdonck-De Leeuw, I. M | 2019 | Prevalence of adjustment disorder among cancer patients, and the reach, effectiveness, cost-utility and budget impact of tailored psychological treatment: study protocol of a randomized controlled trial | BMC PSYCHOLOGY | Different Study Design |  |  |  |  |
|  |  | 652 | Van Den Berg, S. W., Gielissen, M. F. M., Ottevanger, P. B. & Prins, J. B | 2012 | Rationale of the BREAst cancer e-healTH [BREATH] multicentre randomised controlled trial: An Internet-based self-management intervention to foster adjustment after curative breast cancer by decreasing distress and increasing empowerment | BMC Cancer | Different Study Design |  |  |  |  |
|  |  | 653 | Van Der Hout, A., et al. | 2021 | The eHealth self-management application 'Oncokompas' that supports cancer survivors to improve health-related quality of life and reduce symptoms: which groups benefit most? | Acta Oncologica | Different Study Design |  |  |  |  |
|  |  | 654 | Van Der Hout, A., et al. | 2021 | The eHealth self-management application ‘Oncokompas’ that supports cancer survivors to improve health-related quality of life and reduce symptoms: which groups benefit most? : | Acta Oncologica | Different Study Design |  |  |  |  |
|  |  | 655 | Van Der Hout, A., et al. | 2017 | Efficacy, cost-utility and reach of an eHealth self-management application 'Oncokompas' that helps cancer survivors to obtain optimal supportive care: Study protocol for a randomised controlled trial | Trials | Different Study Design |  |  |  |  |
|  |  | 656 | Van Der Hout, A., et al. | 2017 | Efficacy, cost-utility and reach of an eHealth self-management application 'Oncokompas' that helps cancer survivors to obtain optimal supportive care: study protocol for a randomised controlled trial | Trials | Different Study Design |  |  |  |  |
|  |  | 657 | Van Drongelen, A., Boot, C. R. L., Hlobil, H., Twisk, J. W. R., Smid, T. & Van Der Beek, A. J | 2014 | Evaluation of an mHealth intervention aiming to improve health-related behavior and sleep and reduce fatigue among airline pilots | SCANDINAVIAN JOURNAL OF WORK ENVIRONMENT & HEALTH | Different Study Design |  |  |  |  |
|  |  | 658 | Van Eenbergen, M. et al | 2022 | Comparing Survivors of Cancer in Population-Based Samples With Those in Online Cancer Communities: Cross-sectional Questionnaire Study | JMIR Cancer | Different Study Design |  |  |  |  |
|  |  | 659 | Van Helmondt, S. J., Van Der Lee, M. L., Van Woezik, R. a. M., Lodder, P. & De Vries, J | 2020 | No effect of CBT-based online self-help training to reduce fear of cancer recurrence: First results of the CAREST multicenter randomized controlled trial | PSYCHO-ONCOLOGY | Different Study Design |  |  |  |  |
|  |  | 660 | Van Ravesteyn, N. et al | 2015 | Transition From Film to Digital Mammography Impact for Breast Cancer Screening Through the National Breast and Cervical Cancer Early Detection Program | American Journal of Preventive Medicine | Duplicate |  |  |  |  |
|  |  | 661 | Van Ravesteyn, N. T., Van Lier, L., Schechter, C. B., Ekwueme, D. U., Royalty, J., Miller, J. W., Near, A. M., Cronin, K. A., Heijnsdijk, E. a. M., Mandelblatt, J. S. & De Koning, H. J | 2015 | Transition from film to digital mammography: Impact for breast cancer screening through the national breast and cervical cancer early detection program | American Journal of Preventive Medicine | Different Study Design |  |  |  |  |
|  |  | 662 | Ventura, F., Koinberg, I., Karlsson, P., Sawatzky, R. & Öhlén, J | 2016 | Purposeful Agency in Support Seeking During Cancer Treatment From a Person-Centered Perspective | Global Qualitative Nursing Research | Duplicate |  |  |  |  |
|  |  | 663 | Ventura, F., Koinberg, I., Karlsson, P., Sawatzky, R. & Öhlén, J | 2016 | Purposeful Agency in Support Seeking During Cancer Treatment From a Person-Centered Perspective | Global Qualitative Nursing Research | Different Study Design |  |  |  |  |
|  |  | 664 | Ventura, F., Sawatzky, R., Ohlen, J., Karlsson, P. & Koinberg, I | 2017 | Challenges of evaluating a computer-based educational programme for women diagnosed with early-stage breast cancer: a randomised controlled trial | European Journal of Cancer Care | Duplicate |  |  |  |  |
|  |  | 665 | Ventura, F., Sawatzky, R., Öhlén, J., Karlsson, P. & Koinberg, I | 2017 | Challenges of evaluating a computer-based educational programme for women diagnosed with early-stage breast cancer: a randomised controlled trial | European Journal of Cancer Care | Duplicate |  |  |  |  |
|  |  | 666 | Ventura, F., Sawatzky, R., Öhlén, J., Karlsson, P. & Koinberg, I | 2017 | Challenges of evaluating a computer-based educational programme for women diagnosed with early-stage breast cancer: a randomised controlled trial | European Journal of Cancer Care | Included |  |  |  |  |
|  |  | 667 | Villani, D., Cognetta, C., Repetto, C., Serino, S., Toniolo, D., Scanzi, F. & Riva, G | 2018 | Promoting Emotional Well-Being in Older Breast Cancer Patients: Results From an eHealth Intervention | Frontiers in Psychology | Duplicate |  |  |  |  |
|  |  | 668 | Villani, D., Cognetta, C., Repetto, C., Serino, S., Toniolo, D., Scanzi, F. & Riva, G | 2018 | Promoting emotional well-being in older breast cancer patients: Results from an eHealth intervention | Frontiers in Psychology | Different Study Design |  |  |  |  |
|  |  | 669 | Villani, D., Cognetta, C., Repetto, C., Serino, S., Toniolo, D., Scanzi, F. & Riva, G | 2018 | Promoting Emotional Well-Being in Older Breast Cancer Patients: Results From an eHealth Intervention | Frontiers in Psychology | Different Study Design |  |  |  |  |
|  |  | 670 | Vogel, R. I., Niendorf, K., Petzel, S., Lee, H., Teoh, D., Blaes, A. H., Argenta, P., Rivard, C., Winterhoff, B., Lee, H. Y. & Geller, M. A | 2019 | A patient-centered mobile health application to motivate use of genetic counseling among women with ovarian cancer: A pilot randomized controlled trial | GYNECOLOGIC ONCOLOGY | Different Study Design |  |  |  |  |
|  |  | 671 | Wagner, L. I., Tooze, J. A., Hall, D. L., Levine, B. J., Beaumont, J., Duffecy, J., Victorson, D., Gradishar, W., Leach, J., Saphner, T., Sturtz, K., Smith, M. L., Penedo, F. & Cella, D | 2021 | Targeted eHealth Intervention to Reduce Breast Cancer Survivors' Fear of Recurrence: Results From the FoRtitude Randomized Trial | JNCI-JOURNAL OF THE NATIONAL CANCER INSTITUTE | Duplicate |  |  |  |  |
|  |  | 672 | Wagner, L. I., Tooze, J. A., Hall, D. L., Levine, B. J., Beaumont, J., Duffecy, J., Victorson, D., Gradishar, W., Leach, J., Saphner, T., Sturtz, K., Smith, M. L., Penedo, F., Mohr, D. C. & Cella, D | 2021 | Targeted eHealth Intervention to Reduce Breast Cancer Survivors' Fear of Recurrence: Results From the FoRtitude Randomized Trial | JNCI-JOURNAL OF THE NATIONAL CANCER INSTITUTE | Different Study Design |  |  |  |  |
|  |  | 673 | Walsh, J. C., Richmond, J., Mc Sharry, J., Groarke, A., Glynn, L., Kelly, M. G., Harney, O. & Groarke, J. M | 2021 | Examining the Impact of an mHealth Behavior Change Intervention With a Brief In-Person Component for Cancer Survivors With Overweight or Obesity: Randomized Controlled Trial | JMIR mHealth and uHealth | Different Study Design |  |  |  |  |
|  |  | 674 | Watanabe-Galloway, S., Ratnapradipa, K., Subramanian, R., Ramos, A., Famojuro, O., Schmidt, C. & Farazi, P | 2023 | Mobile Health (mHealth) Interventions to Increase Cancer Screening Rates in Hispanic/Latinx Populations: A Scoping Review | Health promotion practice | Different Study Design |  |  |  |  |
|  |  | 675 | Wee, L. E., Koh, G. C. H., Auyong, L. S., Cheong, A., Myo, T. T., Lin, J., Lim, E., Tan, S., Sundaramurthy, S., Koh, C. W., Ramakrishnan, P., Aariyapillai-Rajagopal, R., Vaidynathan-Selvamuthu, H. & Ma-Ma, K | 2014 | Screening for cardiovascular disease risk factors at baseline and post intervention among adults with intellectual disabilities in an urbanised Asian society | JOURNAL OF INTELLECTUAL DISABILITY RESEARCH | Different Study Design |  |  |  |  |
|  |  | 676 | Weiner, L. S., Nagel, S., Su, H. I., Hurst, S. & Hartman, S. J | 2020 | A Remotely Delivered, Peer-Led Physical Activity Intervention for Younger Breast Cancer Survivors (Pink Body Spirit): Protocol for a Feasibility Study and Mixed Methods Process Evaluation | JMIR Research Protocols | Different Study Design |  |  |  |  |
|  |  | 677 | Weng, L. Z., Lin, W. L., Lin, X. X., Liu, M. B. & Yang, J | 2024 | Randomized controlled trial of an app for cancer pain management | Supportive Care in Cancer | Different Study Design |  |  |  |  |
|  |  | 678 | Westerlinck, P., Coucke, P. & Albert, A | 2024 | Development of a cancer risk model and mobile health application to inform the public about cancer risks and risk factors | International Journal of Medical Informatics | Different Study Design |  |  |  |  |
|  |  | 679 | White-Means, S., Dapremont, J., Davis, B. D. & Thompson, T | 2020 | Who can help us on this journey? African american woman with breast cancer: Living in a city with extreme health disparities | International Journal of Environmental Research and Public Health | Duplicate |  |  |  |  |
|  |  | 680 | White-Means, S., Dapremont, J., Davis, B. D. & Thompson, T | 2020 | Who Can Help Us on This Journey? African American Woman with Breast Cancer: Living in a City with Extreme Health Disparities | International Journal of Environmental Research and Public Health | Different Study Design |  |  |  |  |
|  |  | 681 | Williamson, S., Beaver, K. & Langton, S | 2020 | Exploring health care professionals views on alternative approaches to cancer follow-up and barriers and facilitators to implementation of a recovery package | EUROPEAN JOURNAL OF ONCOLOGY NURSING | Non Original Articles |  |  |  |  |
|  |  | 682 | Winefield, H. R., Coventry, B. J. & Lambert, V | 2004 | Setting up a health education website: practical advice for health professionals | Patient Education and Counseling | Different Study Design |  |  |  |  |
|  |  | 683 | Wise, M., Han, J. Y., Shaw, B., Mctavish, F. & Gustafson, D. H | 2008 | Effects of using online narrative and didactic information on healthcare participation for breast cancer patients | Patient Education and Counseling | Different Study Design |  |  |  |  |
|  |  | 684 | Wojczulis, T., Ziemek, K., Zukowska, E., Janota, A. & Dzierzanowski, T | 2024 | The use of mindfulness practices in alleviating mental disorders in patients under palliative care | MEDYCYNA PALIATYWNA-PALLIATIVE MEDICINE | Included |  |  |  |  |
|  |  | 685 | Wolff, J., Wuelfing, P., Koenig, A., Ehrl, B., Damsch, J., Smollich, M., Baumann, F. T., Harbeck, N. & Wuerstlein, R | 2023 | App-based lifestyle coaching (PINK!) accompanying breast cancer patients and survivors to reduce psychological distress and fatigue and improve physical activity - A feasibility pilot study | Breast Care | Duplicate |  |  |  |  |
|  |  | 686 | Wolff, J., Wuelfing, P., König, A., Ehrl, B., Damsch, J., Smollich, M., Baumann, F. T., Harbeck, N. & Wuerstlein, R | 2023 | App-Based Lifestyle Coaching (PINK!) Accompanying Breast Cancer Patients and Survivors to Reduce Psychological Distress and Fatigue and Improve Physical Activity: A Feasibility Pilot Study | Breast Care | Different Study Design |  |  |  |  |
|  |  | 687 | Wolvers, M. D. J., Bruggeman-Everts, F. Z., Van Der Lee, M. L., Van De Schoot, R. & Vollenbroek-Hutten, M. M. R | 2015 | Effectiveness, Mediators, and Effect Predictors of Internet Interventions for Chronic Cancer-Related Fatigue: The Design and an Analysis Plan of a 3-Armed Randomized Controlled Trial | JMIR Research Protocols | Different Study Design |  |  |  |  |
|  |  | 688 | Wu, F. N., Rotimi, O., Laza-Cagigas, R. & Rampal, T | 2021 | The Feasibility and Effects of a Telehealth-Delivered Home-Based Prehabilitation Program for Cancer Patients during the Pandemic | CURRENT ONCOLOGY | Non English |  |  |  |  |
|  |  | 689 | Wu, Jie et al. | 2019 | Effect of comprehensive guided nursing on skin injury and quality of life of patients with breast cancer treated by radiotherapy | Chinese Journal of General Practice | Different Study Design |  |  |  |  |
|  |  | 690 | Xu, D. W., Li, Z. J., Leitner, U. & Sun, J | 2024 | Efficacy of Remote Cognitive Behavioural Therapy for Insomnia in Improving Health Status of Patients with Insomnia Symptoms: A Meta-analysis | COGNITIVE THERAPY AND RESEARCH | Duplicate |  |  |  |  |
|  |  | 691 | Xu, D. W., Li, Z. J., Leitner, U. & Sun, J | 2024 | Efficacy of Remote Cognitive Behavioural Therapy for Insomnia in Improving Health Status of Patients with Insomnia Symptoms: A Meta-analysis | COGNITIVE THERAPY AND RESEARCH | Different Study Design |  |  |  |  |
|  |  | 692 | Yanez, B., et al | 2023 | Effectiveness and implementation of an electronic health record-integrated digital health intervention for managing depressive symptoms in ambulatory oncology | Contemporary Clinical Trials | Different Study Design |  |  |  |  |
|  |  | 693 | Yanez, B., et al | 2020 | Brief culturally informed smartphone interventions decrease breast cancer symptom burden among Latina breast cancer survivors | PSYCHO-ONCOLOGY | Different Study Design |  |  |  |  |
|  |  | 694 | Yang, Y.S., Ryu, G.W., Choi, M. | 2019 | Methodological strategies for ecological momentary assessment to evaluate mood and stress in adult patients using mobile phones: Systematic review | JMIR mHealth and uHealth | Different Study Design |  |  |  |  |
|  |  | 695 | Ye, L. C., Mages, M. A., Jimison, H. B. & Patel, S. R | 2022 | Developing OurSleepKit: A Couple-focused mHealth Tool to Support Adherence to Positive Airway Pressure Treatment | BEHAVIORAL SLEEP MEDICINE | Different Study Design |  |  |  |  |
|  |  | 696 | Yeboah-Asiamah, B., Yirenya-Tawiah, D., Baafi, D. & Ackumey, M. M | 2017 | Perceptions and knowledge about prostate cancer and attitudes towards prostate cancer screening among male teachers in the Sunyani Municipality, Ghana | AFRICAN JOURNAL OF UROLOGY | Different Study Design |  |  |  |  |
|  |  | 697 | Yehualashet, S. S. et al | 2021 | Predictors of adherence to COVID-19 prevention measure among communities in North Shoa Zone, Ethiopia based on health belief model: A cross-sectional study | PLoS ONE | Different Study Design |  |  |  |  |
|  |  | 698 | Yi, J. C., et al | 2025 | INteractive survivorship program to improve health care REsources [INSPIRE]: A study protocol testing a digital intervention with stepped care telehealth to improve outcomes for adolescent and young adult survivors | Contemporary Clinical Trials | Different Study Design |  |  |  |  |
|  |  | 699 | Yip, K. C., Lai, L. L., Ngu, S. T., Chong, R. S. T., Yahya, A. & See, M. H | 2023 | Exploring the health information-seeking practices of breast cancer patients in a middle-income country with a diverse ethnic population: a cross-sectional investigation | Supportive Care in Cancer | Non Original Articles |  |  |  |  |
|  |  | 700 | Yoo, W., Shah, D. V., Chih, M. Y. & Gustafson, D. H | 2020 | A smartphone-based support group for alcoholism: Effects of giving and receiving emotional support on coping self-efficacy and risky drinking | Health Informatics Journal | Different Study Design |  |  |  |  |
|  |  | 701 | Yuan, Q., Oginni, J., Liao, N., He, H. & Gao, Z | 2024 | Promoting precision health using fitness wearable and apps among breast cancer survivors: Protocols of a smart health management trial | Contemporary Clinical Trials | Different Study Design |  |  |  |  |
|  |  | 702 | Yussof, I. et al | 2024 | Challenges in Obtaining and Seeking Information Among Breast Cancer Survivors in an Asian Country: a Qualitative Study | Journal of Cancer Education | Duplicate |  |  |  |  |
|  |  | 703 | Yussof, I. et al | 2024 | Challenges in Obtaining and Seeking Information Among Breast Cancer Survivors in an Asian Country: a Qualitative Study | Journal of Cancer Education | Different Study Design |  |  |  |  |
|  |  | 704 | Zhang, L. S. & Jiang, S. H | 2021 | Linking health information seeking to patient-centered communication and healthy lifestyles: an exploratory study in China | HEALTH EDUCATION RESEARCH | Different Study Design |  |  |  |  |
|  |  | 705 | Zhang, Y., Wen, N. N. & Chao, N. P | 2019 | Effects of mobile information-seeking on the intention to obtain reproductive cancer screening among chinese women: testing an integrative model | CHINESE JOURNAL OF COMMUNICATION | Different Study Design |  |  |  |  |
|  |  | 706 | Zhou, J. J. & Wang, C. Y | 2020 | Improving cancer survivors' e-health literacy via online health communities (OHCs): a social support perspective | Journal of Cancer Survivorship | Different Study Design |  |  |  |  |
|  |  | 707 | Zhou, K., Wang, W., Zhao, W., Li, L., Zhang, M., Guo, P., Zhou, C., Li, M., An, J., Li, J. & Li, X | 2020 | Benefits of a WeChat-based multimodal nursing program on early rehabilitation in postoperative women with breast cancer: A clinical randomized controlled trial | International Journal of Nursing Studies | Duplicate |  |  |  |  |
|  |  | 708 | Zhou, K. N., Wang, W., Zhao, W. Q., Li, L. L., Zhang, M. Y., Guo, P. L., Zhou, C., Li, M. J., An, J. H., Li, J. & Li, X. M | 2020 | Benefits of a WeChat-based multimodal nursing program on early rehabilitation in postoperative women with breast cancer: A clinical randomized controlled trial | International Journal of Nursing Studies | Different Study Design |  |  |  |  |
|  |  | 709 | Zhu, J., Ebert, L., Guo, D., Yang, S., Han, Q. & Chan, S. W. C | 2018 | Mobile breast cancer e-support program for Chinese women with breast cancer undergoing chemotherapy (Part 1): Qualitative study of women’s perceptions | JMIR mhealth and uHealth | Different Study Design |  |  |  |  |
|  |  | 710 | Zhu, J., Ebert, L., Liu, X. & Chan, S. W | 2017 | A mobile application of breast cancer e-support program versus routine Care in the treatment of Chinese women with breast cancer undergoing chemotherapy: study protocol for a randomized controlled trial | BMC Cancer | Duplicate |  |  |  |  |
|  |  | 711 | Zhu, J., Ebert, L., Liu, X. & Chan, S. W. C | 2017 | A mobile application of breast cancer e-support program versus routine Care in the treatment of Chinese women with breast cancer undergoing chemotherapy: Study protocol for a randomized controlled trial | BMC Cancer | Different Study Design |  |  |  |  |
|  |  | 712 | Zhu, J., Ebert, L. & Wai-Chi Chan, S | 2017 | Integrative Review on the Effectiveness of Internet-Based Interactive Programs for Women With Breast Cancer Undergoing Treatment | Oncology Nursing Forum | Different Study Design |  |  |  |  |
|  |  | 713 | Zhu, J., Ebert, L., Xue, Z., Shen, Q. & Chan, S. W. C | 2017 | Development of a mobile application of Breast Cancer e-Support program for women with breast cancer undergoing chemotherapy | Technology and Health Care | Different Study Design |  |  |  |  |
|  |  | 714 | Zhu, J. M., Ebert, L., Guo, D. M., Yang, S. M., Han, Q. Y. & Chan, S. W. C | 2018 | Mobile Breast Cancer e-Support Program for Chinese Women With Breast Cancer Undergoing Chemotherapy (Part 1): Qualitative Study of Women's Perceptions | JMIR mHealth and uHealth | Different Study Design |  |  |  |  |
|  |  | 715 | Zhu, J. M., Ebert, L., Liu, X. Y. & Chan, S. W. C | 2017 | A mobile application of breast cancer e-support program versus routine Care in the treatment of Chinese women with breast cancer undergoing chemotherapy: study protocol for a randomized controlled trial | BMC Cancer | Different Study Design |  |  |  |  |
|  |  | 716 | Zhu, J. M., Ebert, L., Xue, Z. M., Shen, Q. & Chan, S. W. C | 2017 | Development of a mobile application of Breast Cancer e-Support program for women with breast cancer undergoing chemotherapy | Technology and Health Care | Different Study Design |  |  |  |  |
|  |  | 717 | Ziebland, S. & Wyke, S | 2012 | Health and Illness in a Connected World: How Might Sharing Experiences on the Internet Affect People's Health? | The Milbank Quarterly | Different Study Design |  |  |  |  |
|  |  | 718 | Zion, S. R., Schapira, L., Berek, J. S., Spiegel, D., Dweck, C. S. & Crum, A. J | 2023 | Changing cancer mindsets: A randomized controlled feasibility and efficacy trial | PSYCHO-ONCOLOGY | Different Study Design |  |  |  |  |
